# Global, regional, and national burden of osteoarthritis, 1990–2020 and projections to 2050: a systematic analysis for the Global Burden of Disease Study 2021

**DOI:** 10.1016/S2665-9913(23)00163-7

**Published:** 2023-08-21

**Authors:** GBD 2021 Osteoarthritis Collaborators, Jaimie D Steinmetz, Garland T Culbreth, Lydia M Haile, Quinn Rafferty, Justin Lo, Kai Glenn Fukutaki, Jessica A Cruz, Amanda E Smith, Stein Emil Vollset, Peter M Brooks, Marita Cross, Anthony D Woolf, Hailey Hagins, Mohsen Abbasi-Kangevari, Aidin Abedi, Ilana N Ackerman, Hubert Amu, Benny Antony, Jalal Arabloo, Aleksandr Y Aravkin, Ayele Mamo Argaw, Anton A Artamonov, Tahira Ashraf, Amadou Barrow, Lindsay M Bearne, Isabela M Bensenor, Alemshet Yirga Berhie, Nikha Bhardwaj, Pankaj Bhardwaj, Vijayalakshmi S Bhojaraja, Ali Bijani, Paul Svitil Briant, Andrew M Briggs, Nadeem Shafique Butt, Jaykaran Charan, Vijay Kumar Chattu, Flavia M Cicuttini, Kaleb Coberly, Omid Dadras, Xiaochen Dai, Lalit Dandona, Rakhi Dandona, Katie de Luca, Edgar Denova-Gutiérrez, Samath Dhamminda Dharmaratne, Meghnath Dhimal, Mostafa Dianatinasab, Karsten E Dreinhoefer, Muhammed Elhadi, Umar Farooque, Hamid Reza Farpour, Irina Filip, Florian Fischer, Marisa Freitas, Balasankar Ganesan, Belete Negese Belete Gemeda, Tamiru Getachew, Seyyed-Hadi Ghamari, Ahmad Ghashghaee, Tiffany K Gill, Mahaveer Golechha, Davide Golinelli, Bhawna Gupta, Veer Bala Gupta, Vivek Kumar Gupta, Rasool Haddadi, Nima Hafezi-Nejad, Rabih Halwani, Samer Hamidi, Asif Hanif, Netanja I Harlianto, Josep Maria Haro, Jan Hartvigsen, Simon I Hay, Jeffrey J Hebert, Golnaz Heidari, Mohammad-Salar Hosseini, Mehdi Hosseinzadeh, Alexander Kevin Hsiao, Irena M Ilic, Milena D Ilic, Louis Jacob, Ranil Jayawardena, Ravi Prakash Jha, Jost B Jonas, Nitin Joseph, Himal Kandel, Ibraheem M Karaye, Md Jobair Khan, Yun Jin Kim, Ali-Asghar Kolahi, Oleksii Korzh, Rajasekaran Koteeswaran, Vijay Krishnamoorthy, G Anil Kumar, Narinder Kumar, Sang-woong Lee, Stephen S Lim, Stany W Lobo, Giancarlo Lucchetti, Mohammad-Reza Malekpour, Ahmad Azam Malik, Luiz Garcia Garcia Mandarano-Filho, Santi Martini, Alexios-Fotios A Mentis, Mohamed Kamal Mesregah, Tomislav Mestrovic, Erkin M Mirrakhimov, Awoke Misganaw, Reza Mohammadpourhodki, Ali H Mokdad, Sara Momtazmanesh, Shane Douglas Morrison, Christopher J L Murray, Hasan Nassereldine, Henok Biresaw Netsere, Sandhya Neupane Kandel, Mayowa O Owolabi, Songhomitra Panda-Jonas, Anamika Pandey, Shrikant Pawar, Paolo Pedersini, Jeevan Pereira, Amir Radfar, Mohammad-Mahdi Rashidi, David Laith Rawaf, Salman Rawaf, Reza Rawassizadeh, Seyed-Mansoor Rayegani, Daniela Ribeiro, Leonardo Roever, Basema Saddik, Amirhossein Sahebkar, Sana Salehi, Lidia Sanchez Riera, Francesco Sanmarchi, Milena M Santric-Milicevic, Saeed Shahabi, Masood Ali Shaikh, Elaheh Shaker, Mohammed Shannawaz, Rajendra Sharma, Saurab Sharma, Jeevan K Shetty, Rahman Shiri, Parnian Shobeiri, Diego Augusto Santos Silva, Ambrish Singh, Jasvinder A Singh, Surjit Singh, Søren T Skou, Helen Slater, Mohammad Sadegh Soltani-Zangbar, Antonina V Starodubova, Arash Tehrani-Banihashemi, Sahel Valadan Tahbaz, Pascual R Valdez, Bay Vo, Linh Gia Vu, Yuan-Pang Wang, Seyed Hossein Yahyazadeh Jabbari, Naohiro Yonemoto, Ismaeel Yunusa, Lyn M March, Kanyin Liane Ong, Theo Vos, Jacek A Kopec

## Abstract

**Background:**

Osteoarthritis is the most common form of arthritis in adults, characterised by chronic pain and loss of mobility. Osteoarthritis most frequently occurs after age 40 years and prevalence increases steeply with age. WHO has designated 2021–30 the decade of healthy ageing, which highlights the need to address diseases such as osteoarthritis, which strongly affect functional ability and quality of life. Osteoarthritis can coexist with, and negatively effect, other chronic conditions. Here we estimate the burden of hand, hip, knee, and other sites of osteoarthritis across geographies, age, sex, and time, with forecasts of prevalence to 2050.

**Methods:**

In this systematic analysis for the Global Burden of Disease Study, osteoarthritis prevalence in 204 countries and territories from 1990 to 2020 was estimated using data from population-based surveys from 26 countries for knee osteoarthritis, 23 countries for hip osteoarthritis, 42 countries for hand osteoarthritis, and US insurance claims for all of the osteoarthritis sites, including the other types of osteoarthritis category. The reference case definition was symptomatic, radiographically confirmed osteoarthritis. Studies using alternative definitions from the reference case definition (for example self-reported osteoarthritis) were adjusted to reference using regression models. Osteoarthritis severity distribution was obtained from a pooled meta-analysis of sources using the Western Ontario and McMaster Universities Arthritis Index. Final prevalence estimates were multiplied by disability weights to calculate years lived with disability (YLDs). Prevalence was forecast to 2050 using a mixed-effects model.

**Findings:**

Globally, 595 million (95% uncertainty interval 535–656) people had osteoarthritis in 2020, equal to 7·6% (95% UI 6·8–8·4) of the global population, and an increase of 132·2% (130·3–134·1) in total cases since 1990. Compared with 2020, cases of osteoarthritis are projected to increase 74·9% (59·4–89·9) for knee, 48·6% (35·9–67·1) for hand, 78·6% (57·7–105·3) for hip, and 95·1% (68·1–135·0) for other types of osteoarthritis by 2050. The global age-standardised rate of YLDs for total osteoarthritis was 255·0 YLDs (119·7–557·2) per 100 000 in 2020, a 9·5% (8·6–10·1) increase from 1990 (233·0 YLDs per 100 000, 109·3–510·8). For adults aged 70 years and older, osteoarthritis was the seventh ranked cause of YLDs. Age-standardised prevalence in 2020 was more than 5·5% in all world regions, ranging from 5677·4 (5029·8–6318·1) per 100 000 in southeast Asia to 8632·7 (7852·0–9469·1) per 100 000 in high-income Asia Pacific. Knee was the most common site for osteoarthritis. High BMI contributed to 20·4% (95% UI –1·7 to 36·6) of osteoarthritis. Potentially modifiable risk factors for osteoarthritis such as recreational injury prevention and occupational hazards have not yet been explored in GBD modelling.

**Interpretation:**

Age-standardised YLDs attributable to osteoarthritis are continuing to rise and will lead to substantial increases in case numbers because of population growth and ageing, and because there is no effective cure for osteoarthritis. The demand on health systems for care of patients with osteoarthritis, including joint replacements, which are highly effective for late stage osteoarthritis in hips and knees, will rise in all regions, but might be out of reach and lead to further health inequity for individuals and countries unable to afford them. Much more can and should be done to prevent people getting to that late stage.

**Funding:**

Bill & Melinda Gates Foundation, Institute of Bone and Joint Research, and Global Alliance for Musculoskeletal Health.

## Introduction

Osteoarthritis is the most prevalent form of arthritis and is a leading cause of adult chronic pain and long-term disability.^1^, ^2^, ^3^ Osteoarthritis most commonly affects the hip, knee, and hand joints, but most joints can be involved. Osteoarthritis is a major source of health expenditure. In the USA in 2016, for example, osteoarthritis was responsible for an estimated US$80 billion in health-care spending,^4^ and in Hong Kong in 2003, osteoarthritis was responsible for more than $400 million in direct and indirect spending.^5^ The 2015 WHO Global Ageing and Health Report^6^ highlights osteoarthritis as a leading cause of disability in adults aged 60 years and older. Given that global populations are ageing, the health and economic burden of osteoarthritis is increasing. WHO designated 2021–30 to be the decade of healthy ageing, with an emphasis not only on life expectancy but also quality of life. This designation provides an opportunity to focus on osteoarthritis burden in the context of adult health,^7^ especially given the chronic nature of osteoarthritis and its effect on mobility and daily activities. Osteoarthritis can manifest relatively early in adulthood, including in people younger than 50 years,^8^ and therefore, preventing or mitigating the effects of osteoarthritis could avoid decades of reduced quality of life.

**Research in context**

**Evidence before this study**

The Global Burden of Disease (GBD) study is the only source of global, regional, and country estimates of osteoarthritis burden over time. We have previously reported on osteoarthritis of the hip and of the knee, but here we also report on osteoarthritis of the hand and of other sites for the first time. Input data for GBD osteoarthritis models were identified through a systematic review and use of US insurance-claims data. We did a systematic review of population-based epidemiological studies of osteoarthritis reporting on osteoarthritis prevalence published between 1980 and the end of 2019 in PubMed using the search terms ((“osteoarthritis” AND (“epidemiology” OR “prevalence”)) AND “humans”) AND (“population” OR “population groups” OR (“population” AND “groups”)).

**Added value of this study**

This Article provides an update on the global prevalence and burden of osteoarthritis to the year 2020. This GBD iteration includes new statistical methods used to adjust for non-reference data and two new sites of osteoarthritis—hand and an other category (eg, shoulder or elbow). We also, to the best of knowledge for the first time, present forecasts of case numbers at the global and regional level to the year 2050 for total osteoarthritis and by each individual site, and report on population-attributable risk of high BMI for osteoarthritis. By 2050, we estimate that case numbers will increase by 60% to 100% depending on the site of osteoarthritis. High BMI had population-attributable fractions of 20·4% for osteoarthritis, making this an important modifiable risk factor.

**Implications of all the available evidence**

Given that the 2021–30 decade has been designated the decade of healthy ageing by WHO, and that osteoarthritis is a major cause of adult disability, an up-to-date analysis of temporal, age-related, geographical, and site-specific trends in osteoarthritis burden can benefit policy decisions. If trends continue as at present, we estimate that almost 1 billion individuals will have some form of osteoarthritis in the year 2050. Because there is no known cure for osteoarthritis and no proven structure-modifying interventions, this burden represents a major challenge to health systems. Population and health-systems approaches adaptable to all regions must address osteoarthritis at all stages from prevention strategies addressing modifiable risk factors to multimodal integrated education and care to improve pain and disability, and timely and equitable access to joint-replacement surgery. These data provide strong support to address high BMI as a modifiable risk factor to reduce osteoarthritis burden. Health system planning would be further aided by quantifying additional osteoarthritis risk factors, such as injury and occupational hazards. The presented evidence should contribute to developing global health initiatives for osteoarthritis during the 2021–30 decade of healthy ageing.

Current management strategies for osteoarthritis include exercise and other forms of physical therapy, assistive devices such as canes or splints, home modifications, self-management educational programmes, pain medication, and surgical treatments including joint replacement.^9^ Established modifiable risk factors for osteoarthritis include high BMI^10^ and joint injury.^11^, ^12^ Other factors that increase the risk of osteoarthritis are physically demanding occupations,^13^ elite-level high-impact sports,^14^, ^15^ surgery such as meniscectomy following injury,^16^ joint anatomy, and muscle weakness.^3^ To effectively target prevention and intervention, we need to quantify the sex-specific, age-specific, and location-specific patterns of the prevalence and burden of total osteoarthritis and each site of osteoarthritis.

The Global Burden of Diseases, Injuries, and Risk Factors (GBD) Study systematically quantifies health loss for 369 diseases by age, sex, year, and geographical location, and allows for the comparison of burden across disparate diseases.^17^ Other assessments of osteoarthritis burden have been done in the past decade,^18^ but these assessments focus on individual osteoarthritis sites, single geographies such as Asia^19^ or Africa,^20^ subpopulations such as professional athletes,^14^ or risk factors such as high BMI,^10^ or provide forecasts for specific locations.^21^

In this study, we report the national, regional, and global burden of osteoarthritis, which updates the last osteoarthritis-specific report of estimates for GBD 2010^22^ and GBD 2017.^23^ We report prevalence and burden in 2020, trends from 1990 to 2020, contribution of high BMI to osteoarthritis, and we project osteoarthritis prevalence to 2050.

## Methods

### Overview

This manuscript was produced as part of the GBD Collaborator Network and in accordance with the GBD Protocol. The GBD Study adheres to the Guidelines for Accurate and Transparent Health Estimates Reporting statement. The methods presented here describe case definitions, data collection, and disease modelling methods. Studies done between 1980 and 2019 from all available global locations were identified using PubMed (appendix pp 3–4). These data were used to produce prevalence estimates for each osteoarthritis site between the years 1990 and 2020, across the age range (>30 years), for male and female sexes, and for all GBD locations (appendix pp 1–2 presents a description of GBD super- regions, regions, and country groupings).

Input data sources used for osteoarthritis models are listed in the appendix (pp 38–51). Many of these studies are Community Oriented Program for the Control of Rheumatic Diseases (COPCORD) studies and applied a common methodological framework to ascertain the scope of musculoskeletal conditions in more than 30 countries in the Americas, Africa, Australasia, and the Middle East.

### Case definitions

The GBD reference case definition for both hip and knee osteoarthritis is symptomatic osteoarthritis radiographically confirmed as Kellgren-Lawrence grade 2–4 (definitive osteoarthritis).^24^, ^25^, ^26^ Kellgren-Lawrence grade 2 indicates the presence of one defined osteophyte in the joint, grade 3 indicates the presence of several osteophytes and joint-space narrowing, and grade 4 indicates the same criteria as grade 3 in addition to bone deformity. Symptomatic osteoarthritis requires reported pain for at least 1 month out of the past 12 months.

The main sources of input data for the hip and knee osteoarthritis models were cross-sectional, population-based survey data from locations worldwide and state-level US insurance claims data captured by International Classification of Diseases (ICD)-9 four-digit or five-digit codes starting with 715 specific to knee or hip, and ICD-10 codes M16 and M17. ICD-10 used the terminology osteoarthritis and replaced ICD-9 that used osteoarthrosis to reflect the recognition that inflammatory processes are involved in the pathogenesis of osteoarthritis.

Two new osteoarthritis categories were added in GBD 2019, comprising hand osteoarthritis and a residual category of other osteoarthritis sites (eg, shoulder and elbow). Cases of osteoarthritis affecting the cervical spine, lumbar spine, or both sites were excluded from the ‘other osteoarthritis’ category, because pain in these sites is captured by the neck pain and lower back pain disease categories in the GBD Study.^17^ Existing osteoarthritis input data from previous GBD hip and knee modelling were rereviewed for mention of osteoarthritis present in the hand or other joints. In addition, we did a broad systematic review of epidemiological studies of osteoarthritis prevalence published in English between 1980 and end of 2019 in PubMed (appendix p 3).

Eight diagnostic criteria, six hand joints, and more than 20 combinations of different hand joints were reported in the literature. Affected hand joints were either evaluated independently, in some explicit combination (eg, the distal interphalangeal joints [DIP] and proximal interphalangeal joints [PIP]), or as generalised osteoarthritis, wherein a case had osteoarthritis present in the DIP, PIP, and first carpometacarpal joints (CMC) specifically. Other sources reported estimates of hand osteoarthritis present in any single joint or present in several joints without specifying joint type. We classified the case definitions of hand osteoarthritis in terms of the two dimensions of diagnostic criteria (presence of symptoms and diagnosis with or without radiography) and four categories of affected sites (appendix p 5). Consistent with hip and knee osteoarthritis modelling, symptomatic, radiographically confirmed osteoarthritis in any single joint of the hand was used as the reference case definition for hand osteoarthritis, to which alternative case definitions present in the literature were adjusted.

Given the paucity of survey data on other osteoarthritis joint sites, US insurance claims data from 2000 to 2016 constituted the sole source of other osteoarthritis data. Claims data cases of other osteoarthritis were identified using codes under ICD-10 M19 for cases of other and unspecified osteoarthritis that were not used in estimation of osteoarthritis hip, knee, or hand.

### Data processing and disease modelling

Before fitting models, data reported for male and female sexes combined were split by sex, non-reference osteoarthritis definition data (eg, identified by self-reporting alone, radiography alone, or in US claims data) were adjusted to the reference case definition, and wide age-range data were split by age into smaller bins (sex and age split details in the appendix p 4). A 2011 systematic review found that different case definitions could lead to variable prevalence estimates across osteoarthritis sites.^27^ Data that were ascertained using different diagnostic criteria, such as self-reporting or ICD coding in insurance claims data, were adjusted for systematic bias, a process referred to as crosswalking. Adjustment factors were estimated by pairing studies on the basis of location, sex, and age, then using a tool developed at the Institute for Health Metrics and Evaluation (the meta-regression—Bayesian, regularised, trimmed tool)^28^ to do a meta-analysis of the estimated logit difference between prevalence reported by alternate case definition and prevalence reported by the reference definition (adjustment factors in the appendix pp 5–7). Meta-analysis results systematically adjusted insurance-claims-data prevalence upward for osteoarthritis in the hip, knee, and hand, which agrees with previously published work demonstrating undercounting in ICD-coded data.^29^

Bayesian meta-regressions of the adjusted data were run using DisMod-MR 2.1,^17^ an age-integrating Bayesian meta-regression log-normal disease model with a mixed-effects geographical cascade. The meta-regression was a combination of a meta-analysis to pool data points with weighted averages to include and reconcile heterogeneous data, and a regression to include known associations between several variables (eg, osteoarthritis prevalence and BMI or age). Fixed effects included sex and country-level covariates (eg, BMI). Nested random effects were calculated for each super-region, region, and country. DisMod-MR 2.1 is a compartmental model and solves differential equations to ensure consistency between different parameters; in the case of osteoarthritis, incidence and prevalence. Incidence of osteoarthritis was set to zero before age 30 years. The age-standardised osteoarthritis summary exposure value (SEV) scalar (a normalised value of risks affecting a disease) and BMI were included as covariates on prevalence in the models for hip, knee, and other types of osteoarthritis. High BMI was the only risk factor included in the current GBD Study with osteoarthritis as an outcome. The SEV was not used in the hand osteoarthritis model, given the absence of strong evidence suggesting a relationship between BMI and osteoarthritis in hand joints. First, a global model included all data from all years to produce an initial global fit and to calculate covariate coefficients and location random effects. Next, the global fit adjusted by covariate coefficients and random effects was passed as data (a prior) to super-region models to help inform model estimates, and this process was repeated for each level of the geographical cascade. Final estimates were produced by then aggregating up the geographical cascade; in other words, final prevalence estimates for each region were the aggregation of the prevalence of all countries within the region. DisMod models were iterated 5000 times using a Markov chain Monte Carlo algorithm. Uncertainty bounds reflect stochastic error, measurement error, and between-study heterogeneity.

The GBD Study estimated osteoarthritis prevalence in all countries. For most disease models in GBD, input data were not available for every location where we estimated prevalence. In these cases, prevalence estimates in DisMod were made through two main mechanisms: through regional priors and country-level covariates. Regional estimates used data from all countries in a given GBD region to produce regional estimates, and these priors were passed down to each country in the region to help inform country estimates. In regions with no data, estimates were informed by super-region priors. Country-specific mean BMI also informed estimates in countries with no data.

Prevalence estimates generated by DisMod-MR 2.1 were split into four severity levels:^17^ asymptomatic; mild; moderate; and severe, on the basis of a pooled meta-analysis of five severity distribution sources from three GBD super-regions (high-income, south Asia, and southeast Asia, east Asia, and Oceania super-regions) that assessed osteoarthritis severity using the Western Ontario and McMaster Universities Arthritis Index.^30^ One of these data sources was the large database of knee osteoarthritis provided by the Osteoarthritis Initiative.^31^ Disability weights for mild, moderate, and severe osteoarthritis were generated using population-based surveys in which participants were asked to compare the severity of sets of health states.^32^ Prevalence at each severity level was then multiplied by the corresponding disability weight of the health state to calculate years lived with disability (YLDs; appendix pp 7–8). Comorbidity-adjusted YLDs were generated by simulating the distribution of all conditions and health states in the GBD cause hierarchy at the severity level and for each location-year, assuming independence. Because the prevalence of osteoarthritis in several sites was counted as one prevalent case per person without accounting for site-specific correlation, the combined prevalence of osteoarthritis in unique sites exceeded the total number of cases of osteoarthritis (referred to as total osteoarthritis). No death was attributed to osteoarthritis in the GBD Study, and therefore this Article provides only non-fatal measures of burden (ie, YLDs).

Prevalence and YLDs are presented as either counts (prevalent cases or number of YLDs), or rates (prevalent cases per 100 000 people or YLDs per 100 000 people, at all ages, when age standardised, or when age specific). For both prevalence and YLDs, mean and uncertainty were calculated by taking the final 100 outputs from the posterior distribution after model convergence (termed draws), collapsing to the mean and 95% uncertainty intervals (UIs) as the 2·5th and 97·5th ordered draws. Age-standardised rates were calculated using GBD standard population age weights.^17^

### Risk estimation for high BMI

High BMI is a GBD risk factor, defined as a BMI of more than a theoretical minimum risk level that ranges between 20 kg/m^2^ and 25 kg/m^2^ in adults aged 20 years or older. High BMI data and methods are described in detail elsewhere.^33^ Briefly, population exposure to high BMI was calculated for each country, age, sex, and year using a combination of spatiotemporal Gaussian process regression and mixed-effects models. High BMI was established as a risk factor for hip and knee osteoarthritis by performing a systematic review of published analyses of cohort studies and use of causal criteria to examine strength of the evidence. The cohort studies were used to determine the relative risk of osteoarthritis incidence for every five-unit increase in BMI. The relative risk estimate was then used to calculate the population-attributable risk. Population-attributable fractions for osteoarthritis caused by high BMI were calculated as the expected reduction in osteoarthritis if the exposure of high BMI was decreased to the theoretical minimum risk level. It is important to note that a negative lower bound for population-attributable fractions does not signify a protective effect, but instead signifies the absence of a relationship.

### Estimate projections

The number of global and regional cases of osteoarthritis were estimated to the year 2050, using forecasted population estimates^34^ and a regression to forecast prevalence that included the Socio-demographic Index (SDI) as a predictor. For each osteoarthritis site and for total osteoarthritis, age-specific, location-specific, and sex-specific GBD 2019 prevalence rates for all estimation years were logit transformed and used in the following regression model:

$$E\left[logit\left(Y_{l,a,s,y}\right)\right]=\beta_{1}SD1+\alpha_{l,a,s}$$

In this linear model fitted to logit-transformed prevalence, *E[logit(Y*_l,a,s,y_*)]* is the forecasted logit (prevalence) estimated by (l,a,s,y), the unique location-age-sex-year, in which β_1_ is the fixed coefficient on SDI over time and α_l,a,s_ is the location-age-sex-specific random intercept. To compute forecasted cases, forecasted rates were multiplied by forecasted population counts.^34^ Forecasted prevalence rates were intercept shifted to GBD prevalence in the year 2021, and this difference was used to shift all forecasted values to the year 2050. A validation experiment was used to forecast prevalence for the years 2010–19 and compared these projected results to the known 2010–19 prevalence estimates (appendix p 8). A Das Gupta decomposition analysis was done to determine the relative contributions to the change in case number between 2020 and 2050 of population growth, population ageing, and changes in prevalence unrelated to demographics.^35^

### Role of the funding source

The funder of the study had no role in the study design, data collection, data analysis, data interpretation, or writing of the report.

## Results

A total of 53 non-insurance-claims sources with prevalence or incidence data covering 22 countries in nine of 21 GBD regions and six of seven GBD super-regions were included for hip osteoarthritis estimation. A total of 95 sources covering 26 countries in 12 regions and all seven super-regions were included for knee osteoarthritis. A total of 69 sources covering 12 countries in eight regions and six super-regions were included for hand osteoarthritis. Hand, hip, and knee osteoarthritis models all included US insurance claims data by state, which comprised more than 600 sources for each model (appendix pp 10–15). Only US insurance claims data were used to estimate ‘other’ osteoarthritis, for a total of 624 sources (appendix p 16).

In 2020, an estimated 7·6% (95% UI 6·8–8·4) of the global population lived with osteoarthritis, some 595 million (95% UI 535–656) individuals. In 1990, 4·8% (4·3–5·3) of the global population had osteoarthritis, equating to 256 million (232–282) individuals, and all-age prevalence steadily increased in the intervening decades. Of the global population aged 30 years or older in 2020, 14·8% (13·3–16·3) lived with some form of osteoarthritis, and in working-age adults aged 30–60 years, 3·5% (3·1–3·9) lived with some form of osteoarthritis.

Between 1990 and 2020, the global age-standardised rate of YLDs for total osteoarthritis increased by 9·5% (95% UI 8·6–10·1), from 233·0 (109·3–510·8) to 255·0 (119·7–557·2) YLDs per 100 000 (table; appendix pp 17–18), and ranked as the 14th most common cause of age-standardised YLDs when compared to other diseases at level 3 of the GBD cause hierarchy. For adults aged 70 years and older, osteoarthritis was the seventh ranked cause of YLDs in 2020 and sixth ranked in 1990. Increases in age-standardised YLD rates between 1990 and 2020 were observed for hip (6·0%, 4·2–7·3), knee (8·2%, 7·5–8·9), hand (14·1%, 12·9 to 15·1), and ‘other’ osteoarthritis (5·4%, 4·5–6·3). By comparison, the global number of YLDs for total osteoarthritis increased by 134·0% (131·8–136·0) from 9·28 million (4·34–20·3) in 1990 to 21·7 million (10·2–47·6) YLDs in 2020, and the all-age rate increased by 60·1% (58·6–61·4) from 173·6 YLDs (81·3–380·4) per 100 000 to 278·0 (130·6–608·3). By osteoarthritis site, all-age YLDs rates increased by 69·0% (67·0–71·1) for hand osteoarthritis, 56·3% (54·8–57·7) for other types of osteoarthritis, 56·9% (55·5–58·3) for knee osteoarthritis, and 55·8% (53·7–58·2) for hip osteoarthritis between 1990 and 2020.

**Table:** Number and age-standardised rate of YLDs in 2020 for total osteoarthritis, and percentage change from 1990 for each measure globally, and by GBD regions and super-regions

		**Number of YLDs**	**Percentage change in number of YLDs from 1990 to 2020**	**Age-standardised rate of YLDs per 100 000 in 2020**	**Percentage change in age-standardised rate of YLDs from 1990 to 2020**
Global		21 700 000 (10 200 000–47 600 000)	134·0% (131·8–136·0)	255·0 (119·7–557·2)	9·5% (8·6–10·1)
Central Europe, eastern Europe, and central Asia		1 750 000 (825 000–3 720 000)	43·7% (42·0–45·6)	278·1 (130·6–593·8)	8·1% (7·0–9·3)
	Central Asia	209 000 (98 100–446 000)	97·9% (93·6–102·5)	262·8 (123·6–557·7)	16·3% (14·3–18·7)
	Central Europe	530 000 (249 000–1 130 000)	57·3% (55·1–60·0)	257·4 (120·6–549·4)	12·5% (11·4–13·9)
	Eastern Europe	1 010 000 (477 000–2 160 000)	30·4% (28·3–32·6)	294·6 (138·3–631·6)	6·0% (4·5–7·2)
High income		5 800 000 (2 740 000–12 500 000)	81·3% (79·4–82·9)	295·1 (138·9–641·6)	7·5% (7·0–8·3)
	Australasia	140 000 (66 100–309 000)	128·9% (122·0–134·9)	295·2 (138·8–651·9)	11·0% (7·7–13·8)
	High-income Asia Pacific	1 330 000 (623 000–2 880 000)	112·5% (107·4–118·2)	329·7 (154·5–723·3)	8·2% (6·8–9·6)
	High-income North America	1 890 000 (901 000–4 080 000)	88·3% (86·1–90·8)	314·0 (148·9–680·4)	4·9% (4·0–6·0)
	Southern Latin America	238 000 (112 000–520 000)	98·2% (93·3–103·5)	285·4 (133·9–624·4)	9·9% (7·6–12·6)
	Western Europe	2 200 000 (1 030 000–4 720 000)	58·5% (56·7–60·1)	264·5 (124·2–573·3)	6·0% (4·9–6·9)
Latin America and Caribbean		1 680 000 (786 000–3 660 000)	209·1% (205·0–213·3)	272·9 (128·0–594·0)	13·6% (12·6–14·7)
	Andean Latin America	160 000 (74 700–353 000)	216·3% (210·5–223·6)	273·2 (127·4–601·6)	12·6% (10·9–14·9)
	Caribbean	140 000 (65 500–306 000)	124·8% (120·8–128·0)	262·8 (122·9–573·9)	10·1% (8·1–11·7)
	Central Latin America	685 000 (321 000–1 490 000)	230·8% (225·6–236·4)	276·3 (129·5–600·8)	14·1% (12·5–15·8)
	Tropical Latin America	691 000 (325 000–1 500 000)	210·9% (206·4–215·8)	271·6 (127·8–589·7)	13·9% (12·7–15·0)
North Africa and Middle East		1 070 000 (498 000–2 320 000)	215·2% (210·4–219·6)	224·5 (105·5–490·2)	17·2% (15·5–18·7)
South Asia		3 360 000 (1 570 000–7 370 000)	199·9% (193·4–206·8)	225·8 (105·8–494·2)	18·9% (16·8–20·9)
Southeast Asia, east Asia, and Oceania		7 000 000 (3 270 000–15 300 000)	186·0% (177·7–192·5)	242·4 (113·2–528·7)	16·2% (13·9–18·5)
	East Asia	5 630 000 (2 640 000–12 300 000)	183·0% (174·5–190·3)	254·3 (118·9–554·5)	15·5% (12·9–18·0)
	Oceania	17 500 (8 130–37 400)	177·9% (169·4–184·5)	222·0 (103·8–482·2)	10·8% (7·4–13·3)
	Southeast Asia	1 350 000 (634 000–2 930 000)	199·9% (191·8–205·7)	204·3 (96·0–445·1)	19·9% (17·0–21·9)
Sub-Saharan Africa		1 090 000 (513 000–2 380 000)	156·3% (153·3–159·3)	219·9 (103·5–481·0)	11·6% (10·3–12·7)
	Central sub-Saharan Africa	124 000 (58 500–270 000)	163·6% (156·9–171·1)	212·9 (101·0–464·0)	6·6% (4·3–9·4)
	Eastern sub-Saharan Africa	367 000 (172 000–800 000)	165·3% (159·9–169·2)	209·3 (98·4–457·2)	15·5% (13·3–17·2)
	Southern sub-Saharan Africa	155 000 (73 200–335 000)	133·6% (131·1–136·8)	261·0 (123·6–561·1)	9·2% (8·1–10·6)
	Western sub-Saharan Africa	448 000 (209 000–977 000)	155·8% (151·8–158·9)	218·3 (102·4–480·7)	11·5% (10·0–12·6)

Data in parentheses are 95% uncertainty intervals. The sum of regional YLDs does not exactly match YLD cases because of rounding. North Africa and Middle East and South Asia are both super-regions and regions. YLDs=years lived with disability.

**Figure 1:**
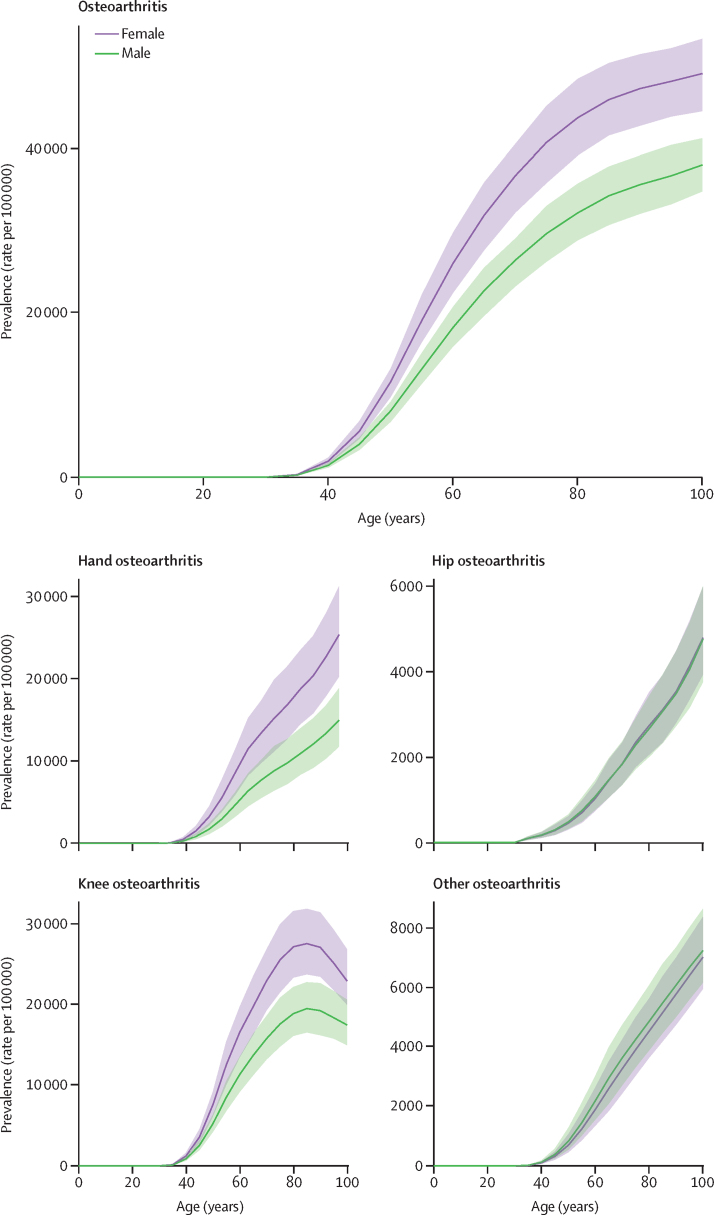
Global prevalence of total osteoarthritis and each site of osteoarthritis in 2020 by sex and age Shaded area represents 95% uncertainty intervals.

Across all estimation years, prevalence of osteoarthritis was more common in females than in males, with a 2020 global age-standardised prevalence of 8058·9 per 100 000 (95% UI 7251·9–8867·9) for females and 5780·1 per 100 000 (5217·8–6341·2) for males. Prevalence of total osteoarthritis increased with age; in 2020, the 70 and older age group had a prevalence rate of 38 418·9 per 100 000 (34 471·4–42 302·7), and the group aged 50–69 years had a prevalence rate of 23 237·2 per 100 000 (20 390·8–26 108·6). The rate in younger adults aged 25–49 years was 2983·5 per 100 000 (2513·1–3439·1). Temporal trends in age-specific and all-age prevalence of total osteoarthritis are shown in the appendix (p 17). The global age-specific rates for individual sites of osteoarthritis increased with age, with the exception of knee osteoarthritis, for which the prevalence rate peaked at age 80–84 years and then began to decrease (figure 1). The knee was the most common site of osteoarthritis, with a 2020 global age-standardised prevalence of 4307·4 cases (3844·5–4913·3) per 100 000, followed by osteoarthritis of the hand (2226·1, 1719·7–2802·8), other types of osteoarthritis (718·4, 578·2–932·1), and osteoarthritis of the hip (417·7, 314·7–532·7; appendix p 19).

**Figure 2:**
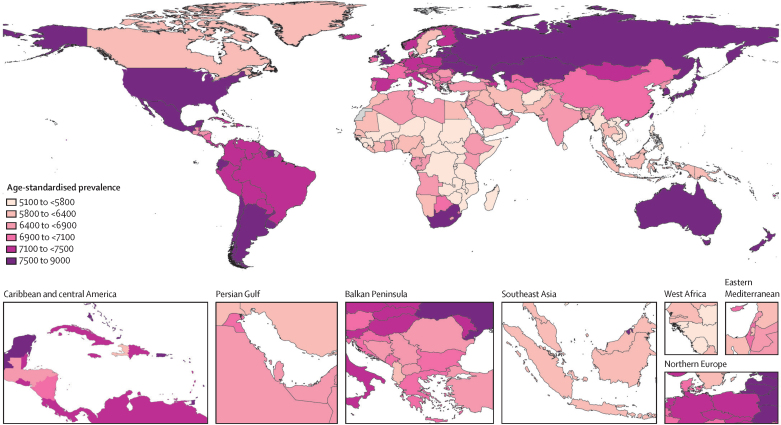
Age-standardised prevalence per 100 000 of total osteoarthritis by country for male and female sexes combined in 2020

Among GBD regions, the age-standardised prevalence of total osteoarthritis was greatest in high-income Asia Pacific (8632·7 per 100 000, 95% UI 7852·0–9469·1), high-income North America (8431·7 per 100 000, 7676·2–9329·1), and eastern Europe (7937·9 per 100 000, 7013·5–8931·2), and was smallest in southeast Asia (5677·4 per 100 000, 5029·8–6318·1), eastern sub-Saharan Africa (5821·0 per 100 000, 5216·8–6438·8), and central sub-Saharan Africa (5946·0 per 100 000, 5340·7–6542·4; appendix pp 17–18)· Nationally, the USA had the highest age-standardised prevalence (8696·1 per 100 000, 7937·9–9610·2; figure 2). The all-age prevalence of total osteoarthritis was highest in high-income Asia Pacific (18 381·6 per 100 000, 16 734·2–20 177·2) and high-income North America (13 843·2 per 100 000, 12 555·1–15 296·6), and lowest in eastern sub-Saharan Africa (2419·7 per 100 000, 2184·7–2674·4) and central sub-Saharan Africa (2555·5 per 100 000, 2036·8–2822·5). Region and country-level prevalence by osteoarthritis site are provided in the appendix (pp 19–32).

**Figure 3:**
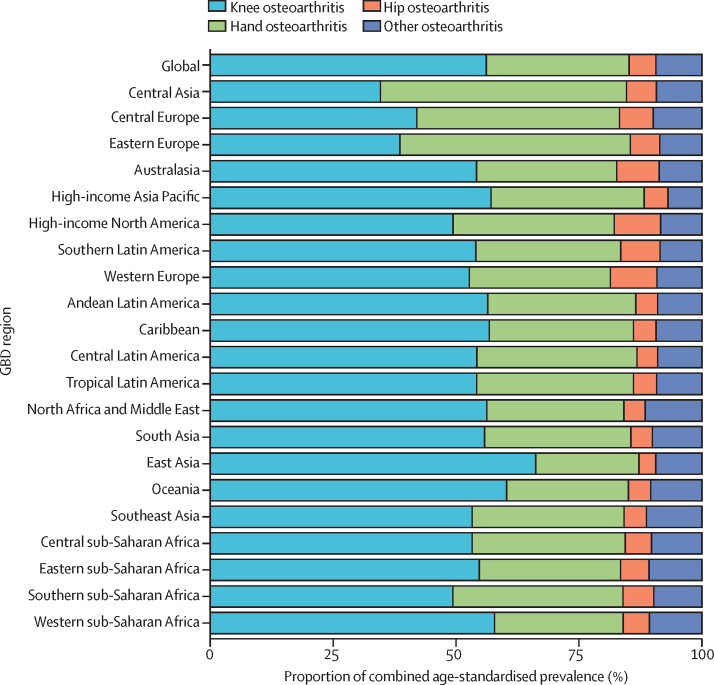
Contribution of different osteoarthritis sites to combined age-standardised prevalence, globally and by GBD region, 2020

Knee osteoarthritis was the largest contributor to combined osteoarthritis age-standardised prevalence in all GBD regions except for central Asia and eastern Europe, where hand osteoarthritis was the largest contributor (figure 3). The proportion of knee osteoarthritis ranged from 34·6% (95% UI 32·2–37·8) in central Asia to 66·2% (63·6–69·2) in east Asia. Hip osteoarthritis was the smallest contributor to osteoarthritis prevalence in all regions except for high-income North America and western Europe, where the contribution of other osteoarthritis was the same or slightly lower. The proportion of hip osteoarthritis ranged from 3·4% (3·1–3·6) in east Asia to 9·5% (8·6–10·3) in high-income North America. The contribution of hand osteoarthritis varied widely by region, with a minimum contribution in east Asia of 21·0% (18·9–22·6) to a maximum contribution in central Asia of 50·0% (47·2–51·4).

High BMI was the only GBD risk factor for osteoarthritis, with a 2020 global age-standardised population-attributable fraction of 20·4% (–1·7 to 36·6). High BMI was responsible for 4·6 million (–0·317 to 15·0) osteoarthritis YLDs. The global contribution of high BMI to osteoarthritis increased over time, with a 1990 age-standardised population-attributable fraction of 16·1% (–1·3 to 30·2). The 2020 global age-standardised population-attributable fractions in females was 21·1% (–1·8 to 37·7) and in males was 19·3% (–1·7 to 35·3). The population-attributable fraction was highest in southern Latin America (27·9%, –2·4 to 47·7) and lowest in south Asia (12·7%, –0·9 to 25·2).

**Figure 4:**
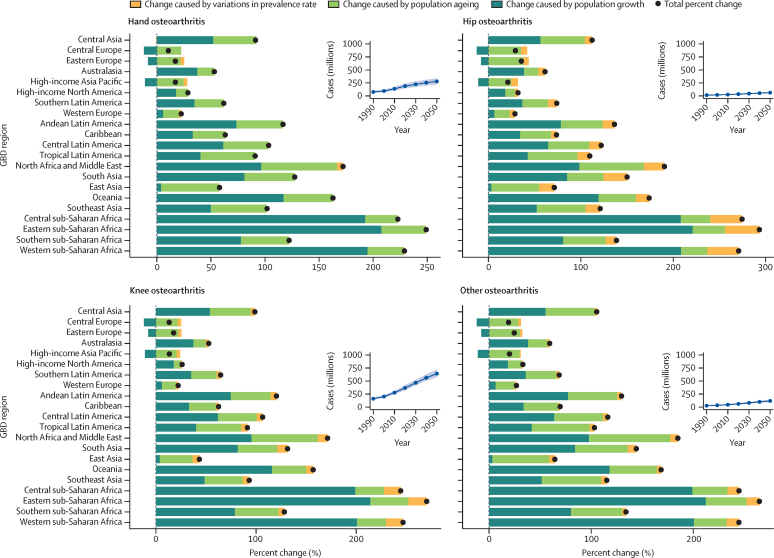
Global cases of site-specific osteoarthritis forecasted to the year 2050 and decomposition analysis of relative contribution of change in prevalence rate, population growth, and population ageing to total percent change in age-restricted case number by region, 2020–50 Insets display global case numbers for each osteoarthritis site. Shading denotes 95% uncertainty intervals.

In 2050, there will be an estimated 642 million (95% UI 574–722) individuals with knee osteoarthritis, 279 million (221–338) individuals with hand osteoarthritis, 62·6 million (49·7–75·5) individuals with hip osteoarthritis, and 118 million (97·1–144) individuals with other types of osteoarthritis (figure 4). These numbers constitute increases in case numbers from 2020 to 2050 of 74·9% (59·4–89·9) for knee osteoarthritis, 48·6% (35·9–67·1) for hand osteoarthritis, 78·6% (57·7–105·3) for hip osteoarthritis, and 95·1% (68·1–135·0) for other types of osteoarthritis.

For every osteoarthritis site, the three regions with the lowest percentage change in all-age cases from 2020 to 2050 were central Europe, eastern Europe, and high-income Asia Pacific. The three regions with the greatest change were central sub-Saharan Africa, eastern sub-Saharan Africa, and western sub-Saharan Africa, which saw projected increases of more than 200% across all sites of osteoarthritis (figure 4; 2050 age-standardised prevalence and cases by osteoarthritis site are shown in the appendix pp 33–34).

A decomposition analysis by region shows the relative contribution of population growth, population ageing, and changes in prevalence to the projected increase in cases (figure 4; appendix pp 35–37). Population growth was the largest contributor in most locations, especially in regions within sub-Saharan Africa. In central, eastern, and western sub-Saharan Africa, population growth accounted for more than a 200% increase in case numbers for all sites of osteoarthritis. Locations with the smallest percentage change in case numbers between 2020 and 2050 had a projected population decline rather than growth, which is the case for example in central and eastern Europe. In general, increases in age-standardised prevalence contributed less to the projected increase in cases than projected changes in demography.

## Discussion

We estimated the burden of total osteoarthritis and individual sites of osteoarthritis, including newly added models of hand osteoarthritis and other types of osteoarthritis in GBD 2019, at which time we did an updated systematic review for all osteoarthritis sites and implemented new methods to adjust non-reference data. Osteoarthritis was a top-ten leading cause of YLDs for adults older than 70 years in 2020, affecting one-third of adults in this age group, and ranked 14th for age-standardised YLDs across all ages. Although prevalence increases with age, 3·5% of working-aged adults aged 30–60 years experienced some form of osteoarthritis in 2020. Working-age and older adults with osteoarthritis are both important demographics to target for better access and impact of rehabilitation programmes.^36^

The pronounced increase between 1990 and 2020 in total global YLD counts and all-age YLD rate compared with the relatively stable age-standardised rate shows that the expanding burden of osteoarthritis is largely caused by the growth and ageing of populations.^37^ High BMI is currently the only risk factor for osteoarthritis for which attributable burden is quantified in the GBD. Given high BMI accounted for just 20% of osteoarthritis burden, it will be an important future direction to quantify the contribution of history of injuries,^8^, ^12^ occupational risk,^38^ prior joint-related surgeries, and other potentially modifiable risk factors to osteoarthritis burden.

Total osteoarthritis YLDs, as well as hand and knee osteoarthritis YLDs, were higher in females than males, even after accounting for demographics. The most common form of osteoarthritis was knee, and the least common was hip across the majority of GBD regions. We estimate that global case numbers for each site of osteoarthritis will increase by 48·6% to 95·1% between 2020 and 2050. Due to projected population growth, regions in sub-Saharan Africa have the largest projected increase in cases, more than 200% by 2050, which should be emphasised in health systems planning.

Age-standardised prevalence of osteoarthritis in 2020 was highest in high-income North America and high-income Asia Pacific, and lowest in southeast Asia and eastern sub-Saharan Africa. This finding might not be indicative of a true difference in burden across regions because of the compositional bias of the relatively sparse input data for the osteoarthritis models, with the majority of data coming from high-income locations.

Differences in the burden of osteoarthritis across regions might be caused by genetic, metabolic, and behavioural factors. Although there is little quantitative evidence to support a particular physiological causal mechanism, the literature suggests differences in prevalence of osteoarthritis in the thumb base and knee joints could be partially explained by geographical variation in occupational distribution, high BMI, behaviours such as frequency of kneeling or squatting, joint anatomy, or genetic predisposition.^19^, ^38^, ^39^, ^40^ For example, sitting in a squatting position might explain a higher prevalence of knee osteoarthritis in a cohort of Japanese females compared to US females, even though the US cohort had a higher average BMI.^41^ Furthermore, although the relative contributions of metabolic factors to the risk and severity of osteoarthritis are unknown, evidence indicates that metabolic factors associated with obesity, such as adipokine concentrations and insulin resistance, are a strong predictor of knee osteoarthritis irrespective of BMI.^42^ These findings suggest the potential for novel biotherapeutics to reduce symptoms and severity of osteoarthritis, although more research is needed.

In this study, we only address the non-fatal burden of osteoarthritis; a growing body of evidence suggests an association between osteoarthritis and excess mortality.^43^, ^44^, ^45^ Future research should further examine this link and address potential confounding caused by shared risk factors between osteoarthritis and other diseases with associated fatality.

There are several important limitations in modelling osteoarthritis globally. First, we needed to adjust data that used non-reference case-ascertainment methods. As few input data sources provide data on our reference case definition of osteoarthritis requiring radiography in addition to pain, more than 80% of all sources, including approximately 70% of non-clinical sources, required adjustment to the reference definition. In particular, ICD-coded insurance-claims data systematically undercounted osteoarthritis cases, in agreement with previously reported analyses of claims data.^29^ Second, most surveys reporting on osteoarthritis prevalence were not nationally representative but often conducted purposively in a particular subpopulation for logistical reasons. As important risks for osteoarthritis might vary between people living in cities, suburban areas or rural areas, such subnational studies might not reflect the whole country for which we are making estimates. Third, we used the same severity distributions and disability weights for all osteoarthritis sites. Given that osteoarthritis symptoms can be lessened with physical therapy, physical activity, appropriate pain medication, or joint replacement surgery, a potential update would be to grade severity by access to care by location and over time, on the basis of coverage rates for the main types of intervention. Fourth, in the calculation of total osteoarthritis we assumed independence because of insufficient data on site overlap. More population-based data are needed to quantify the occurrence of several sites of osteoarthritis observed in the same individual in clinical practice. Fifth, although SDI was included as a predictor in forecasts for osteoarthritis, future iterations should incorporate other known risk factors such as high BMI. Sixth, because US claims data constitute a large proportion of available data, these data might unduly influence global age patterns, and might be affected by care-seeking behaviour differences across the age spectrum. For example, the decreased prevalence seen in knee osteoarthritis after age 80 years might be related to decreased use of the health-care system in older age.Seventh, estimates for osteoarthritis were limited by data availability and diagnostic heterogeneity, and this limitation was particularly true for other types of osteoarthritis, which solely used US insurance-claims data. Insurance claims only captured cases of osteoarthritis that intersected with the US health system. As such, model results likely underestimated the prevalence of other osteoarthritis to a greater extent than that of hip, knee, and hand osteoarthritis. To better model other osteoarthritis, access to datasets with granular diagnostic codes in diverse geographies will be imperative. Finally, estimates of osteoarthritis were limited by the absence of reliable predictive covariates, with the exception of BMI. There is also evidence to suggest that the normal range of BMI varies in different populations,^46^ and future analyses of the attributable burden to high BMI should account for these differences.

Across all musculoskeletal disorders, GBD models rely heavily on data gathered from COPCORD studies. The ability to refine osteoarthritis estimates in the GBD going forward will depend on the availability of new input data from under-sampled geographies collected by the broader scientific community. There is currently no global initiative targeting musculoskeletal disease specifically, which means data collection using the COPCORD relies on independent funding from individual research groups. Moreover, there is increasing criticism among clinical researchers of the Kellgren-Lawrence classification system,^25^, ^26^ a component of the reference diagnostic criteria used in the present study. Criticisms include whether this system can be used to identify early osteoarthritis, varying descriptions of Kellgren-Lawrence classifications between studies, varying rater reliability between studies, and lower estimates of prevalence than other criteria such as the National Institute for Health and Care Excellence or American College of Radiology. However, it remains the most commonly used radiographical measure available across the epidemiological literature and over time. Although there is an urgent need to generate more country-level baseline measures of osteoarthritis prevalence and impact, it is unlikely that large-scale x-ray studies will be repeated given the cost and potential radiation harms. Measuring burden of osteoarthritis would be greatly enhanced by a validated self-reported measure to use in national health surveys. Groups such as the European Musculoskeletal Conditions Surveillance and Information Network and the Global Alliance for Musculoskeletal Health have begun to address this measure.^47^

Osteoarthritis is a common disorder, and case numbers have increased over the past few decades to 14·8% of the global population older than 30 years. Numbers are expected to continue to increase to the year 2050 for all sites of osteoarthritis, leading to a greater health-system burden everywhere. The main limitation to modelling the global burden of osteoarthritis was data sparsity; high-quality data collection should be prioritised and funded. Addressing burden in the long term also requires a focus on prevention and access to highly effective treatments including joint replacement, and further research into risk factors that cause osteoarthritis or increase severity and disease progression, including addressing high BMI, a known risk factor.

## Data sharing

Our study follows the Guidelines for Accurate and Transparent Health Estimates Reporting (GATHER). The findings of this study are supported by data available in public online repositories, data publicly available upon request of the data provider, and data not publicly available due to restrictions by the data provider. Non-publicly available data were used under license for the current study but may be available from the authors upon reasonable request and with permission of the data provider. Data sources used in this analysis are listed in the appendix (pp 38–51).

## Declaration of interests

BA reports an investigator-initiated trial grant from Rebecca Cooper Foundation and an investigator-initiated trial biomarkers assessment support grant from Nat Rem, and payment or honoraria for lectures, presentations, speakers bureaus, manuscript writing or educational events from Nat Rem and IRACON, all outside the submitted work. AMB reports grants or contracts paid to his institution from the Bone and Joint Decade Foundation, AO Alliance, Canadian Memorial Chiropractic College, Australian Rheumatology Association, Pan-American League of Associations for Rheumatology, World Federation of Chiropractic, and Asia Pacific League of Associations for Rheumatology, consulting fees from WHO, payment or honoraria for lectures, presentations, speakers bureaus, manuscript writing or educational events from the American College of Rheumatology, and support for attending meetings and travel from WHO, all outside the submitted work. IF and AR report payment or honoraria for lectures, presentations, speakers bureaus, manuscript writing or educational events from Avicenna Medical and Clinical Research Institute to provide critical feedback and comments on important intellectual content on Global Burden of Disease manuscripts before publication. A-FAM reports grants or contracts for MilkSafe, a novel pipeline to enrich formula milk using omics technologies, research cofinanced by the European Regional Development Fund of the EU and Greek national funds through the Operational Program Competitiveness, Entrepreneurship, and Innovation, under the call Research, Create, Innovate (project code T2EDK-02222), and from ELIDEK (Hellenic Foundation for Research and Innovation, MIMS-860), payment for expert testimony from Fondazione Cariplo, Italy for having served as an external peer reviewer; leadership, or fiduciary roles in board, society, committee, or advocacy groups, paid or unpaid with Sytematic Reviews and Annals of Epidemiology as an Editorial Board Members, and with Translational Psychiatry as an Associate Editor, stock or stock options in a family winder, and other financial or non-financial support from BGI Group for serving as a scientific officer, all outside the submitted work. JAS reports consulting fees from Crealta-Horizon, Medisys, Fidia, PK Med, Two labs, Adept Field Solutions, Clinical Care options, Clearview Healthcare Partners, Putnam associates, Focus Forward, Navigant consulting, Spherix, MedIQ, Jupiter Life Science, UBM LLC, Trio Health, Medscape, WebMD, and Practice Point Communications, and the National Institutes of Health and the American College of Rheumatology, payment or honoraria for lectures, presentations, speakers bureaus, manuscript writing, or educational events from the speaker's bureau of Simply Speaking, support for attending meetings and travel from the steering committee of OMERACT, participation on a Data Safety Monitoring Board or Advisory Board as a member of the FDA Arthritis Advisory Committee, leadership or fiduciary roles in board, society, committee, or advocacy groups, paid or unpaid as a steering committee member of the OMERACT, with the Veteran Affairs Rheumatology Field Advisory Committee as a Chair, and with the UAB Cochrane Musculoskeletal Group Satellite Center on Network Meta-analysis as an Editor, stock or stock options in Atai Life Sciences, Kintara Therapeutics, Intelligent Biosolutions, Acumen pharmaceutical, TPT Global Tech, Vaxart Pharmaceuticals, Atyu Biopharma, Adaptimmune Therapeutics, GeoVax Labs, Pieris Pharmaceuticals, Enzolytics, Seres Therapeutics, Tonix Pharmaceuticals Holding Corp., and Charlotte's Web Holdings, and previously owned stock options in Amarin, Viking, and Moderna Pharmaceuticals, all outside the submitted work. STS reports grants or contracts from the European Research Council paid to the university from the EU Horizon 2020 research innovation program (grant agreement 801790), the EU Horizon 2020 research innovation program paid to the hospital (grant agreement 945377), Region Zealand paid to the hospital as a program grant from Region Zealand (Exercise First), royalties from Munksgaard for book chapters and TrustMe-Ed for an online lecture, an honorarium from Nestlé Health Science for a webinar presentation on osteoarthritis, and other financial or non-financial interests as co-founder of GLA:D, a not-for profit initiative hosted at University of Southern Denmark aimed at implementing clinical guidelines for osteoarthritis in clinical practice, all outside the submitted work. HS reports grants or contracts paid to her institution from the Australian Government (Department of Health Grant), Medical Research Future Fund, Western Australian Government Department of Health, Bone and Joint Decade Foundation (Sweden), Curtin University (Australia), Institute for Bone and Joint Research (Australia), Canadian Memorial Chiropractic College (Canada), and support for attending meetings and travel from the Australian Pain Society, all outside the submitted work. All other authors declare no competing interests.
