# Supplementary material for: Global, regional, and national burden of osteoarthritis, 1990–2020 and projections to 2050: a systematic analysis for the Global Burden of Disease Study 2021
Source: Lancet Rheumatol. 2023 Aug 21;5(9):e508–22. doi: 10.1016/S2665-9913(23)00163-7 (PMC10477960; doi:10.1016/S2665-9913(23)00163-7)
Supplement: Supplementary appendix [file mmc1.pdf]

THE LANCET
Rheumatology

Supplementary appendix

This appendix formed part of the original submission and has been peer reviewed. We post it as supplied by the authors.

Supplement to: GBD 2021 Osteoarthritis Collaborators. Global, regional, and national burden of osteoarthritis, 1990–2020 and projections to 2050: a systematic analysis for the Global Burden of Disease Study 2021. *Lancet Rheumatol* 2023; **5:** e508–22.

Appendix to Global, regional, and national burden of osteoarthritis, 1990–2020 and projections to 2050: a systematic analysis for the Global Burden of Disease Study 2021

GBD 2021 Osteoarthritis Collaborators

Supplemental Methods

This study complies with the Guidelines for Accurate and Transparent Health Estimates Reporting (GATHER) recommendations.[1]

Global Burden of Disease location hierarchy

The below table is organized by organized by seven super-region (headers), their corresponding regions (left column), and each region's corresponding countries (right column). Countries where subnational estimates are analysed are noted.

**Central Europe, eastern Europe, and central Asia**	
Central Asia	Armenia, Azerbaijan, Georgia, Kazakhstan, Kyrgyzstan, Mongolia, Tajikistan, Turkmenistan, Uzbekistan
Central Europe	Albania, Bosnia and Herzegovina, Bulgaria, Croatia, Czech Republic, Hungary, Montenegro, North Macedonia, Poland (subnational), Romania, Serbia, Slovakia, Slovenia
Eastern Europe	Belarus, Estonia, Latvia, Lithuania, Moldova, Russia (subnational), Ukraine
**High-income**	
Australasia	Australia, New Zealand (subnational Māori + non-Māori)
High-income Asia Pacific	Brunei, Japan (subnational), Singapore, South Korea
High-income North America	Canada, Greenland, United States (subnational)
Southern Latin America	Argentina, Chile, Uruguay
Western Europe	Andorra, Austria, Belgium, Cyprus, Denmark, Finland, France, Germany, Greece, Iceland, Ireland, Israel, Italy (subnational), Luxembourg, Malta, Monaco, Netherlands, Norway (subnational), Portugal, San Marino, Spain, Sweden (subnational), Switzerland, United Kingdom (subnational)
**Latin America and Caribbean**	
Andean Latin America	Bolivia, Ecuador, Peru

Caribbean	Antigua and Barbuda, Bahamas, Barbados, Belize, Bermuda, Cuba, Dominica, Dominican Republic, Grenada, Guyana, Haiti, Jamaica, Puerto Rico, Saint Kitts and Nevis, Saint Lucia, Saint Vincent and the Grenadines, Suriname, Trinidad and Tobago, US Virgin Islands
Central Latin America	Colombia, Costa Rica, El Salvador, Guatemala, Honduras, Mexico (subnational), Nicaragua, Panama, Venezuela
Tropical Latin America	Brazil (subnational), Paraguay
**North Africa and Middle East**	
North Africa and Middle East	Afghanistan, Algeria, Bahrain, Egypt, Iran (subnational), Iraq, Jordan, Kuwait, Lebanon, Libya, Morocco, Oman, Palestine, Qatar, Saudi Arabia, Sudan, Syria, Tunisia, Türkiye, United Arab Emirates, Yemen
**South Asia**	
South Asia	Bangladesh, Bhutan, India (subnational), Nepal, Pakistan (subnational)
**Southeast Asia, east Asia, Oceania**	
East Asia	China, North Korea, Taiwan (province of China)
Oceania	American Samoa, Cook Islands, Federated States of Micronesia, Fiji, Guam, Kiribati, Marshall Islands, Nauru, Niue, Northern Mariana Islands, Palau, Papua New Guinea, Samoa, Solomon Islands, Tokelau, Tonga, Tuvalu, Vanuatu
Southeast Asia	Cambodia, Indonesia (subnational), Laos, Malaysia, Maldives, Mauritius, Myanmar, Philippines (subnational), Seychelles, Sri Lanka, Thailand, Timor-Leste, Vietnam
**Sub-Saharan Africa**	
Central sub-Saharan Africa	Angola, Central African Republic, Congo (Brazzaville), Democratic Republic of the Congo, Equatorial Guinea, Gabon
Eastern sub-Saharan Africa	Burundi, Comoros, Djibouti, Eritrea, Ethiopia (subnational), Kenya (subnational), Madagascar, Malawi, Mozambique, Rwanda, Somalia, South Sudan, Tanzania, Uganda, Zambia
Southern sub-Saharan Africa	Botswana, eSwatini, Lesotho, Namibia, South Africa (subnational), Zimbabwe
Western sub-Saharan Africa	Benin, Burkina Faso, Cape Verde, Cameroon, Chad, Côte d'Ivoire, Gambia, Ghana, Guinea, Guinea-Bissau, Liberia, Mali, Mauritania, Niger, Nigeria (subnational), São Tomé and Príncipe, Senegal, Sierra Leone, Togo

Systematic review

A systematic review of literature between 1980 and 2019 was updated using the following search string in PubMed: (("osteoarthritis" AND ("epidemiology" OR "prevalence")) AND "humans") AND ("population" OR "population groups" OR ("population" AND "groups")). The titles and abstracts of records identified through Pubmed were screened for inclusion, followed by full-text article screening for eligibility. Articles previously included in knee or hip osteoarthritis modeling were re-reviewed to look for data pertaining to other sites of osteoarthritis. The PRISMA diagram below depicts the number of studies screened at each stage, followed by delineation of exclusion criteria.

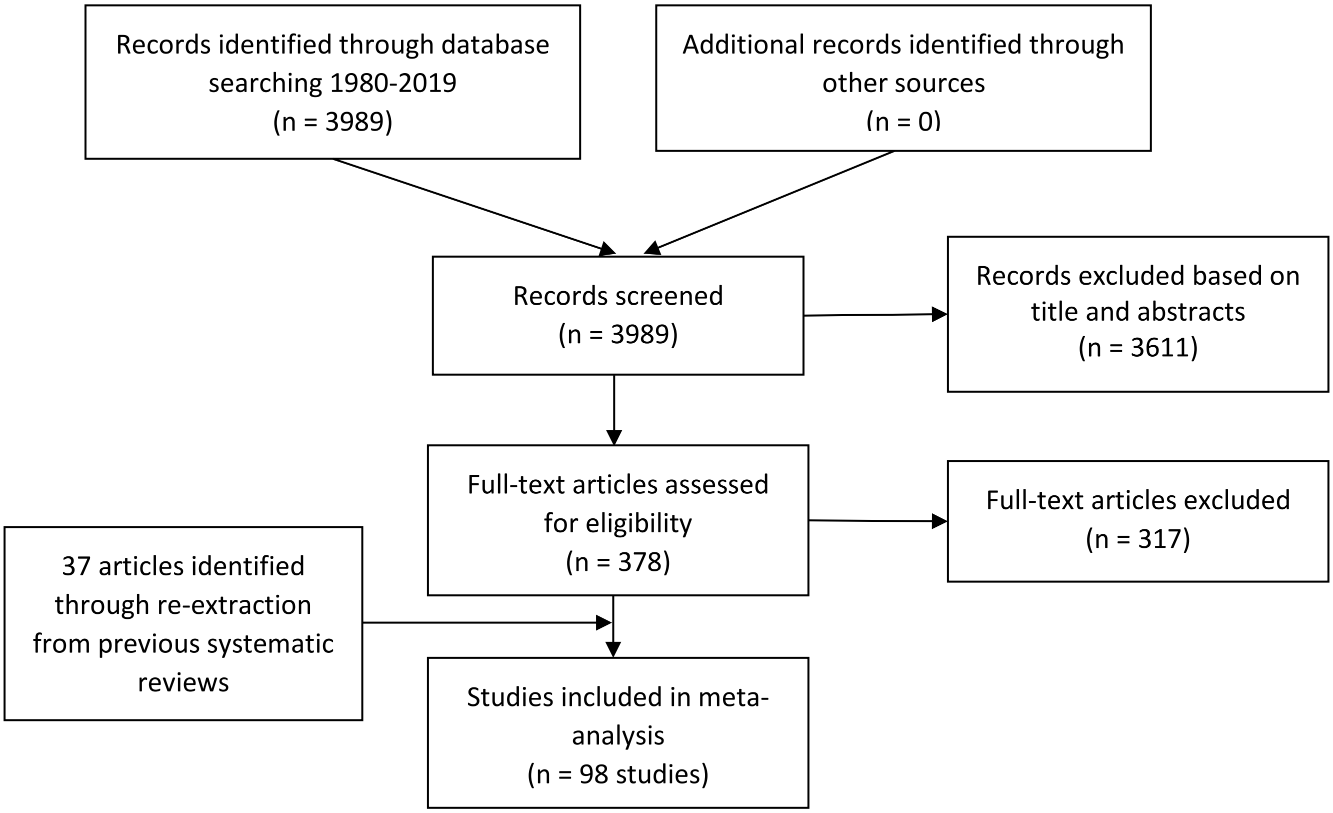

Study exclusion criteria for systematic review:

- Biased geographical selections (e.g. selecting regions within a larger geographical area that are not representative, such as a city where a targeted intervention had recently occurred that would make prevalence in the region appear artificially low, or an isolated island with significantly lower access to care than most of the geographical location it is representing)
- Data that are not representative of the location (e.g. only the lowest- or highest-income neighborhoods in a city, or only private hospitals)
- Non-GBD locations
- Sub-populations clearly not representative of the national population (eg, ethnic groups (except for NZ/Maori), specific social groups/classes, employees, etc.)
- Not a population-based study

- Low sample size (less than 150)
- Review rather than original study
- "Current or previous" osteoarthritis (which would not allow estimation of current prevalence at time of the study)
- In sources where surveys first ask whether a respondent has been diagnosed with arthritis (any type) and subsequently if that arthritis is osteoarthritis: Exclude sources for which less than 80% of the respondents to the general arthritis question specify their arthritis type.

Sex- and age-splitting methods

If studies contained both sex-specific and separate age-specific estimates, the male to female ratio was used to proportionally split age-specific data and produce age- and sex-specific prevalence data. For studies with only "both"-sex data, studies with both male and female data were used to produce male/female ratios which were log-transformed and used as input data into a regression analysis using the MR-BRT (Meta-regression – Bayesian, regularised, trimmed) tool.[2] Model results were used to proportionally split "both"-sex data. Formulas used for sex splitting are shown below:

Male prevalence:

$$prev_{male} = prev_{both} * \frac{pop_{both}}{(pop_{male} + ratio * pop_{female})}$$

Female prevalence:

$$prev_{female} = ratio * prev_{male}$$

Data with age ranges spanning more than 20 years were split into five-year age bins using global age patterns derived from GBD 2017 final models for knee and hip osteoarthritis. Age patterns from USA insurance claims data were used to split hand osteoarthritis data. Briefly, age- and sex-specific mean prevalence (or incidence) and standard error from Dismod model estimates were used to back-calculate age- and sex-specific cases and sample size with the following equations:

- Prevalence: Dismod sample size = mean prevalence * (1 – mean prevalence)/standard error^2
- Incidence: Dismod sample size = mean prevalence/standard error^2
- Prevalence and incidence: Cases = sample size * mean prevalence

Population weights were determined by dividing Dismod age pattern results for a given sex and age group by the result for the aggregated age of data that needed age splitting. Age-specific values were calculated by multiplying aggregate input data by the calculated age-specific weights.

Adjustments for non-reference case definitions (crosswalking)

Supplemental Table 1. Individual and combined joints included in modelling for hand osteoarthritis.

Grouping	Full description
Individual joints (hand)	Interphalangeal (IP); distal interphalangeal (DIP); proximal interphalangeal (PIP); carpometacarpal (CMC), ie, thumb base; metacarpophalangeal (MCP); radiocarpal (RC), ie, wrist
Multiple joints	20+ combinations, including: at least two joints; at least three joints; DIP 2-4 + at least two other joints; DIP 2-5 + IP 2-5; DIP 2-5 + PIP 2-5; IP 1 + MCP 1-5 + wrists; DIP 2-5 + PIP 2-5 + MCP 1-5 + CMC 1
Any joint	Joint(s) not specified
Generalised	Generalised osteoarthritis of the hand

Crosswalking is a standard approach in the GBD to make data as comparable as possible in spite of different case definitions or case identification protocols.

For hip and knee OA, the following case identification methods were crosswalked: X-ray reporting only, self-reported OA with pain, self-reported OA with no information on pain, or insurance claims data. Other studies identified cases of osteoarthritis through a review of medical charts, and it was assumed that these cases were diagnosed by X-ray with pain present. For hand OA, two dimensions of case definition were assessed for crosswalking: affected joint and diagnostic criteria. These alternative case definitions concerned studies reporting on the presence of OA in any single joint type (e.g. distal interphalangeal), present in multiple joint types, or diagnosed as generalized hand OA. Adjustments were also considered for studies that used X-rays, studies in which a physician diagnosed OA without X-rays, and studies that used reported pain.

For alternative case definitions, MR-BRT was used to derive adjustment factors in network analyses to compare gold standard and alternative case definition data from different studies, matched by age, sex, year and location. Model inputs were log-transformed ratios of matched data, and 10% of data were trimmed as outliers. Coefficient mean and standard error were used to adjust non-reference data, as described in the tables below.

Supplemental Table 2A. MR-BRT crosswalk adjustment factors for hip osteoarthritis.

Data input	Reference or alternative case definition	Gamma	Beta coefficient, log (95% CI)	Adjustment factor*
Radiography with pain	Ref	0.26	---	---
Radiography only	Alt		1.09 (0.89 to 1.28)	2.96 (2.44 to 3.6)

Self-reported osteoarthritis with pain	Alt		1.32 (1.15 to 1.48)	3.73 (3.16 to 4.39)
Self-reported osteoarthritis, no mention of pain	Alt		1.60 (1.18 to 2.01)	4.94 (3.26 to 7.49)
USA claims data – 2000	Alt		-2.50 (-2.96 to -2.01)	0.082 (0.052 to 0.13)
USA claims data – 2010–2016	Alt		-2.03 (-2.08 to -1.97)	0.13 (0.12 to 0.14)

**Adjustment factor is the transformed beta coefficient in normal space and can be interpreted as the factor by which the alternative case definition is adjusted to reflect what it would have been if measured as the reference. A positive beta coefficient means you will adjust down, and a negative beta coefficient means you will adjust up. Gamma is the variance of between-study effects.*

Supplemental Table 2B. MR-BRT crosswalk adjustment factors for knee osteoarthritis.

Data input	Reference or alternative case definition	Gamma	Beta coefficient, log (95% CI)	Adjustment factor*
Radiography with pain	Ref	0.38	---	---
Radiography only	Alt		0.21 (0.14 to 0.27)	1.23 (1.15 to 1.32)
Self-reported osteoarthritis with pain	Alt		0.063 (-0.027 to 0.15)	1.065 (0.97 to 1.17)
Self-reported osteoarthritis, no mention of pain	Alt		-0.77 (-0.81 to -0.72)	0.46 (0.44 to 0.48)
USA claims data – 2000	Alt		-2.26 (-2.64 to -1.88)	0.10 (0.072 to 0.15)
USA claims data – 2010–2016	Alt		-1.60 (-2.43 to -0.77)	0.20 (0.088 to 0.46)

**Adjustment factor is the transformed beta coefficient in normal space and can be interpreted as the factor by which the alternative case definition is adjusted to reflect what it would have been if measured as the reference. A positive beta coefficient means you will adjust down, and a negative beta coefficient means you will adjust up. Gamma is the variance of between-study effects.*

Supplemental Table 2C. MR-BRT crosswalk adjustment factors for hand osteoarthritis.

Data input	Reference or alternative case definition	Gamma	Beta coefficient, log (95% CI)	Adjustment factor*
Radiography with pain in a single joint type	Ref	0.36	---	---
Osteoarthritis in a single joint type	Alt		0.32 (0.29 to 0.34)	1.37 (1.34 to 1.40)
Osteoarthritis in multiple joint types	Alt		0.32 (0.30 to 0.34)	1.38 (1.35 to 1.41)
Generalised hand osteoarthritis	Alt		-0.74 (-0.80 to -0.68)	0.48 (0.45 to 0.51)
Radiography only	Alt		1.09 (1.03 to 1.15)	2.97 (2.79 to 3.16)
Physician diagnosis only	Alt		0.58 (0.51 to 0.65)	1.78 (1.66 to 1.92)
Pain only	Alt		0.055 (0.0077 to 0.10)	1.06 (1.01 to 1.11)
Radiography with pain	Alt		0.31 (0.23 to 0.39)	1.36 (1.26 to 1.48)
Physician diagnosis with pain	Alt		0.28 (0.20 to 0.35)	1.32 (1.22 to 1.42)
USA claims data – 2000	Alt		-0.48 (-0.49 to -0.47)	0.62 (0.61 to 0.62)
USA claims data – 2010–2016	Alt		-2.74 (-2.81 to -2.66)	0.065 (0.60 to 0.70)

**Adjustment factor is the transformed beta coefficient in normal space and can be interpreted as the factor by which the alternative case definition is adjusted to reflect what it would have been if measured as the reference. A positive beta coefficient means you will adjust down, and a negative beta coefficient means you will adjust up. Gamma is the variance of between-study effects.*

Disability weights

Disability weights were derived from population and internet surveys using pairwise comparisons to determine the severity of the 235 health states used in non-fatal outcomes in GBD, and population

health equivalence questions for a subset of these health states to anchor the values on a scale between zero and one. YLD estimates across all causes in the GBD underwent a final comorbidity adjustment to proportionally reduce YLDs for each component comorbid condition.[3,4] The same severity distribution and disability weights that were applied to hip and knee osteoarthritis were used to calculate YLDs for hand and other sites.

Supplemental Table 3. Severity distribution and associated disability weights.

Severity level	Lay description	DW (95% CI)
Asymptomatic		0
Mild	This person has pain in the leg, which causes some difficulty running, walking long distances, and getting up and down.	0.023 (0.013–0.037)
Moderate	This person has moderate pain in the leg, which makes the person limp, and causes some difficulty walking, standing, lifting and carrying heavy things, getting up and down, and sleeping.	0.079 (0.054–0.110)
Severe	This person has severe pain in the leg, which makes the person limp and causes a lot of difficulty walking, standing, lifting and carrying heavy things, getting up and down, and sleeping.	0.165 (0.112–0.232)

Validation experiment for forecasting method

To help validate the forecasting approach, forecasting methods were used to project prevalence from 2010-2019 using estimates from 1990-2010 as input data. Projected results were compared to known GBD results for 2010-2019, and root mean squared error (RMSE) and bias (calculated as the median value of all predicted minus observed values by age, sex, location and year) were calculated for each site of osteoarthritis, as displayed in the table below.

Supplemental Table 4. Severity distribution and associated disability weights

Site of osteoarthritis	RMSE	Bias
Hand	0.0075	0.00007
Hip	0.0005	0.00003
Knee	0.0022	0.00004
Other	0.0004	0.00005

References

1 Stevens GA, Alkema L, Black RE, *et al.* Guidelines for Accurate and Transparent Health Estimates Reporting: the GATHER statement. *The Lancet* 2016; **388**: e19–23.

2 Zheng P, Aravkin A, Barber R, Sorensen R, Murray C. Trimmed Constrained Mixed Effects Models: Formulations and Algorithms. *bioRxiv* 2020; : 2020.01.28.923599.

3 Vos T, Lim SS, Abbafati C, *et al.* Global burden of 369 diseases and injuries in 204 countries and territories, 1990–2019: a systematic analysis for the Global Burden of Disease Study 2019. *The Lancet* 2020; **396**: 1204–22.

4 Salomon JA, Haagsma JA, Davis A, *et al.* Disability weights for the Global Burden of Disease 2013 study. *Lancet Glob Health* 2015; **3**: e712–23.

Supplemental Results

Supplemental Figure 1.

Supplemental Figure 1A. Distribution of data sources for hand osteoarthritis.

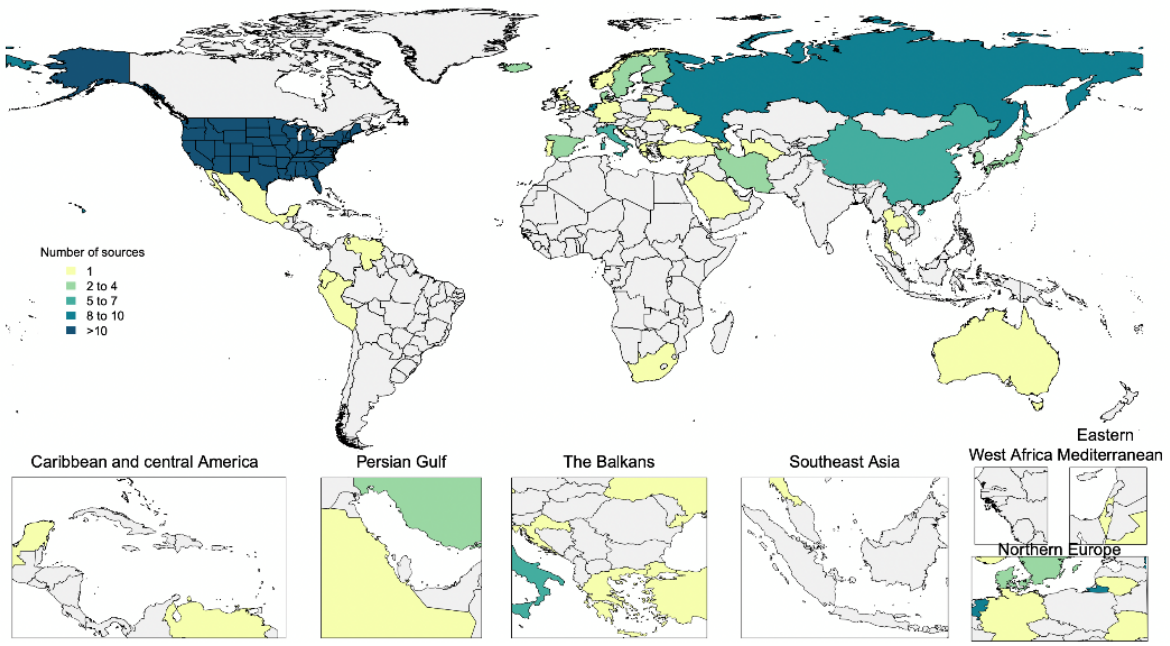

Supplemental Table 1A. Super-regional, regional, and national breakdown of locations with data sources for hand osteoarthritis.

Super-region	Region	Countries
Central Europe, Eastern Europe, and Central Asia	Central Asia	Azerbaijan, Georgia, Turkmenistan
	Central Europe	Croatia
	Eastern Europe	Lithuania, Russia, Ukraine
High-income	Australasia	Australia
	High-income Asia Pacific	Japan, Republic of Korea
	High-income North America	United States of America
	Western Europe	Andorra, Denmark, England, Finland, France, Germany, Greece, Iceland, Israel, Italy, Netherlands, Norway, Portugal, Scotland, Spain, Sweden, Wales

Latin America and Caribbean	Andean Latin America	Ecuador, Peru
	Central Latin America	Mexico, Venezuela
North Africa and Middle East	North Africa and Middle East	Iran, Saudi Arabia, Türkiye
Southeast Asia, East Asia, and Oceania	East Asia	China
	Southeast Asia	Thailand
Sub-Saharan Africa	Southern Sub-Saharan Africa	South Africa

Supplemental Table 1B. National and subnational sources in countries with subnational estimation for hand osteoarthritis.

**Country**	**Number of subnational and national sources**
China	National = 1, Subnational = 4
Iran	National = 0, Subnational = 2
Italy	National = 0, Subnational = 7
Japan	National = 2, Subnational = 2
Mexico	National = 0, Subnational = 1
Norway	National = 0, Subnational = 1
Russia	National = 0, Subnational = 8
South Africa	National = 1, Subnational = 0
Sweden	National = 0, Subnational = 2
Ukraine	National = 1, Subnational = 0
UK	National = 6, Subnational = 3
USA	National = 3, Subnational = 616**
South Africa	National = 1, Subnational = 0

*Note this table only displays countries where we produce national and subnational estimates in the Global Burden of Disease study, and does not display all countries with input data for the model, which are listed in Supplemental Table 1A; **USA claims data were tested but outliered in the OA hand model.

Supplemental Figure 1B. Distribution of data sources for hip osteoarthritis.

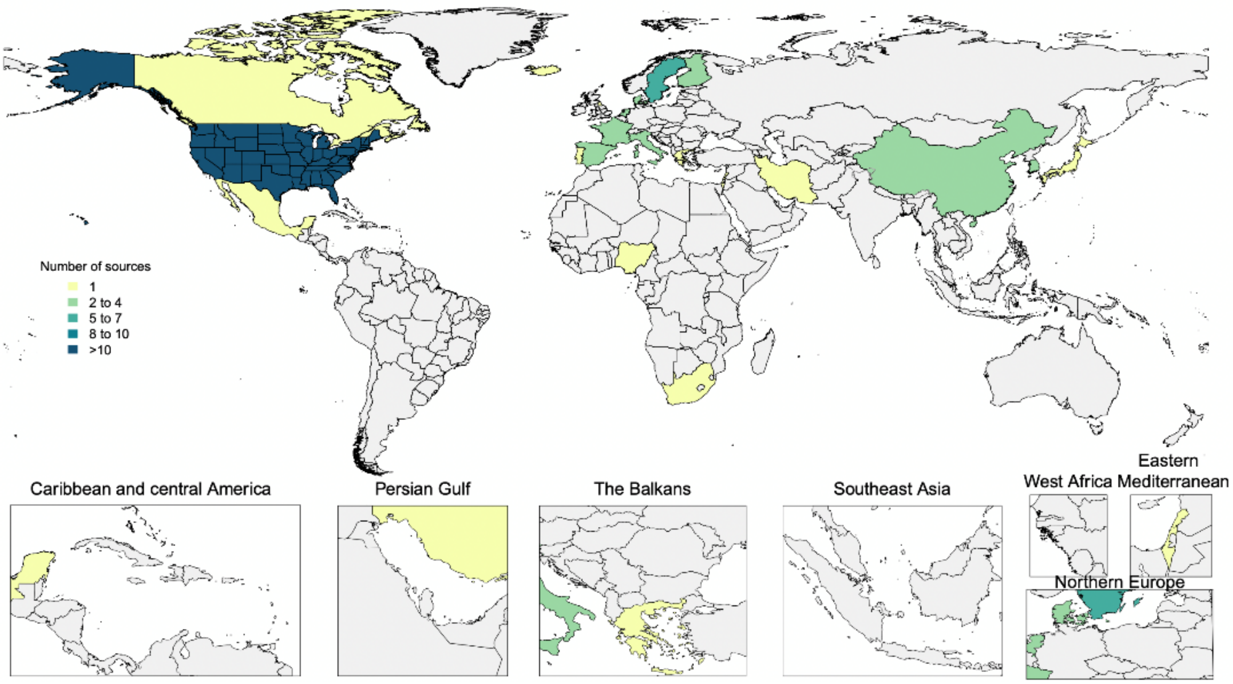

Supplemental Table 1C. Super-regional, regional, and national breakdown of locations with data sources for hip osteoarthritis.

**Super-region**	**Region**	**Countries**
High-income	High-income Asia Pacific	Japan, Republic of Korea
	High-income North America	Canada, United States of America
	Western Europe	Denmark, England, Finland, France, Greece, Iceland, Israel, Italy, Netherlands, Portugal, Spain, Sweden
Latin America and Caribbean	Central Latin America	Mexico
North Africa and Middle East	North Africa and Middle East	Iran, Lebanon

Southeast Asia, East Asia, and Oceania	East Asia	China
South Asia	South Asia	India
Sub-Saharan Africa	Southern Sub-Saharan Africa	South Africa
	Western Sub-Saharan Africa	Nigeria

Supplemental Table 1D. National and subnational sources in countries with subnational estimation for hip osteoarthritis.

**Country**	**Number of subnational and national sources**
China	National = 0, Subnational = 3
India	National = 0, Subnational = 8
Iran	National = 1, Subnational = 0
Italy	National = 0, Subnational = 2
Japan	National = 1, Subnational = 0
Mexico	National = 0, Subnational = 1
Nigeria	National = 0, Subnational = 1
South Africa	National = 0, Subnational = 1
Sweden	National = 2, Subnational = 4
UK	National = 1, Subnational = 1
USA	National = 4, Subnational = 615

*Note this table only displays countries where we produce national and subnational estimates in the Global Burden of Disease study, and does not display all countries with input data for the model, which are listed in Supplemental Table 1C.

Supplemental Figure 1C. Distribution of data sources for knee osteoarthritis.

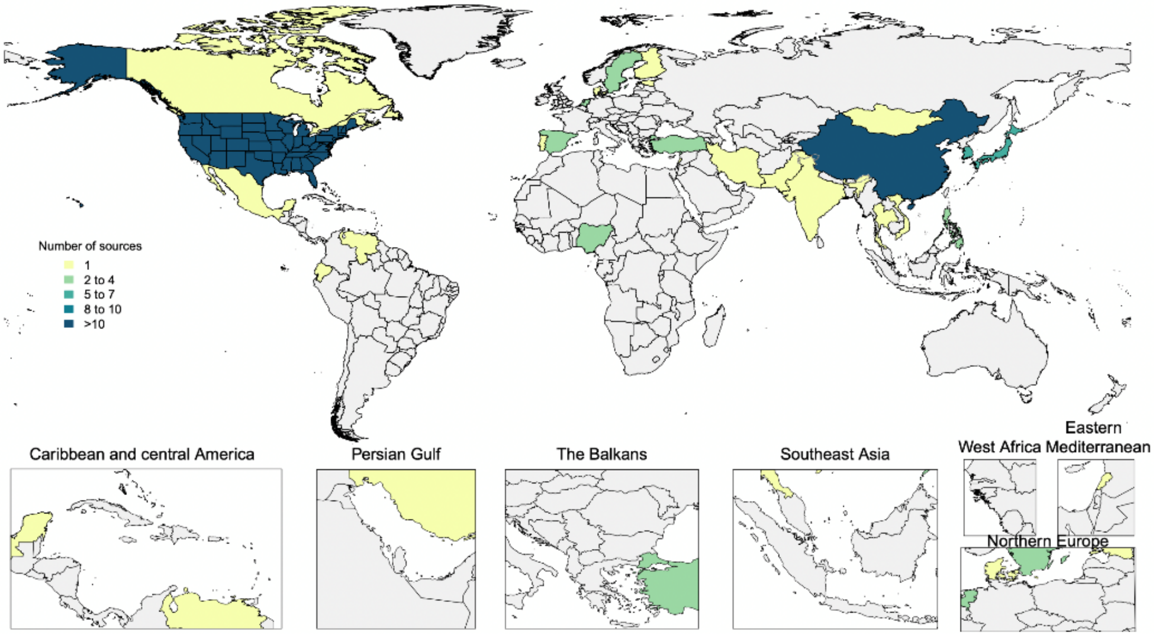

Supplemental Table 1E. Super-regional, regional, and national breakdown of locations with data sources for knee osteoarthritis.

Super-region	Region	Countries
Central Europe, Eastern Europe, and Central Asia	Central Asia	Mongolia
	Eastern Europe	Estonia
High-income	High-income Asia Pacific	Japan, Republic of Korea
	High-income North America	Canada, United States of America
	Western Europe	Denmark, England, Finland, Netherlands, Portugal, Spain, Sweden
Latin America and Caribbean	Andean Latin America	Ecuador
	Central Latin America	Mexico, Venezuela
North Africa and Middle East	North Africa and Middle East	Iran, Lebanon, Türkiye
South Asia	South Asia	Pakistan, India
	East Asia	China

Southeast Asia, East Asia, and Oceania	Southeast Asia	Philippines, Thailand, Viet Nam
Sub-Saharan Africa	Western Sub-Saharan Africa	Nigeria

Supplemental Table 1F. National and subnational sources in countries with subnational estimation for knee osteoarthritis.

**Country**	**Number of subnational and national sources**
China	National = 0, Subnational = 36
India	National = 1, Subnational = 6
Iran	National = 0, Subnational = 1
Japan	National = 2, Subnational = 5
Mexico	National = 0, Subnational = 1
Nigeria	National = 0, Subnational = 2
Pakistan	National = 0, Subnational = 1
Philippines	National = 1, Subnational = 1
Sweden	National = 0, Subnational = 2
UK	National = 4, Subnational = 0
USA	National = 6, Subnational = 615

*Note this table only displays countries where we produce national and subnational estimates in the Global Burden of Disease study, and does not display all countries with input data for the model, which are listed in Supplemental Table 1E.

Supplemental Figure 1D. Distribution of data sources for other osteoarthritis.

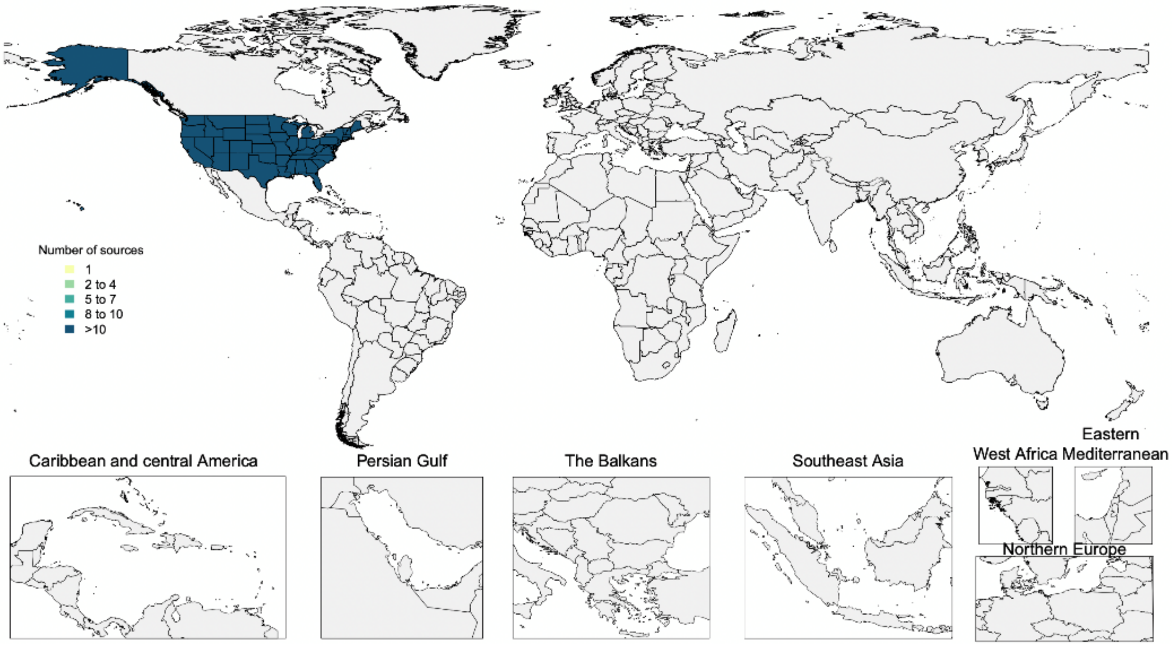

Supplemental Table 1G. Super-regional, regional, and national breakdown of locations with data sources for other osteoarthritis.

Super-region	Region	Countries
High-income	High-income North America	United States of America

Supplemental Table 1H. National and subnational sources in countries with subnational estimation for other osteoarthritis.

Country	Number of subnational and national sources
United States of America	National = 12, Subnational = 612

Supplemental Figure 2. All age and age-specific prevalence of total OA over time.

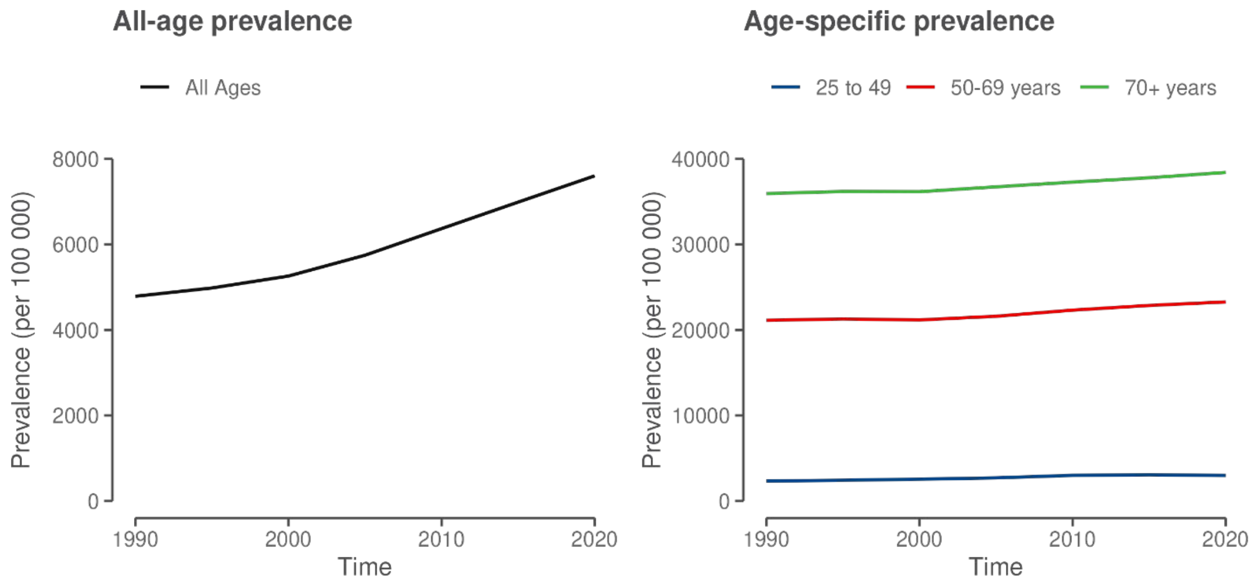

Supplemental Table 2. Number and age-standardised prevalence in 2020 for total osteoarthritis and percentage change from 1990 for each measure.

Location	Number of prevalent cases (95% UI)	% change in number of prevalent cases from 1990 to 2020 (95% UI)	Age-standardised prevalence rate per 100 000 (95% UI)	% change in age-standardised prevalence rate from 1990 to 2020 (95% UI)
Global	595 000 000 (535 000 000–656 000 000)	132.2 (130.3–134.1)	6973·6 (6287·3–7688·7)	8·7 (8·0–9·2)
Central Europe, eastern Europe, and central Asia	47 200 000 (41 600 000–53 000 000)	41.9 (40.2–43.7)	7507·0 (6642·4–8436·4)	6·8 (6·0–7·8)
Central Asia	5 660 000 (4 910 000–6 490 000)	96.0 (92.6–100.0)	7086·9 (6176·7–8077·8)	15·0 (13·1–17·1)
Central Europe	14 300 000 (12 700 000–16 000 000)	54.6 (52.4–56.8)	6979·0 (6211·8–7787·4)	10·8 (9·7–11·9)
Eastern Europe	27 200 000 (23 900 000–30 600 000)	28.8 (26.9–30.6)	7937·9 (7013·5–8931·2)	4·9 (3·7–6·0)
High income	154 000 000 (140 000 000–169 000 000)	79.8 (78.0–81.2)	7884·1 (7195·0–8648·6)	6·8 (6·3–7·4)
Australasia	3 750 000 (3 440 000–4 090 000)	125.6 (120.4–130.7)	7910·3 (7265·3–8622·6)	9·6 (7·3–11·7)
High-income Asia Pacific	34 500 000 (31 400 000–37 900 000)	107.8 (103.3–112.1)	8632·7 (7852·0–9469·1)	6·6 (5·4–7·9)

High-income North America	50 600 000 (45 900 000–56 000 000)	88.7 (87.0–90.3)	8431·7 (7676·2–9329·1)	5·2 (4·5–6·0)
Southern Latin America	6 390 000 (5 840 000–6 990 000)	96.9 (93.2–100.9)	7673·2 (7014·7–8376·8)	9·2 (7·1–11·4)
Western Europe	58 900 000 (53 800 000–64 600 000)	57.3 (55.6–59.0)	7119·1 (6512·9–7799·1)	5·3 (4·3–6·2)
Latin America and Caribbean	45 700 000 (41 100 000–50 400 000)	205.0 (201.9–208.4)	7440·9 (6695·1–8194·2)	12·1 (11·3–12·9)
Andean Latin America	4 340 000 (3 920 000–4 750 000)	213.5 (208.2–218.8)	7399·5 (6685·7–8101·2)	11·7 (10·1–13·4)
Caribbean	3 810 000 (3 470 000–4 180 000)	123.8 (120.9–127.6)	7164·3 (6537·1–7859·0)	9·6 (8·2–11·2)
Central Latin America	18 600 000 (16 700 000–20 600 000)	225.8 (221.3–231.3)	7510·8 (6746·5–8282·0)	12·4 (11·2–13·7)
Tropical Latin America	18 900 000 (17 000 000–20 900 000)	206.3 (202.4–210.1)	7438·4 (6675·0–8212·9)	12·2 (11·2–13·1)
North Africa and Middle East	29 700 000 (26 800 000–32 700 000)	213.1 (208.9–218.0)	6256·9 (5644·8–6874·9)	16·3 (14·8–17·8)
North Africa and Middle East	29 700 000 (26 800 000–32 700 000)	213.1 (208.9–218.0)	6256·9 (5644·8–6874·9)	16·3 (14·8–17·8)
South Asia	94 100 000 (84 800 000–104 000 000)	194.5 (189.1–200.8)	6319·7 (5682·1–6944·1)	16·6 (14·9–18·3)
South Asia	94 100 000 (84 800 000–104 000 000)	194.5 (189.1–200.8)	6319·7 (5682·1–6944·1)	16·6 (14·9–18·3)
Southeast Asia, east Asia, and Oceania	193 000 000 (173 000 000–214 000 000)	181.5 (174.8–188.1)	6699·3 (6029·7–7403·6)	14·3 (12·2–16·4)
East Asia	155 000 000 (140 000 000–172 000 000)	178.4 (171.5–185.7)	7019·3 (6336·0–7740·9)	13·6 (11·3–15·9)
Oceania	489 000 (441 000–541 000)	176.2 (168.9–181.4)	6208·9 (5597·6–6867·1)	9·9 (7·3–12·1)
Southeast Asia	37 500 000 (33 200 000–41 700 000)	195.5 (189.0–200.9)	5677·4 (5029·8–6318·1)	18·0 (15·6–19·8)
Sub-Saharan Africa	30 400 000 (27 400 000–33 600 000)	153.6 (150.9–156.2)	6104·3 (5470·4–6748·3)	10·2 (9·2–11·1)
Central sub-Saharan Africa	3 460 000 (3 120 000–3 820 000)	161.2 (156.3–167.3)	5946·0 (5340·7–6542·4)	5·4 (3·5–7·9)
Eastern sub-Saharan Africa	10 200 000 (9 230 000–11 300 000)	161.2 (156.4–165.1)	5821·0 (5216·8–6438·8)	13·3 (11·4–14·8)
Southern sub-Saharan Africa	4 260 000 (3 790 000–4 740 000)	133.6 (131.6–135.7)	7173·7 (6351·0–7984·1)	9·1 (8·2–10·1)
Western sub-Saharan Africa	12 500 000 (11 200 000–13 800 000)	152.9 (149.4–156.0)	6067·2 (5458·0–6707·5)	10·0 (8·8–10·9)

Note that the sum of regional cases will not exactly match global cases due to rounding.

Supplemental Table 3. Counts and age-standardised prevalence in 2020 for individual osteoarthritis sites globally, by region, and by country.

	Knee osteoarthritis		Hand osteoarthritis		Hip osteoarthritis		Other osteoarthritis	
**Location**	**Number of prevalent cases (95% UI)**	**Age-standardised prevalence rate per 100 000 (95% UI)**	**Number of prevalent cases (95% UI)**	**Age-standardised prevalence rate per 100 000 (95% UI)**	**Number of prevalent cases (95% UI)**	**Age-standardised prevalence rate per 100 000 (95% UI)**	**Number of prevalent cases (95% UI)**	**Age-standardised prevalence rate per 100 000 (95% UI)**
Global	368 000 000 (328 000 000–420 000 000)	4307·4 (3844·5–4913·3)	189 000 000 (146 000 000–238 000 000)	2226·1 (1719·7–2802·8)	35 300 000 (26 500 000–45 100 000)	417·7 (314·7–532·7)	60 900 000 (48 900 000–79 300 000)	718·4 (578·2–932·1)
Central Europe, eastern Europe, and central Asia	20 700 000 (18 400 000–23 400 000)	3279·7 (2917·5–3707·6)	24 100 000 (18 600 000–30 100 000)	3813·5 (2971·5–4786·7)	3 290 000 (2 570 000–4 210 000)	524·6 (409·3–665·1)	60 900 000 (48 900 000–79 300 000)	758·5 (604·8–992·6)
Central Asia	2 170 000 (1 890 000–2 460 000)	2722·5 (2407·8–3058·7)	3 110 000 (2 360 000–3 880 000)	3933·4 (3012·7–4886·3)	357 000 (275 000–454 000)	476·7 (374·0–608·1)	557 000 (442 000–739 000)	727·0 (582·3–952·4)
Armenia	114 000 (99 400–131 000)	2717·4 (2365·3–3098·9)	158 000 (118 000–200 000)	3700·8 (2798·1–4633·9)	19 700 (15 500–25 200)	474·2 (374·5–604·8)	30 500 (24 300–41 000)	721·8 (580·5–969·2)
Azerbaijan	293 000 (252 000–335 000)	2757·2 (2429·0–3132·1)	**412 000 (309 000–512 000)**	**3865·5 (2912·8–4787·7)**	49 500 (38 400–63 200)	491·8 (384·4–626·5)	75 400 (60 100–101 000)	732·5 (593·3–972·1)
Georgia	158 000 (140 000–179 000)	2738·2 (2442·4–3110·3)	**196 000 (148 000–246 000)**	**3330·4 (2535·7–4172·9)**	28 000 (22 000–35 900)	478·9 (375·6–614·6)	43 100 (34 500–56 700)	731·3 (587·0–970·9)
Kazakhstan	521 000 (454 000–596 000)	2847·8 (2500·8–3233·1)	833 000 (633 000–1 040 000)	4571·9 (3513·8–5711·5)	90 700 (69 400–117 000)	519·8 (403·7–662·3)	136 000 (109 000–181 000)	760·7 (614·6–1015·8)
Kyrgyzstan	127 000 (110 000–144 000)	2615·6 (2306·9–2962·6)	185 000 (141 000–232 000)	3866·4 (2970·4–4800·7)	20 100 (15 500–25 300)	437·5 (341·4–551·9)	32 600 (26 000–43 500)	701·5 (561·5–920·3)
Mongolia	68 300 (58 500–78 800)	2619·2 (2306·5–2951·6)	113 000 (85 200–139 000)	4390·3 (3346·5–5421·6)	10 700 (8 060–13 400)	437·7 (340·5–557·5)	17 200 (13 700–22 900)	699·2 (560·1–913·7)
Tajikistan	139 000 (118 000–159 000)	2446·2 (2133·6–2747·1)	168 000 (126 000–211 000)	3004·5 (2291·2–3801·8)	20 300 (15 200–25 800)	389·2 (301·8–499·8)	35 700 (28 300–46 700)	673·9 (541·1–870·1)
Turkmenistan	116 000 (99 500–132 000)	2754·5 (2417·3–3116·1)	**161 000 (121 000–202 000)**	**3843·6 (2925·9–4812·6)**	19 600 (15 000–25 200)	489·0 (378·7–632·3)	29 600 (23 500–39 600)	728·2 (583·8–961·7)
Uzbekistan	723 000 (631 000–818 000)	2694·2 (2377·1–3013·4)	1 020 000 (774 000–1 270 000)	3815·7 (2933·7–4761·2)	119 000 (90 700–150 000)	467·5 (365·8–594·6)	185 000 (147 000–245 000)	720·8 (573·5–943·9)
Central Europe	6 690 000 (5 960 000–7 610 000)	3249·8 (2892·7–3676·1)	6 610 000 (5 110 000–8 230 000)	3194·2 (2490·4–3988·5)	1 110 000 (865 000–1 410 000)	530·8 (412·3–677·8)	1 610 000 (1 280 000–2 090 000)	765·0 (612·7–1001·7)
Albania	134 000 (117 000–152 000)	3107·4 (2718·9–3534·1)	87 900 (67 000–111 000)	2040·4 (1581·0–2573·4)	20 000 (15 400–26 000)	470·3 (360·2–608·1)	31 300 (24 700–41 200)	720·1 (574·4–947·7)

Bosnia and Herzegovina	185 000 (164 000–207 000)	3148·4 (2797·3–3548·3)	169 000 (129 000–212 000)	2875·4 (2224·1–3595·8)	27 600 (21 200–36 000)	480·4 (372·4–617·3)	43 000 (34 100–56 000)	725·3 (580·8–946·0)
Bulgaria	440 000 (383 000–506 000)	3230·6 (2815·9–3682·5)	452 000 (346 000–580 000)	3319·4 (2562·9–4201·2)	69 700 (54 000–89 900)	507·8 (390·4–650·8)	105 000 (83 800–135 000)	751·9 (604·6–982·0)
Croatia	265 000 (233 000–304 000)	3234·3 (2811·5–3714·7)	**259 000 (199 000–327 000)**	**3152·0 (2451·8–3968·1)**	42 800 (33 000–55 200)	511·1 (388·7–653·1)	63 500 (50 500–82 300)	752·0 (600·0–982·4)
Czechia	649 000 (577 000–749 000)	3267·2 (2901·1–3730·2)	682 000 (528 000–859 000)	3441·5 (2677·3–4347·2)	106 000 (82 700–135 000)	524·0 (407·8–675·5)	155 000 (125 000–202 000)	762·5 (612·0–1007·0)
Hungary	603 000 (530 000–685 000)	3306·8 (2921·0–3740·1)	629 000 (487 000–786 000)	3440·4 (2685·0–4350·1)	98 400 (76 800–125 000)	528·5 (408·2–676·0)	143 000 (114 000–187 000)	764·2 (614·0–1010·0)
Montenegro	31 800 (27 800–36 100)	3240·9 (2853·6–3684·2)	32 000 (24 400–39 800)	3271·9 (2519·7–4077·0)	4 890 (3 720–6 330)	511·0 (388·3–651·2)	7 320 (5 770–9 480)	743·8 (591·8–968·4)
North Macedonia	104 000 (91 900–119 000)	3173·5 (2827·7–3624·3)	94 400 (72 300–120 000)	2897·3 (2245·4–3657·6)	15 500 (11 600–20 300)	497·1 (378·9–640·0)	23 900 (19 100–31 500)	738·2 (596·4–964·7)
Poland	2 210 000 (1 970 000–2 510 000)	3260·5 (2897·9–3696·3)	2 370 000 (1 830 000–2 950 000)	3462·9 (2688·7–4308·9)	389 000 (307 000–495 000)	565·6 (442·0–716·1)	547 000 (431 000–707 000)	788·1 (623·5–1030·7)
Romania	1 150 000 (1 020 000–1 300 000)	3239·6 (2871·1–3665·2)	956 000 (741 000–1 200 000)	2679·1 (2088·0–3365·6)	185 000 (143 000–234 000)	511·9 (392·9–652·3)	273 000 (218 000–359 000)	753·3 (602·0–1000·5)
Serbia	508 000 (446 000–584 000)	3247·4 (2826·9–3713·7)	445 000 (349 000–553 000)	2828·3 (2243·4–3519·3)	82 700 (65 300–105 000)	516·1 (402·8–662·3)	121 000 (97 800–160 000)	753·3 (610·6–1002·6)
Slovakia	299 000 (264 000–340 000)	3265·8 (2884·0–3710·5)	316 000 (246 000–400 000)	3464·3 (2706·4–4384·3)	47 200 (36 500–60 300)	519·3 (402·0–658·1)	70 200 (55 600–92 600)	760·3 (609·4–1008·0)
Slovenia	132 000 (117 000–151 000)	3265·6 (2863·2–3701·1)	135 000 (104 000–168 000)	3321·1 (2592·3–4185·2)	22 100 (17 500–27 700)	524·1 (407·3–665·9)	32 000 (25 500–42 200)	763·1 (605·5–1013·4)
Eastern Europe	11 800 000 (10 500 000–13 400 000)	3432·1 (3051·3–3882·1)	14 300 000 (11 100 000–18 000 000)	4168·2 (3262·9–5274·8)	1 820 000 (1 430 000–2 330 000)	532·2 (415·6–670·7)	2 660 000 (2 100 000–3 470 000)	761·8 (604·8–996·9)
Belarus	546 000 (486 000–618 000)	3454·4 (3072·6–3909·1)	633 000 (497 000–798 000)	4011·8 (3163·9–5036·2)	78 300 (61 400–101 000)	494·5 (385·3–626·4)	119 000 (94 700–156 000)	741·1 (596·8–978·3)
Estonia	85 600 (76 400–98 200)	3532·5 (3118·9–4029·0)	102 000 (80 400–131 000)	4250·6 (3279·6–5432·4)	13 200 (10 400–16 800)	522·5 (403·0–673·6)	19 100 (15 300–24 800)	759·3 (607·8–1000·2)
Latvia	129 000 (114 000–147 000)	3521·3 (3103·5–3996·0)	148 000 (115 000–187 000)	4083·8 (3160·1–5218·0)	19 800 (15 400–25 100)	518·6 (397·5–662·2)	28 700 (23 000–36 800)	753·7 (603·1–982·2)
Lithuania	186 000 (162 000–212 000)	3477·7 (3048·5–3974·9)	**214 000 (166 000–274 000)**	**4028·5 (3134·1–5137·2)**	27 800 (21 400–35 300)	502·7 (382·0–637·0)	41 200 (33 200–53 800)	744·8 (598·7–986·4)

Moldova	198 000 (173 000–224 000)	3450·4 (3043·0–3901·7)	181 000 (141 000–229 000)	3171·9 (2493·6–4015·8)	28 000 (21 400–36 600)	497·0 (382·6–643·7)	42 500 (33 600–55 800)	740·4 (591·9–973·3)
Russia	8 160 000 (7 250 000–9 230 000)	3450·2 (3069·8–3904·4)	**10 200 000 (7 890 000–12 800 000)**	**4308·1 (3376·0–5444·0)**	1 270 000 (1 000 000–1 630 000)	543·0 (425·4–685·0)	1 840 000 (1 450 000–2 400 000)	768·4 (609·2–1007·9)
Ukraine	2 520 000 (2 210 000–2 860 000)	3359·5 (2944·4–3804·0)	**2 890 000 (2 220 000–3 680 000)**	**3856·6 (2986·0–4862·9)**	382 000 (296 000–485 000)	511·8 (396·5–641·8)	570 000 (451 000–745 000)	748·8 (595·2–984·2)
High income	91 800 000 (81 800 000–104 000 000)	4667·2 (4174·0–5242·6)	54 300 000 (41 400 000–67 800 000)	2712·0 (2090·7–3391·1)	14 700 000 (11 300 000–18 900 000)	740·9 (565·1–955·8)	14 800 000 (12 200 000–18 600 000)	739·1 (613·4–936·6)
Australasia	2 280 000 (2 020 000–2 550 000)	4794·4 (4208·6–5404·2)	1 220 000 (925 000–1 510 000)	2522·2 (1941·4–3154·5)	367 000 (283 000–468 000)	765·8 (584·3–974·8)	373 000 (299 000–487 000)	767·2 (614·9–1001·1)
Australia	1 920 000 (1 690 000–2 160 000)	4824·9 (4214·7–5458·2)	**1 010 000 (765 000–1 250 000)**	**2499·1 (1922·5–3125·8)**	308 000 (237 000–393 000)	768·4 (584·8–979·5)	312 000 (249 000–408 000)	766·9 (613·0–996·8)
New Zealand	362 000 (320 000–405 000)	4637·8 (4109·0–5202·7)	210 000 (160 000–265 000)	2643·6 (2041·2–3348·8)	58 700 (44 800–75 700)	752·6 (569·0–957·5)	61 200 (49 000–79 700)	768·1 (618·9–1007·7)
High-income Asia Pacific	22 400 000 (20 000 000–25 600 000)	5580·6 (5028·2–6347·6)	12 600 000 (9 700 000–15 800 000)	3040·2 (2351·5–3784·0)	1 920 000 (1 480 000–2 460 000)	476·2 (358·9–609·2)	2 860 000 (2 320 000–3 670 000)	671·7 (537·3–873·9)
Brunei	20 600 (18 000–23 800)	5907·8 (5280·5–6722·5)	10 400 (7 950–13 000)	3018·9 (2317·7–3795·5)	1 750 (1 320–2 220)	502·7 (386·6–639·9)	2 260 (1 790–3 040)	680·2 (544·8–893·5)
Japan	**16 100 000 (14 400 000–18 500 000)**	**5338·0 (4804·3–6060·4)**	**9 660 000 (7 460 000–12 200 000)**	**3100·6 (2400·0–3874·7)**	**1 410 000 (1 100 000–1 800 000)**	**469·1 (354·1–598·5)**	2 190 000 (1 780 000–2 790 000)	677·4 (544·8–880·9)
South Korea	**5 840 000 (5 210 000–6 710 000)**	**6207·3 (5568·8–7101·0)**	**2 680 000 (2 030 000–3 380 000)**	**2842·5 (2178·8–3555·6)**	**461 000 (346 000–596 000)**	**496·4 (373·2–638·3)**	610 000 (478 000–797 000)	652·4 (516·4–847·3)
Singapore	492 000 (438 000–560 000)	5821·3 (5192·5–6637·0)	251 000 (197 000–316 000)	2974·6 (2332·8–3735·5)	42 600 (31 500–55 000)	506·3 (375·4–654·0)	56 100 (44 900–73 600)	671·4 (540·7–875·3)
High-income North America	28 400 000 (25 100 000–31 600 000)	4720·4 (4197·1–5304·5)	19 300 000 (14 600 000–24 100 000)	3127·3 (2391·2–3899·6)	5 490 000 (4 190 000–7 190 000)	902·8 (683·7–1167·2)	4 780 000 (4 080 000–5 730 000)	800·9 (697·9–947·0)
Canada	1 790 000 (1 580 000–2 030 000)	2734·0 (2400·8–3092·2)	1 830 000 (1 390 000–2 320 000)	2750·4 (2106·4–3496·5)	404 000 (308 000–532 000)	607·3 (459·8–800·9)	501 000 (405 000–647 000)	756·3 (609·9–975·8)
Greenland	2 170 (1 890–2 460)	2940·3 (2598·5–3310·0)	1 960 (1 480–2 510)	2632·5 (2025·3–3328·5)	457 (340–594)	629·2 (474·7–810·0)	506 (397–658)	708·9 (569·9–912·3)
USA	**26 700 000 (23 600 000–29 700 000)**	**4960·9 (4406·6–5581·9)**	**17 500 000 (13 200 000–22 000 000)**	**3174·0 (2423·8–3963·3)**	**5 100 000 (3 890 000–6 680 000)**	**938·6 (712·3–1213·1)**	**4 290 000 (3 680 000–5 100 000)**	**806·0 (705·5–946·0)**
Southern Latin America	3 850 000 (3 420 000–4 350 000)	4616·7 (4095·0–5202·4)	2 110 000 (1 640 000–2 610 000)	2519·4 (1967·0–3116·4)	573 000 (435 000–745 000)	685·4 (518·3–885·4)	612 000 (491 000–802 000)	728·0 (585·1–957·1)

Argentina	2 450 000<br>(2 180 000–2 780 000)	4568·5<br>(4054·4–5175·5)	1 390 000<br>(1 090 000–1 720 000)	2581·4<br>(2025·6–3197·8)	362 000<br>(274 000–472 000)	670·8<br>(505·2–873·0)	392 000<br>(315 000–515 000)	724·1<br>(583·2–953·7)
Chile	1 160 000<br>(1 020 000–1 310 000)	4715·6<br>(4150·9–5302·0)	594 000<br>(454 000–747 000)	2395·9<br>(1844·6–2997·5)	177 000<br>(134 000–228 000)	716·6<br>(542·6–920·2)	182 000<br>(146 000–239 000)	735·5<br>(590·8–966·2)
Uruguay	235 000<br>(208 000–267 000)	4637·1<br>(4123·1–5203·5)	127 000<br>(97 600–160 000)	2465·4<br>(1902·7–3084·2)	35 300<br>(27 200–45 900)	687·3<br>(523·4–883·1)	38 300<br>(30 900–49 700)	731·6<br>(586·4–959·8)
Western Europe	34 800 000<br>(31 300 000–39 500 000)	4172·3<br>(3720·7–4676·9)	19 100 000<br>(14 600 000–23 900 000)	2271·3<br>(1759·6–2862·9)	6 310 000<br>(4 840 000–8 010 000)	749·9<br>(570·3–957·8)	6 200 000<br>(4 990 000–8 010 000)	721·9<br>(582·7–945·3)
Andorra	6 070<br>(5 400–6 870)	4165·0<br>(3698·6–4709·7)	**3 220**<br>**(2 470–4 080)**	**2189·6**<br>**(1687·3–2770·5)**	1 100<br>(836–1 400)	761·7<br>(578·4–964·5)	1 070<br>(848–1 400)	727·0<br>(576·5–951·7)
Austria	692 000<br>(613 000–769 000)	4157·3<br>(3657·6–4612·1)	**365 000**<br>**(279 000–463 000)**	**2182·2**<br>**(1679·5–2759·5)**	124 000<br>(93 800–158 000)	735·9<br>(552·7–946·8)	122 000<br>(97 600–159 000)	714·3<br>(570·6–939·1)
Belgium	862 000<br>(767 000–976 000)	4113·0<br>(3626·9–4653·7)	**474 000**<br>**(364 000–596 000)**	**2246·2**<br>**(1742·5–2823·2)**	153 000<br>(116 000–197 000)	719·8<br>(540·4–930·8)	153 000<br>(125 000–198 000)	709·2<br>(577·3–924·9)
Cyprus	79 500<br>(70 500–90 300)	4038·2<br>(3568·4–4582·5)	**42 800**<br>**(33 200–53 900)**	**2199·3**<br>**(1716·7–2761·7)**	13 600<br>(10 400–17 400)	690·5<br>(523·2–878·5)	13 800<br>(11 000–17 800)	702·4<br>(563·9–914·4)
Denmark	**428 000**<br>**(381 000–489 000)**	**3985·9**<br>**(3509·1–4513·6)**	**235 000**<br>**(179 000–296 000)**	**2191·8**<br>**(1691·7–2768·8)**	**85 600**<br>**(65 200–110 000)**	**790·5**<br>**(597·3–1022·9)**	76 800<br>(61 400–99 700)	701·9<br>(563·4–919·1)
Finland	**465 000**<br>**(412 000–535 000)**	**4181·0**<br>**(3724·3–4750·8)**	**242 000**<br>**(183 000–308 000)**	**2175·8**<br>**(1672·7–2744·6)**	**85 200**<br>**(65 400–108 000)**	**754·6**<br>**(565·6–980·3)**	82 100<br>(65 400–106 000)	720·3<br>(577·2–941·5)
France	5 060 000<br>(4 530 000–5 740 000)	4096·3<br>(3651·2–4624·7)	2 770 000<br>(2 130 000–3 500 000)	2225·2<br>(1726·4–2807·8)	965 000<br>(735 000–1 240 000)	768·9<br>(578·6–993·4)	900 000<br>(726 000–1 150 000)	702·7<br>(564·3–914·5)
Germany	7 270 000<br>(6 540 000–8 220 000)	4193·5<br>(3780·6–4720·8)	3 890 000<br>(2 980 000–4 910 000)	2236·4<br>(1720·4–2829·3)	1 320 000<br>(1 020 000–1 650 000)	754·4<br>(575·6–950·3)	1 280 000<br>(1 030 000–1 670 000)	717·4<br>(584·4–934·4)
Greece	880 000<br>(782 000–992 000)	4178·8<br>(3734·7–4693·4)	**424 000**<br>**(325 000–539 000)**	**1999·0**<br>**(1547·5–2513·1)**	**143 000**<br>**(109 000–183 000)**	**665·5**<br>**(498·3–859·7)**	156 000<br>(129 000–201 000)	713·8<br>(580·4–935·3)
Iceland	22 700<br>(20 100–25 900)	4235·9<br>(3766·3–4791·5)	**13 000**<br>**(10 000–16 600)**	**2437·5**<br>**(1855·2–3118·6)**	**4 560**<br>**(3 440–5 820)**	**843·2**<br>**(632·6–1075·4)**	4 010<br>(3 190–5 250)	733·5<br>(588·4–970·3)
Ireland	309 000<br>(274 000–344 000)	4176·7<br>(3691·3–4675·9)	160 000<br>(123 000–202 000)	2171·1<br>(1677·9–2723·5)	55 600<br>(42 300–71 000)	749·4<br>(568·7–959·0)	53 200<br>(42 500–69 800)	713·9<br>(570·1–938·7)
Israel	473 000<br>(418 000–542 000)	4145·3<br>(3663·9–4742·7)	**257 000**<br>**(198 000–322 000)**	**2255·0**<br>**(1748·2–2834·4)**	**79 000**<br>**(60 100–98 800)**	**687·7**<br>**(516·9–866·4)**	82 200<br>(65 500–107 000)	712·2<br>(568·2–933·5)
Italy	5 230 000<br>(4 690 000–5 930 000)	4066·4<br>(3630·6–4545·6)	**3 170 000**<br>**(2 430 000–3 990 000)**	**2424·6**<br>**(1870·3–3044·6)**	**899 000**<br>**(691 000–1 150 000)**	**696·1**<br>**(524·7–897·2)**	978 000<br>(795 000–1 270 000)	731·4<br>(589·9–958·6)

Luxembourg	41 100<br>(35 900–46 300)	4143·6<br>(3606·1–4694·6)	22 300<br>(17 100–27 600)	2233·9<br>(1710·7–2769·2)	7 390<br>(5 650–9 400)	742·6<br>(564·5–947·4)	7 320<br>(5 880–9 610)	727·5<br>(583·3–957·3)
Malta	36 100<br>(32 300–40 900)	4145·9<br>(3689·6–4700·7)	19 500<br>(14 900–24 500)	2246·4<br>(1729·0–2823·0)	6 540<br>(4 970–8 230)	741·7<br>(560·0–936·6)	6 400<br>(5 080–8 300)	723·3<br>(579·5–953·9)
Monaco	3 690<br>(3 280–4 210)	4304·1<br>(3821·3–4850·7)	1 910<br>(1 460–2 400)	2221·8<br>(1719·2–2785·5)	696<br>(530–890)	801·1<br>(603·9–1034·5)	645<br>(516–838)	731·2<br>(584·8–964·9)
Netherlands	**1 390 000**<br>**(1 230 000–1 560 000)**	**4323·2**<br>**(3817·4–4830·4)**	**715 000**<br>**(543 000–896 000)**	**2216·2**<br>**(1704·0–2794·5)**	**241 000**<br>**(187 000–309 000)**	**741·0**<br>**(571·5–958·3)**	232 000<br>(185 000–303 000)	708·9<br>(566·6–928·5)
Norway	366 000<br>(328 000–413 000)	4014·0<br>(3593·7–4493·4)	**235 000**<br>**(180 000–293 000)**	**2565·8**<br>**(1987·8–3199·3)**	67 500<br>(51 700–86 200)	739·4<br>(563·7–948·4)	68 300<br>(54 700–88 300)	732·8<br>(587·7–958·3)
Portugal	**913 000**<br>**(801 000–1 030 000)**	**4173·9**<br>**(3666·6–4692·8)**	**466 000**<br>**(356 000–584 000)**	**2125·6**<br>**(1640·0–2676·9)**	**156 000**<br>**(118 000–199 000)**	**702·7**<br>**(529·0–907·4)**	159 000<br>(128 000–204 000)	707·6<br>(568·2–911·7)
San Marino	2 510<br>(2 230–2 820)	4219·8<br>(3711·2–4778·3)	1 330<br>(1 030–1 660)	2219·3<br>(1729·6–2776·3)	460<br>(354–587)	766·7<br>(583·6–983·6)	443<br>(358–575)	724·7<br>(580·7–947·0)
Spain	**3 690 000**<br>**(3 250 000–4 230 000)**	**4188·3**<br>**(3653·7–4769·3)**	**1 930 000**<br>**(1 480 000–2 430 000)**	**2178·8**<br>**(1673·9–2749·2)**	**695 000**<br>**(536 000–871 000)**	**780·9**<br>**(595·2–987·1)**	656 000<br>(525 000–843 000)	721·8<br>(569·4–942·7)
Sweden	**629 000**<br>**(542 000–733 000)**	**3311·2**<br>**(2858·0–3802·2)**	**400 000**<br>**(285 000–527 000)**	**2074·1**<br>**(1483·7–2721·9)**	**136 000**<br>**(104 000–175 000)**	**715·7**<br>**(537·9–932·5)**	144 000<br>(117 000–186 000)	737·6<br>(596·7–971·7)
Switzerland	654 000<br>(580 000–741 000)	4035·3<br>(3579·3–4544·7)	358 000<br>(274 000–451 000)	2191·8<br>(1703·6–2751·6)	113 000<br>(85 700–145 000)	686·7<br>(517·5–887·9)	116 000<br>(93 600–149 000)	694·2<br>(560·9–901·1)
UK	**5 240 000**<br>**(4 670 000–5 930 000)**	**4481·4**<br>**(3998·0–5023·0)**	**2 860 000**<br>**(2 180 000–3 570 000)**	**2425·0**<br>**(1870·6–3037·1)**	**951 000**<br>**(727 000–1 210 000)**	**809·3**<br>**(614·6–1038·9)**	902 000<br>(724 000–1 160 000)	753·8<br>(605·3–987·1)
Latin America and Caribbean	27 700 000<br>(24 600 000–31 300 000)	4500·5<br>(4019·8–5082·5)	16 000 000<br>(12 500 000–20 100 000)	2614·6<br>(2040·9–3275·8)	2 240 000<br>(1 700 000–2 820 000)	367·7<br>(279·3–464·4)	4 560 000<br>(3 630 000–5 990 000)	750·4<br>(598·4–980·4)
Andean Latin America	2 710 000<br>(2 370 000–3 060 000)	4611·8<br>(4056·5–5199·7)	1 430 000<br>(1 120 000–1 810 000)	2456·1<br>(1913·3–3080·7)	213 000<br>(163 000–270 000)	365·5<br>(278·9–465·1)	425 000<br>(341 000–557 000)	734·3<br>(587·0–957·9)
Bolivia	421 000<br>(373 000–476 000)	4487·0<br>(3994·0–5071·0)	204 000<br>(158 000–260 000)	2214·1<br>(1709·2–2812·2)	31 300<br>(23 300–39 700)	341·4<br>(255·4–434·5)	64 700<br>(51 200–86 400)	712·8<br>(568·0–953·2)
Ecuador	**769 000**<br>**(672 000–879 000)**	**4775·3**<br>**(4186·2–5443·1)**	**386 000**<br>**(300 000–486 000)**	**2413·0**<br>**(1874·9–3038·1)**	63 200<br>(48 100–80 200)	395·1<br>(301·9–502·6)	121 000<br>(96 400–162 000)	760·2<br>(605·4–1013·5)
Peru	1 530 000<br>(1 340 000–1 740 000)	4569·3<br>(3991·7–5203·8)	**850 000**<br>**(666 000–1 070 000)**	**2546·9**<br>**(1990·7–3191·7)**	120 000<br>(92 000–151 000)	357·8<br>(274·3–452·9)	242 000<br>(195 000–317 000)	727·8<br>(582·7–952·6)
Caribbean	2 380 000<br>(2 130 000–2 690 000)	4474·6<br>(4011·5–5046·3)	1 230 000<br>(961 000–1 560 000)	2314·1<br>(1808·4–2917·3)	192 000<br>(145 000–244 000)	361·6<br>(273·8–459·8)	391 000<br>(313 000–512 000)	733·5<br>(587·7–958·8)

Antigua and Barbuda	4 790 (4 220–5 450)	4447·5 (3958·0–5035·2)	2 900 (2 270–3 690)	2716·4 (2131·0–3439·9)	369 (278–470)	353·8 (267·8–450·7)	769 (614–1 020)	732·5 (589·5–966·0)
The Bahamas	19 500 (17 000–22 100)	4614·6 (4076·8–5192·4)	11 400 (8 890–14 500)	2730·6 (2137·7–3443·5)	1 550 (1 170–1 950)	378·6 (288·5–478·5)	3 070 (2 440–4 070)	748·2 (600·3–985·7)
Barbados	22 900 (20 200–25 800)	4624·8 (4084·1–5218·1)	13 500 (10 300–17 000)	2708·6 (2099·5–3431·9)	1 880 (1 400–2 440)	383·8 (286·5–492·6)	3 760 (3 000–4 950)	749·9 (604·5–991·6)
Belize	14 000 (12 100–16 200)	4636·1 (4104·6–5291·7)	6 940 (5 430–8 740)	2323·4 (1810·9–2914·4)	1 180 (883–1 480)	398·5 (301·7–500·7)	2 250 (1 800–2 930)	771·3 (621·1–995·4)
Bermuda	6 040 (5 360–6 880)	4806·6 (4251·8–5457·6)	3 510 (2 690–4 410)	2753·0 (2148·1–3460·5)	529 (399–674)	418·8 (315·2–535·8)	1 010 (809–1 320)	781·7 (629·1–1027·0)
Cuba	857 000 (763 000–975 000)	4496·1 (4012·9–5086·6)	438 000 (338 000–553 000)	2280·3 (1769·4–2867·1)	68 700 (51 400–88 200)	361·7 (270·5–459·8)	141 000 (113 000–185 000)	726·9 (584·6–956·9)
Dominica	4 060 (3 580–4 600)	4486·5 (3985·6–5059·4)	2 070 (1 590–2 620)	2275·8 (1759·9–2857·5)	329 (249–420)	366·1 (276·7–461·5)	666 (529–885)	729·2 (581·3–967·2)
Dominican Republic	432 000 (378 000–492 000)	4461·2 (3930·1–5068·1)	238 000 (186 000–300 000)	2474·1 (1926·9–3109·6)	34 600 (26 700–43 200)	361·2 (278·8–452·8)	70 500 (57 000–92 900)	741·6 (597·2–976·0)
Grenada	5 210 (4 590–5 930)	4398·7 (3906·3–4967·8)	2 860 (2 190–3 580)	2442·4 (1886·4–3040·1)	396 (297–506)	346·4 (261·4–439·5)	830 (663–1 090)	717·1 (575·4–938·0)
Guyana	29 500 (25 800–33 600)	4409·0 (3906·2–4980·1)	15 800 (12 300–19 700)	2390·6 (1867·8–2991·7)	2 220 (1 640–2 820)	345·6 (258·4–436·6)	4 630 (3 700–6 110)	719·8 (583·3–946·3)
Haiti	297 000 (262 000–340 000)	3937·7 (3533·6–4439·2)	121 000 (93 800–153 000)	1660·9 (1266·1–2106·2)	20 000 (15 100–25 500)	271·5 (207·5–345·1)	46 800 (37 700–61 900)	660·6 (532·1–861·6)
Jamaica	136 000 (121 000–154 000)	4481·9 (3975·5–5075·5)	70 400 (54 900–88 600)	2299·7 (1794·0–2896·8)	11 100 (8 490–14 000)	361·4 (274·9–458·8)	22 300 (17 900–29 500)	729·5 (584·9–963·7)
Puerto Rico	326 000 (288 000–370 000)	4973·3 (4344·2–5622·3)	182 000 (143 000–227 000)	2725·9 (2160·4–3443·1)	30 400 (23 200–39 000)	451·1 (340·9–576·4)	56 100 (44 500–73 600)	816·8 (648·8–1076·9)
Saint Kitts and Nevis	3 380 (2 900–3 860)	4612·2 (4041·0–5215·4)	1 890 (1 470–2 410)	2614·5 (2042·5–3284·9)	271 (205–346)	387·9 (296·9–494·3)	540 (424–714)	764·8 (609·5–995·1)
Saint Lucia	10 200 (8 950–11 700)	4507·6 (3977·3–5134·4)	5 420 (4 200–6 960)	2403·1 (1868·3–3081·8)	810 (613–1 030)	364·4 (277·1–462·2)	1 640 (1 330–2 180)	732·5 (596·4–970·9)
Saint Vincent and the Grenadines	6 270 (5 530–7 140)	4429·5 (3912·2–5041·7)	3 230 (2 490–4 110)	2293·3 (1775·0–2912·2)	495 (368–635)	358·8 (268·5–457·0)	1 020 (816–1 350)	729·0 (587·4–958·1)
Suriname	28 900 (25 200–33 100)	4536·3 (3996·0–5152·2)	16 900 (13 100–21 500)	2666·4 (2075·3–3365·0)	2 300 (1 760–2 950)	369·5 (283·8–473·3)	4 660 (3 720–6 180)	743·6 (597·4–981·7)

Trinidad and Tobago	88 800 (77 900–100 000)	4615·5 (4063·7–5218·7)	49 500 (38 600–62 400)	2572·9 (2027·5–3239·1)	7 290 (5 440–9 390)	384·3 (288·2–490·7)	14 400 (11 400–19 100)	749·0 (598·9–991·3)
Virgin Islands	9 000 (7 920–10 200)	4901·6 (4280·8–5569·1)	4 900 (3 750–6 170)	2670·2 (2086·0–3375·5)	781 (573–1 020)	433·5 (320·9–554·3)	1 470 (1 170–1 930)	791·5 (635·0–1042·8)
Central Latin America	11 200 000 (9 930 000–12 700 000)	4506·6 (4019·5–5094·3)	6 690 000 (5 230 000–8 410 000)	2706·1 (2112·8–3394·5)	856 000 (648 000–1 070 000)	347·7 (263·4–437·9)	1 840 000 (1 460 000–2 410 000)	748·9 (597·0–977·5)
Colombia	2 430 000 (2 170 000–2 760 000)	4451·6 (3961·1–5041·0)	1 370 000 (1 080 000–1 750 000)	2497·8 (1964·1–3169·5)	175 000 (132 000–221 000)	319·1 (240·3–402·4)	399 000 (320 000–524 000)	724·4 (582·3–950·4)
Costa Rica	245 000 (216 000–276 000)	4559·9 (4019·9–5143·9)	131 000 (103 000–166 000)	2438·1 (1921·3–3066·2)	18 000 (13 600–22 900)	336·6 (254·0–426·4)	39 600 (32 000–52 400)	739·3 (598·2–975·7)
El Salvador	272 000 (237 000–308 000)	4554·3 (3968·6–5147·6)	144 000 (113 000–180 000)	2392·6 (1879·2–3004·2)	19 800 (15 100–25 000)	326·0 (246·8–413·1)	43 900 (35 600–57 700)	726·9 (587·3–957·2)
Guatemala	461 000 (404 000–528 000)	4243·4 (3748·8–4833·5)	215 000 (168 000–272 000)	2008·0 (1560·5–2551·5)	31 100 (23 700–39 300)	289·3 (221·1–366·8)	73 600 (58 600–95 700)	696·4 (556·2–898·8)
Honduras	280 000 (247 000–317 000)	4325·9 (3843·4–4853·6)	130 000 (102 000–165 000)	2042·4 (1604·0–2606·0)	19 300 (14 500–24 100)	302·5 (230·6–382·3)	44 300 (35 500–58 000)	711·1 (574·3–934·7)
Mexico	**5 660 000 (5 010 000–6 450 000)**	**4570·5 (4074·5–5177·2)**	**3 700 000 (2 890 000–4 620 000)**	**3011·2 (2346·8–3744·1)**	**461 000 (348 000–576 000)**	**376·7 (285·7–473·4)**	941 000 (744 000–1 240 000)	773·4 (612·3–1012·9)
Nicaragua	211 000 (185 000–241 000)	4411·5 (3907·3–5034·1)	98 300 (77 100–124 000)	2090·3 (1626·2–2656·4)	14 700 (11 000–18 400)	311·7 (233·5–392·9)	32 900 (26 400–44 100)	716·1 (575·2–957·0)
Panama	189 000 (166 000–214 000)	4391·1 (3846·0–4954·4)	104 000 (80 600–130 000)	2403·6 (1865·7–3026·7)	13 700 (10 200–17 200)	316·8 (236·1–398·0)	31 300 (25 300–40 800)	726·4 (585·0–945·3)
Venezuela	**1 390 000 (1 230 000–1 570 000)**	**4494·9 (4008·5–5098·3)**	**768 000 (590 000–980 000)**	**2492·8 (1915·4–3173·7)**	100 000 (76 100–127 000)	329·9 (250·9–416·7)	225 000 (181 000–297 000)	738·0 (596·9–972·4)
Tropical Latin America	11 400 000 (10 200 000–12 900 000)	4473·2 (4000·7–5056·7)	6 650 000 (5 170 000–8 330 000)	2625·1 (2044·2–3283·4)	980 000 (743 000–1 240 000)	388·7 (295·8–490·6)	1 910 000 (1 510 000–2 520 000)	758·8 (602·9–995·7)
Brazil	11 100 000 (9 940 000–12 600 000)	4474·7 (4002·4–5056·8)	6 510 000 (5 070 000–8 170 000)	2633·2 (2050·2–3293·4)	960 000 (728 000–1 210 000)	389·7 (296·5–491·8)	1 870 000 (1 480 000–2 470 000)	759·7 (603·5–996·8)
Paraguay	257 000 (224 000–294 000)	4402·3 (3860·2–5048·1)	132 000 (102 000–166 000)	2273·0 (1756·4–2847·7)	20 200 (15 200–25 300)	347·1 (263·0–439·3)	41 200 (32 800–54 800)	718·3 (574·4–949·4)
North Africa and Middle East	18 200 000 (15 900 000–20 800 000)	3818·5 (3391·9–4347·8)	8 820 000 (6 880 000–11 000 000)	1893·3 (1471·8–2392·0)	1 390 000 (1 040 000–1 730 000)	296·5 (224·5–368·6)	3 540 000 (2 840 000–4 680 000)	781·5 (625·9–1025·9)
North Africa and Middle East	18 200 000 (15 900 000–20 800 000)	3818·5 (3391·9–4347·8)	8 820 000 (6 880 000–11 000 000)	1893·3 (1471·8–2392·0)	1 390 000 (1 040 000–1 730 000)	296·5 (224·5–368·6)	3 540 000 (2 840 000–4 680 000)	781·5 (625·9–1025·9)

Afghanistan	486 000 (420 000–561 000)	3404·4 (3016·2–3883·1)	144 000 (108 000–182 000)	1075·6 (819·6–1376·2)	31 800 (23 000–39 800)	225·9 (171·2–286·5)	91 500 (72 800–122 000)	702·8 (563·7–926·7)
Algeria	1 400 000 (1 220 000–1 610 000)	3821·0 (3346·1–4366·0)	755 000 (588 000–941 000)	2077·4 (1622·9–2609·0)	106 000 (78 000–132 000)	295·3 (222·2–368·7)	278 000 (223 000–371 000)	790·7 (633·6–1058·7)
Bahrain	56 400 (48 200–64 700)	3974·5 (3536·1–4562·7)	29 200 (22 100–36 900)	2149·6 (1672·2–2729·8)	4 670 (3 320–6 060)	338·2 (255·1–426·9)	11 000 (8 630–14 900)	845·4 (673·2–1114·0)
Egypt	2 720 000 (2 350 000–3 140 000)	3894·1 (3447·5–4461·5)	1 150 000 (886 000–1 440 000)	1670·0 (1287·0–2103·8)	209 000 (154 000–262 000)	311·1 (234·0–386·4)	527 000 (418 000–705 000)	801·1 (639·9–1060·1)
Iran	**2 860 000 (2 510 000–3 270 000)**	**3611·9 (3216·1–4099·2)**	**1 650 000 (1 290 000–2 080 000)**	**2125·8 (1648·8–2687·0)**	**237 000 (177 000–294 000)**	**298·7 (225·7–371·2)**	581 000 (465 000–771 000)	763·8 (610·8–1000·9)
Iraq	975 000 (837 000–1 120 000)	3812·1 (3319·9–4333·9)	463 000 (365 000–588 000)	1859·4 (1455·8–2365·5)	72 700 (54 100–90 600)	288·7 (219·9–363·2)	188 000 (150 000–251 000)	778·4 (622·9–1038·3)
Jordan	312 000 (266 000–361 000)	3969·8 (3503·0–4571·2)	168 000 (129 000–213 000)	2183·2 (1703·6–2777·4)	24 600 (18 100–31 600)	322·2 (243·1–402·7)	59 800 (47 400–80 300)	813·6 (646·7–1087·7)
Kuwait	143 000 (119 000–165 000)	4116·5 (3617·4–4750·5)	74 600 (55 900–94 400)	2179·3 (1695·4–2770·8)	12 600 (9 120–16 000)	358·7 (273·6–448·4)	27 300 (21 500–36 600)	865·2 (695·7–1150·4)
Lebanon	**204 000 (181 000–232 000)**	**3852·0 (3408·6–4384·0)**	119 000 (93 200–150 000)	2258·8 (1759·0–2851·5)	**16 000 (12 000–19 900)**	**300·7 (225·5–373·5)**	43 000 (34 100–57 400)	813·7 (643·1–1087·7)
Libya	225 000 (193 000–261 000)	3897·2 (3398·3–4462·5)	122 000 (94 200–155 000)	2142·9 (1664·8–2733·0)	17 600 (13 100–22 000)	305·6 (230·4–380·6)	43 500 (34 900–58 600)	794·2 (635·3–1054·3)
Morocco	1 260 000 (1 110 000–1 440 000)	3722·6 (3300·2–4225·7)	596 000 (464 000–744 000)	1793·1 (1393·2–2245·8)	89 900 (66 400–115 000)	275·6 (206·9–349·8)	246 000 (196 000–331 000)	757·9 (610·5–1007·3)
Oman	90 900 (78 300–105 000)	3899·0 (3458·2–4461·9)	49 200 (36 800–64 100)	2200·0 (1719·1–2813·3)	7 840 (5 720–9 850)	317·0 (238·6–395·6)	17 300 (13 600–23 300)	813·0 (648·7–1082·9)
Palestine	98 400 (85 900–114 000)	3686·2 (3261·5–4184·3)	53 800 (41 400–68 100)	2071·8 (1600·9–2572·1)	7 020 (5 180–8 790)	268·4 (202·5–335·0)	18 800 (15 100–25 000)	749·9 (596·4–981·5)
Qatar	65 900 (55 400–76 600)	4052·1 (3601·6–4612·8)	31 000 (23 300–41 200)	1969·8 (1551·7–2500·6)	6 510 (4 610–8 300)	378·9 (279·8–475·3)	13 100 (10 000–18 000)	902·1 (711·9–1199·2)
Saudi Arabia	938 000 (774 000–1 080 000)	4035·6 (3524·5–4617·6)	**488 000 (363 000–628 000)**	**2151·4 (1682·7–2711·2)**	81 700 (57 900–107 000)	346·6 (259·2–437·6)	177 000 (139 000–239 000)	852·0 (679·3–1121·3)
Sudan	733 000 (638 000–840 000)	3599·0 (3171·3–4096·6)	300 000 (230 000–377 000)	1505·5 (1154·9–1915·1)	53 200 (39 300–68 400)	261·9 (196·8–327·5)	144 000 (116 000–193 000)	749·6 (605·1–996·1)
Syria	514 000 (454 000–583 000)	3744·2 (3353·7–4206·5)	259 000 (202 000–331 000)	1910·8 (1489·4–2417·9)	36 100 (26 700–45 200)	280·9 (212·0–348·1)	99 200 (78 600–131 000)	762·6 (609·0–994·7)

Tunisia	507 000<br>(443 000–580 000)	3776·8<br>(3314·0–4310·7)	283 000<br>(221 000–361 000)	2126·8<br>(1666·8–2708·6)	36 900<br>(27 900–46 500)	282·1<br>(215·1–352·7)	99 600<br>(79 400–131 000)	757·5<br>(606·9–986·9)
Türkiye	**3 800 000<br>(3 310 000–4 290 000)**	**4075·4<br>(3560·8–4588·0)**	**1 740 000<br>(1 350 000–2 200 000)**	**1883·7<br>(1461·1–2377·6)**	283 000<br>(214 000–350 000)	306·7<br>(233·6–380·9)	731 000<br>(588 000–965 000)	795·3<br>(641·4–1046·1)
United Arab Emirates	276 000<br>(223 000–328 000)	3909·4<br>(3475·5–4478·2)	130 000<br>(91 500–167 000)	1893·4<br>(1451·0–2386·8)	25 400<br>(17 600–33 500)	349·2<br>(262·7–429·0)	50 900<br>(37 800–70 400)	846·5<br>(669·8–1119·3)
Yemen	482 000<br>(410 000–543 000)	3308·9<br>(2869·6–3720·5)	209 000<br>(160 000–264 000)	1485·2<br>(1134·6–1875·7)	31 300<br>(23 400–39 500)	214·8<br>(163·9–269·4)	93 300<br>(76 200–124 000)	687·0<br>(556·6–904·6)
South Asia	57 600 000<br>(50 600 000–66 000 000)	3835·2<br>(3397·5–4372·9)	29 800 000<br>(23 100 000–37 700 000)	2050·6<br>(1583·5–2577·5)	4 490 000<br>(3 360 000–5 700 000)	300·5<br>(225·6–377·0)	9 890 000<br>(7 830 000–13 100 000)	691·7<br>(550·7–906·4)
South Asia	57 600 000<br>(50 600 000–66 000 000)	3835·2<br>(3397·5–4372·9)	29 800 000<br>(23 100 000–37 700 000)	2050·6<br>(1583·5–2577·5)	4 490 000<br>(3 360 000–5 700 000)	300·5<br>(225·6–377·0)	9 890 000<br>(7 830 000–13 100 000)	691·7<br>(550·7–906·4)
Bangladesh	5 040 000<br>(4 370 000–5 710 000)	3592·3<br>(3135·4–4080·1)	2 380 000<br>(1 850 000–3 040 000)	1738·2<br>(1347·4–2204·7)	374 000<br>(280 000–475 000)	267·8<br>(200·9–337·0)	871 000<br>(695 000–1 140 000)	645·1<br>(516·7–840·9)
Bhutan	23 400<br>(20 500–26 500)	3893·5<br>(3446·4–4403·8)	8 660<br>(6 610–10 900)	1500·0<br>(1137·2–1904·8)	1 940<br>(1 460–2 440)	319·9<br>(242·4–399·2)	3 940<br>(3 170–5 170)	690·1<br>(556·8–900·0)
India	**47 700 000<br>(42 200 000–54 700 000)**	**3926·0<br>(3491·1–4476·5)**	24 700 000<br>(19 100 000–31 200 000)	2099·7<br>(1620·8–2637·5)	**3 640 000<br>(2 720 000–4 620 000)**	**301·5<br>(226·0–378·4)**	8 080 000<br>(6 390 000–10 700 000)	695·7<br>(554·0–912·3)
Nepal	895 000<br>(777 000–1 030 000)	3773·0<br>(3298·4–4304·1)	293 000<br>(225 000–374 000)	1285·9<br>(985·5–1642·7)	69 200<br>(51 500–88 500)	295·1<br>(220·0–373·1)	149 000<br>(120 000–196 000)	661·5<br>(533·9–859·9)
Pakistan	3 920 000<br>(3 310 000–4 550 000)	3226·2<br>(2778·5–3721·2)	2 380 000<br>(1 840 000–3 000 000)	2090·7<br>(1607·1–2617·1)	407 000<br>(304 000–517 000)	329·7<br>(249·5–410·0)	793 000<br>(624 000–1 060 000)	712·9<br>(561·5–934·6)
Southeast Asia, east Asia, and Oceania	134 000 000<br>(117 000 000–<br>153 000 000)	4599·7<br>(4056·7–5270·0)	46 900 000<br>(35 500 000–60 400 000)	1659·7<br>(1264·2–2122·4)	7 360 000<br>(5 480 000–9 380 000)	263·8<br>(198·3–333·5)	20 000 000<br>(15 800 000–26 300 000)	706·0<br>(562·6–919·2)
East Asia	111 000 000<br>(97 600 000–<br>128 000 000)	5014·7<br>(4412·6–5740·5)	34 700 000<br>(26 000 000–44 700 000)	1589·4<br>(1205·4–2037·4)	5 570 000<br>(4 140 000–7 120 000)	259·8<br>(195·1–329·0)	15 600 000<br>(12 300 000–20 400 000)	710·2<br>(565·3–925·0)
China	**108 000 000<br>(94 300 000–<br>124 000 000)**	**5013·7<br>(4410·6–5739·8)**	**33 300 000<br>(25 000 000–43 000 000)**	**1580·7<br>(1197·6–2027·0)**	**5 400 000<br>(4 010 000–6 900 000)**	**260·7<br>(195·8–330·2)**	15 100 000<br>(11 900 000–19 800 000)	711·3<br>(566·1–926·9)
North Korea	1 620 000<br>(1 410 000–1 850 000)	4771·1<br>(4213·5–5431·6)	456 000<br>(344 000–592 000)	1384·0<br>(1050·2–1777·1)	65 100<br>(47 800–82 200)	199·2<br>(147·9–250·9)	206 000<br>(165 000–270 000)	625·7<br>(501·3–814·5)
Taiwan (province of China)	2 130 000<br>(1 870 000–2 440 000)	5280·7<br>(4616·5–6043·6)	911 000<br>(705 000–1 160 000)	2219·7<br>(1725·8–2814·3)	106 000<br>(80 400–136 000)	263·7<br>(199·9–333·2)	296 000<br>(235 000–394 000)	718·0<br>(574·4–950·8)
Oceania	326 000<br>(279 000–380 000)	4037·7<br>(3541·9–4634·0)	121 000<br>(93 700–152 000)	1658·8<br>(1277·2–2094·8)	22 500<br>(16 900–28 300)	302·2<br>(229·7–384·4)	50 200<br>(40 200–66 800)	698·8<br>(563·7–923·9)

American Samoa	2 520 (2 160–2 950)	4846·5 (4208·5–5640·3)	1 260 (993–1 610)	2516·2 (1984·2–3197·8)	211 (158–270)	429·7 (325·6–547·2)	400 (315–537)	809·8 (645·8–1073·9)
Cook Islands	1 230 (1 070–1 410)	4835·9 (4201·1–5524·0)	649 (503–830)	2523·1 (1972·0–3209·2)	109 (82.5–140)	433·7 (329·1–547·5)	213 (169–279)	827·6 (659·3–1085·0)
Fiji	37 500 (32 400–43 000)	4558·0 (4018·2–5234·6)	18 500 (14 400–23 000)	2365·9 (1845·7–2951·2)	2 860 (2 110–3 640)	371·6 (276·2–471·1)	5 900 (4 690–7 970)	771·3 (618·7–1033·6)
Guam	9 290 (8 090–10 600)	4673·8 (4098·3–5324·5)	5 020 (3 880–6 430)	2540·2 (1976·3–3231·7)	783 (593–999)	402·9 (305·6–511·0)	1 570 (1 260–2 060)	797·4 (641·3–1040·8)
Kiribati	3 360 (2 840–3 890)	4393·7 (3815·7–5009·6)	1 630 (1 270–2 050)	2294·5 (1791·8–2905·1)	231 (172–292)	326·4 (247·0–414·9)	498 (396–667)	724·8 (581·3–951·5)
Marshall Islands	1 640 (1 370–1 920)	4126·6 (3534·7–4723·4)	942 (726–1 190)	2562·3 (1993·1–3217·8)	114 (84.6–144)	315·3 (239·6–401·2)	250 (197–332)	714·9 (570·2–942·9)
Federated States of Micronesia	3 510 (2 950–4 030)	4439·7 (3849·0–5045·2)	1 740 (1 340–2 170)	2353·9 (1817·9–2950·4)	251 (187–322)	347·4 (261·3–442·2)	527 (416–707)	736·9 (589·4–978·2)
Nauru	230 (187–267)	4525·3 (3932·2–5203·1)	110 (83.1–142)	2453·5 (1891·5–3111·0)	16.3 (12–21)	360·7 (269·1–458·6)	32 (25–43.6)	759·2 (604·1–1007·4)
Niue	104 (91.5–121)	4714·0 (4144·0–5440·2)	55.6 (43.3–70.1)	2517·0 (1980·0–3167·6)	8.7 (6.52–11.2)	402·2 (302·5–510·0)	17.8 (14.2–23.3)	805·1 (645·1–1052·0)
Northern Mariana Islands	2 950 (2 500–3 400)	4598·5 (3993·8–5267·3)	1 490 (1 120–1 910)	2466·3 (1915·5–3093·7)	223 (164–288)	394·3 (295·6–500·0)	455 (358–605)	775·3 (627·8–1019·4)
Palau	1 160 (981–1 340)	4677·0 (4077·7–5400·1)	583 (455–748)	2497·4 (1959·8–3182·0)	91.7 (66.7–117)	407·4 (304·3–516·7)	184 (145–248)	805·4 (649·6–1070·4)
Papua New Guinea	214 000 (181 000–251 000)	3862·9 (3373·8–4438·4)	70 000 (53 600–88 700)	1413·0 (1078·6–1791·6)	14 100 (10 600–17 800)	276·9 (211·7–353·8)	32 600 (26 100–43 400)	674·0 (544·3–887·8)
Samoa	7 040 (6 080–8 030)	4485·1 (3905·3–5108·4)	3 470 (2 670–4 380)	2301·9 (1774·9–2930·1)	554 (415–696)	370·2 (278·6–468·8)	1 140 (901–1 490)	765·5 (609·8–986·8)
Solomon Islands	14 300 (12 200–16 400)	4082·8 (3607·1–4644·7)	6 160 (4 820–7 800)	1945·3 (1494·2–2474·0)	983 (733–1 240)	306·8 (233·9–387·7)	2 140 (1 700–2 860)	702·8 (566·3–931·0)
Tokelau	63.6 (55–73.5)	4454·3 (3874·9–5140·6)	33.5 (26–42.5)	2368·9 (1852·7–2987·6)	5.13 (3.81–6.52)	370·5 (278·1–467·1)	10.8 (8.64–14.2)	772·4 (624·5–1011·0)
Tonga	3 720 (3 230–4 280)	4567·7 (3990·4–5268·6)	1 640 (1 280–2 070)	2043·7 (1586·6–2587·7)	299 (226–378)	372·8 (282·7–471·3)	607 (486–801)	761·3 (607·8–999·9)
Tuvalu	468 (404–538)	4368·9 (3802·0–5010·0)	248 (193–317)	2371·5 (1850·5–3011·6)	35.2 (26.2–44.7)	342·1 (256·6–433·3)	76.5 (60.2–101)	741·8 (590·4–969·5)

Vanuatu	7 660<br>(6 490–8 730)	4104·7<br>(3542·4–4629·5)	2 300<br>(1 760–2 950)	1308·7<br>(996·4–1672·3)	563<br>(423–720)	315·9<br>(239·4–402·0)	1 240<br>(994–1 640)	712·2<br>(574·4–934·6)
Southeast Asia	21 800 000<br>(18 900 000–25 200 000)	3256·3<br>(2874·1–3736·7)	12 100 000<br>(9 350 000–15 400 000)	1893·4<br>(1455·8–2402·8)	1 770 000<br>(1 330 000–2 240 000)	277·8<br>(209·7–352·0)	4 360 000<br>(3 460 000–5 770 000)	690·4<br>(551·9–899·3)
Cambodia	409 000<br>(350 000–470 000)	3143·2<br>(2736·8–3605·8)	169 000<br>(129 000–221 000)	1371·3<br>(1047·5–1780·1)	29 300<br>(21 700–37 900)	235·0<br>(175·5–298·4)	77 800<br>(61 900–103 000)	639·6<br>(514·3–830·2)
Indonesia	7 810 000<br>(6 730 000–9 000 000)	3206·3<br>(2819·4–3634·5)	4 590 000<br>(3 530 000–5 840 000)	2024·2<br>(1551·6–2567·9)	659 000<br>(490 000–834 000)	287·8<br>(217·0–362·9)	1 570 000<br>(1 240 000–2 080 000)	700·6<br>(558·6–915·8)
Laos	155 000<br>(133 000–180 000)	3174·8<br>(2792·1–3668·0)	64 100<br>(49 600–82 000)	1412·3<br>(1087·7–1805·8)	11 800<br>(8 800–14 900)	254·3<br>(190·6–320·9)	30 000<br>(23 900–39 400)	671·1<br>(537·4–876·2)
Malaysia	1 010 000<br>(877 000–1 170 000)	3475·2<br>(3041·2–4026·2)	646 000<br>(496 000–811 000)	2293·0<br>(1759·1–2886·7)	88 100<br>(65 300–111 000)	314·6<br>(235·5–398·6)	204 000<br>(162 000–270 000)	735·7<br>(590·8–969·4)
Maldives	12 000<br>(10 200–13 800)	3259·5<br>(2833·7–3706·3)	8 160<br>(6 340–10 300)	2394·3<br>(1829·7–3035·6)	1 050<br>(782–1 320)	289·8<br>(219·6–364·6)	2 340<br>(1 890–3 110)	707·1<br>(563·9–937·3)
Mauritius	64 900<br>(56 900–75 200)	3508·0<br>(3089·4–4061·3)	45 600<br>(35 100–57 500)	2471·6<br>(1921·7–3112·0)	5 530<br>(4 110–7 100)	308·6<br>(231·2–389·9)	13 100<br>(10 400–17 400)	717·2<br>(575·8–946·1)
Myanmar	1 630 000<br>(1 380 000–1 880 000)	3219·2<br>(2782·4–3703·6)	857 000<br>(654 000–1 080 000)	1771·1<br>(1357·5–2245·9)	120 000<br>(90 400–153 000)	249·8<br>(190·1–314·9)	313 000<br>(247 000–421 000)	657·6<br>(521·4–870·1)
Philippines	**2 510 000<br>(2 180 000–2 910 000)**	**2939·0<br>(2588·2–3386·2)**	1 820 000<br>(1 410 000–2 310 000)	2246·8<br>(1725·2–2832·4)	235 000<br>(175 000–296 000)	286·9<br>(215·7–361·6)	565 000<br>(445 000–751 000)	707·4<br>(560·8–923·6)
Seychelles	4 310<br>(3 650–5 020)	3533·1<br>(3050·0–4097·1)	2 940<br>(2 300–3 740)	2496·9<br>(1953·3–3139·0)	369<br>(274–465)	318·6<br>(238·2–402·2)	838<br>(666–1 110)	724·6<br>(579·1–955·1)
Sri Lanka	883 000<br>(765 000–1 020 000)	3285·0<br>(2863·1–3794·4)	520 000<br>(399 000–658 000)	1950·3<br>(1507·1–2463·3)	69 000<br>(50 800–88 900)	264·5<br>(196·9–337·3)	180 000<br>(142 000–241 000)	678·7<br>(544·9–906·4)
Thailand	**4 070 000<br>(3 440 000–4 690 000)**	**3730·9<br>(3194·2–4283·0)**	**1 860 000<br>(1 430 000–2 370 000)**	**1715·9<br>(1326·6–2184·9)**	320 000<br>(241 000–410 000)	301·1<br>(228·4–383·2)	772 000<br>(617 000–1 020 000)	715·1<br>(574·6–946·6)
Timor-Leste	25 500<br>(22 100–29 500)	2982·9<br>(2598·3–3435·2)	14 200<br>(10 800–18 000)	1707·5<br>(1300·1–2165·0)	1 880<br>(1 390–2 400)	228·2<br>(171·2–290·8)	5 170<br>(4 130–6 730)	633·2<br>(510·4–813·1)
Viet Nam	3 220 000<br>(2 780 000–3 770 000)	3116·7<br>(2757·3–3629·1)	1 510 000<br>(1 160 000–1 940 000)	1543·1<br>(1183·9–1978·8)	230 000<br>(174 000–291 000)	234·4<br>(178·5–297·9)	624 000<br>(495 000–819 000)	643·6<br>(514·5–833·9)
Sub-Saharan Africa	18 400 000<br>(16 000 000–21 200 000)	3654·2<br>(3237·5–4155·8)	9 160 000<br>(7 120 000–11 500 000)	1913·2<br>(1475·0–2418·1)	1 840 000<br>(1 380 000–2 290 000)	373·8<br>(286·1–473·4)	3 280 000<br>(2 630 000–4 330 000)	696·5<br>(557·8–909·6)
Central sub-Saharan Africa	2 020 000<br>(1 740 000–2 300 000)	3435·1<br>(3038·9–3883·4)	1 130 000<br>(879 000–1 420 000)	2009·4<br>(1552·8–2570·1)	196 000<br>(146 000–247 000)	346·4<br>(263·6–444·1)	359 000<br>(287 000–478 000)	660·8<br>(527·0–869·7)

Angola	441 000 (375 000–504 000)	3500·9 (3058·6–3986·5)	355 000 (271 000–450 000)	2905·3 (2261·5–3680·5)	43 200 (32 300–54 900)	358·9 (273·3–463·3)	77 600 (62 700–103 000)	672·1 (545·1–876·8)
Central African Republic	79 100 (67 700–91 200)	3344·4 (2998·8–3831·3)	33 500 (25 400–43 100)	1534·9 (1163·4–1962·1)	7 250 (5 430–9 140)	323·5 (247·3–410·4)	13 900 (11 100–18 500)	644·4 (519·4–846·1)
Congo (Brazzaville)	108 000 (92 200–124 000)	3667·1 (3216·3–4120·4)	69 900 (54 600–88 500)	2467·8 (1904·0–3129·2)	11 400 (8 560–14 200)	403·0 (310·2–508·8)	19 200 (15 400–25 600)	704·9 (561·8–936·1)
DR Congo	1 320 000 (1 130 000–1 510 000)	3382·1 (2984·2–3845·1)	630 000 (487 000–804 000)	1706·0 (1299·9–2195·8)	127 000 (93 500–160 000)	335·0 (252·3–429·2)	236 000 (188 000–318 000)	651·8 (517·6–864·0)
Equatorial Guinea	20 600 (17 700–23 700)	3865·2 (3377·7–4404·5)	12 900 (10 100–16 300)	2511·9 (1955·7–3147·7)	2 310 (1 750–2 860)	437·5 (337·6–554·9)	3 570 (2 870–4 720)	718·9 (577·4–943·7)
Gabon	44 800 (39 200–50 900)	3914·8 (3488·9–4403·0)	25 400 (19 800–31 900)	2298·9 (1791·0–2865·0)	5 070 (3 800–6 380)	458·4 (352·7–584·7)	8 010 (6 400–10 500)	739·0 (590·9–979·6)
Eastern sub-Saharan Africa	6 130 000 (5 320 000–7 030 000)	3453·8 (3058·5–3915·6)	3 050 000 (2 370 000–3 810 000)	1807·5 (1393·1–2286·5)	637 000 (478 000–796 000)	367·4 (282·0–465·9)	1 120 000 (900 000–1 480 000)	676·4 (542·8–887·2)
Burundi	164 000 (141 000–190 000)	3263·1 (2856·4–3720·7)	59 200 (45 700–75 100)	1249·0 (955·6–1602·7)	15 400 (11 600–19 600)	318·3 (244·1–403·4)	29 600 (23 600–39 600)	637·1 (509·6–840·2)
Comoros	18 200 (15 700–21 000)	3523·2 (3079·9–4004·6)	8 260 (6 330–10 500)	1645·5 (1253·1–2097·9)	1 840 (1 400–2 320)	366·0 (281·0–466·7)	3 310 (2 670–4 350)	667·8 (537·8–876·9)
Djibouti	24 600 (20 500–28 100)	3538·0 (3045·7–4026·3)	13 000 (10 000–16 100)	1969·1 (1542·0–2471·5)	2 570 (1 910–3 210)	386·4 (297·6–492·0)	4 470 (3 560–5 940)	703·5 (564·5–917·3)
Eritrea	98 200 (84 200–112 000)	3312·5 (2927·6–3775·7)	40 200 (30 800–51 700)	1465·1 (1119·0–1908·4)	9 080 (6 850–11 500)	317·9 (245·7–404·5)	17 600 (14 100–23 700)	650·5 (524·3–861·8)
Ethiopia	1 490 000 (1 280 000–1 710 000)	3360·6 (2939·9–3832·0)	1 100 000 (863 000–1 370 000)	2555·3 (1983·5–3221·3)	164 000 (123 000–205 000)	371·1 (285·5–469·6)	285 000 (227 000–379 000)	684·3 (543·4–899·1)
Kenya	868 000 (751 000–1 000 000)	3563·0 (3160·0–4052·4)	571 000 (445 000–718 000)	2467·1 (1909·3–3094·0)	99 500 (74 500–124 000)	415·1 (318·1–523·3)	161 000 (128 000–214 000)	711·6 (566·0–929·2)
Madagascar	407 000 (350 000–465 000)	3324·2 (2932·1–3754·9)	124 000 (93 100–159 000)	1091·8 (831·2–1405·8)	38 300 (28 800–48 500)	328·6 (252·6–419·7)	72 500 (58 300–95 600)	649·1 (519·9–843·0)
Malawi	275 000 (238 000–313 000)	3526·8 (3124·9–4024·6)	103 000 (78 900–131 000)	1393·5 (1061·8–1787·7)	27 900 (21 300–35 100)	363·5 (281·2–462·3)	49 000 (39 500–64 600)	666·3 (539·8–880·9)
Mozambique	407 000 (352 000–468 000)	3454·8 (3040·6–3933·6)	149 000 (115 000–192 000)	1346·7 (1027·9–1743·7)	39 600 (29 300–50 400)	346·9 (260·4–440·3)	72 100 (57 700–95 100)	656·4 (532·5–866·2)
Rwanda	231 000 (200 000–264 000)	3490·6 (3070·2–3963·1)	72 700 (55 500–93 000)	1169·4 (887·8–1509·5)	22 600 (16 900–28 500)	352·7 (269·2–452·1)	40 800 (32 500–53 300)	659·8 (525·9–847·0)

Somalia	241 000 (208 000–273 000)	3328·8 (2933·8–3807·2)	82 100 (62 700–105 000)	1232·4 (940·6–1591·4)	22 400 (16 700–28 300)	319·0 (245·8–404·8)	42 300 (34 100–55 600)	639·6 (515·6–830·4)
South Sudan	147 000 (125 000–168 000)	3442·5 (3008·0–3915·4)	43 300 (33 200–55 300)	1073·5 (824·5–1380·6)	14 500 (10 900–18 300)	357·0 (274·5–452·1)	25 800 (20 900–33 900)	653·9 (528·3–846·8)
Uganda	535 000 (462 000–614 000)	3482·1 (3097·1–3987·3)	191 000 (145 000–242 000)	1314·5 (998·0–1701·0)	52 800 (39 500–65 700)	351·8 (269·2–445·9)	94 300 (75 400–125 000)	656·8 (527·7–861·5)
Tanzania	955 000 (824 000–1 090 000)	3579·6 (3151·8–4099·6)	381 000 (290 000–483 000)	1490·7 (1132·4–1893·0)	99 500 (74 600–125 000)	382·0 (291·3–486·7)	174 000 (141 000–232 000)	691·8 (559·7–917·4)
Zambia	262 000 (227 000–302 000)	3539·7 (3108·3–4061·4)	111 000 (85 600–140 000)	1584·1 (1211·8–1999·8)	27 000 (20 100–33 400)	373·4 (284·5–476·8)	46 300 (37 500–60 600)	675·2 (546·0–870·6)
Southern sub-Saharan Africa	2 350 000 (2 070 000–2 710 000)	3924·9 (3496·6–4486·1)	1 590 000 (1 240 000–2 010 000)	2746·8 (2134·5–3441·4)	292 000 (219 000–370 000)	494·8 (376·1–633·6)	449 000 (355 000–592 000)	777·5 (618·1–1018·8)
Botswana	59 800 (51 800–68 800)	3927·0 (3479·6–4491·2)	36 500 (28 700–45 600)	2500·2 (1950·0–3160·4)	7 010 (5 270–8 750)	469·0 (357·9–594·2)	10 900 (8 740–14 400)	760·3 (611·1–1000·9)
Eswatini	24 900 (21 700–28 500)	4045·4 (3588·1–4586·2)	13 100 (10 100–16 700)	2233·2 (1720·1–2825·3)	3 000 (2 270–3 720)	494·3 (381·9–626·8)	4 490 (3 600–5 870)	769·7 (617·1–1009·1)
Lesotho	49 800 (43 000–56 600)	3760·1 (3294·8–4274·2)	27 900 (21 700–35 200)	2195·8 (1707·9–2738·3)	5 420 (4 110–6 780)	418·7 (321·8–531·6)	8 910 (7 070–11 700)	709·5 (564·7–936·8)
Namibia	55 200 (48 000–63 500)	3696·0 (3239·2–4229·0)	29 500 (23 100–36 700)	2034·3 (1581·3–2522·2)	6 020 (4 530–7 480)	408·6 (311·6–516·7)	10 200 (8 200–13 500)	712·9 (576·2–943·9)
South Africa	1 890 000 (1 670 000–2 180 000)	3985·1 (3543·8–4566·6)	**1 390 000 (1 080 000–1 750 000)**	**2999·6 (2332·9–3757·3)**	**242 000 (182 000–309 000)**	**517·7 (392·4–663·8)**	366 000 (289 000–484 000)	795·5 (630·8–1043·4)
Zimbabwe	268 000 (234 000–305 000)	3587·4 (3194·6–4064·9)	98 000 (75 500–126 000)	1384·3 (1066·5–1774·8)	28 000 (20 900–35 200)	382·2 (291·1–490·1)	48 200 (38 700–63 200)	686·0 (550·7–895·5)
Western sub-Saharan Africa	7 900 000 (6 820 000–9 130 000)	3804·8 (3366·8–4358·5)	3 390 000 (2 630 000–4 260 000)	1719·2 (1320·6–2173·5)	710 000 (534 000–886 000)	350·6 (268·7–441·4)	1 350 000 (1 080 000–1 780 000)	699·5 (560·1–911·8)
Benin	208 000 (181 000–240 000)	3910·2 (3461·2–4529·7)	90 800 (71 000–114 000)	1779·4 (1363·3–2225·7)	19 300 (14 600–24 100)	370·1 (284·3–468·2)	35 400 (28 400–46 400)	709·1 (568·6–929·7)
Burkina Faso	373 000 (321 000–430 000)	3740·3 (3265·4–4299·8)	94 100 (71 100–120 000)	1006·9 (758·5–1286·4)	32 200 (24 500–41 100)	331·6 (257·3–419·9)	62 500 (50 200–83 100)	672·2 (536·8–880·4)
Cabo Verde	18 000 (15 500–20 600)	3974·3 (3467·5–4544·9)	8 370 (6 520–10 600)	1876·8 (1456·2–2365·6)	1 720 (1 320–2 150)	379·4 (290·8–478·0)	3 150 (2 520–4 090)	710·5 (570·1–914·2)
Cameroon	535 000 (455 000–619 000)	4066·7 (3563·6–4683·9)	198 000 (153 000–246 000)	1588·7 (1218·2–2004·0)	52 200 (39 000–64 100)	405·8 (310·1–508·3)	90 500 (72 300–121 000)	738·0 (591·4–974·8)

Chad	213 000 (181 000–246 000)	3490·6 (3045·8–4007·3)	62 800 (48 600–80 000)	1088·6 (835·4–1410·5)	17 700 (13 300–22 400)	300·2 (232·4–383·7)	37 100 (29 800–48 700)	654·4 (525·4–852·2)
Côte d'Ivoire	456 000 (393 000–534 000)	3815·0 (3416·7–4425·9)	179 000 (140 000–225 000)	1590·8 (1217·8–2018·0)	41 700 (31 400–52 200)	358·5 (275·3–454·1)	76 300 (60 900–101 000)	695·4 (557·4–906·0)
The Gambia	38 900 (33 900–45 000)	3815·0 (3361·1–4403·1)	19 000 (14 600–23 700)	1927·4 (1465·1–2442·8)	3 580 (2 690–4 470)	353·9 (270·6–445·4)	6 750 (5 370–9 020)	697·3 (553·8–928·0)
Ghana	702 000 (603 000–805 000)	3984·9 (3528·1–4561·6)	360 000 (279 000–457 000)	2127·0 (1643·0–2697·6)	65 100 (49 200–80 600)	379·3 (291·9–479·0)	117 000 (93 900–158 000)	710·6 (575·8–946·2)
Guinea	211 000 (182 000–244 000)	3650·5 (3217·1–4207·5)	66 000 (50 500–83 800)	1189·6 (907·5–1532·5)	18 500 (13 700–23 400)	324·8 (244·6–412·7)	36 700 (29 500–48 200)	670·1 (536·2–867·8)
Guinea-Bissau	28 600 (24 700–33 100)	3665·2 (3258·1–4197·7)	10 200 (7 850–12 900)	1397·6 (1063·8–1785·2)	2 440 (1 820–3 060)	319·1 (243·8–404·4)	4 760 (3 830–6 340)	662·6 (536·4–870·2)
Liberia	90 500 (77 800–105 000)	3915·6 (3465·4–4475·5)	35 500 (27 500–44 500)	1622·8 (1255·5–2041·5)	8 530 (6 380–10 700)	377·7 (286·0–479·3)	15 100 (12 100–20 200)	710·0 (570·4–937·5)
Mali	336 000 (287 000–385 000)	3624·6 (3153·8–4141·1)	120 000 (91 500–152 000)	1349·8 (1017·6–1707·3)	28 700 (21 800–36 100)	320·1 (247·2–404·3)	57 600 (46 200–77 000)	662·4 (534·1–878·0)
Mauritania	90 100 (79 100–104 000)	4041·7 (3588·2–4622·2)	36 100 (27 700–46 200)	1664·6 (1265·4–2117·5)	8 760 (6 590–10 900)	401·7 (306·3–507·7)	15 700 (12 400–20 800)	735·5 (580·1–978·8)
Niger	303 000 (258 000–348 000)	3547·2 (3091·2–4052·9)	86 200 (65 400–110 000)	1074·4 (817·4–1393·1)	24 600 (18 200–31 000)	301·6 (228·4–382·9)	51 400 (41 600–67 700)	650·7 (529·1–846·3)
Nigeria	**3 690 000 (3 170 000–4 250 000)**	**3807·3 (3368·5–4347·9)**	1 780 000 (1 380 000–2 240 000)	1934·4 (1485·6–2441·8)	**332 000 (249 000–413 000)**	**349·6 (267·5–438·5)**	637 000 (507 000–843 000)	709·9 (565·1–926·9)
São Tomé and Príncipe	4 590 (3 960–5 280)	3937·5 (3487·6–4528·4)	2 700 (2 100–3 400)	2395·9 (1864·9–3039·7)	427 (317–526)	376·8 (287·4–473·6)	774 (620–1 010)	710·5 (569·6–930·8)
Senegal	305 000 (262 000–352 000)	3794·5 (3305·7–4331·7)	127 000 (96 500–160 000)	1634·9 (1240·5–2041·9)	27 300 (20 500–34 400)	349·0 (265·0–445·3)	52 600 (42 300–70 100)	689·9 (554·5–907·9)
Sierra Leone	142 000 (123 000–162 000)	3673·1 (3214·7–4176·0)	55 700 (42 900–69 900)	1501·8 (1144·3–1916·0)	12 500 (9 410–15 900)	328·5 (250·9–416·0)	24 600 (19 900–32 500)	676·5 (543·2–884·4)
Togo	154 000 (131 000–177 000)	3750·0 (3284·3–4284·0)	64 100 (49 400–81 500)	1663·3 (1266·2–2147·0)	13 100 (9 790–16 400)	331·8 (255·2–417·4)	25 500 (20 300–33 400)	673·8 (543·4–886·1)

*The estimates for countries with input data (for each site of osteoarthritis) are shown in bold. For example, input data was available for knee osteoarthritis in Vietnam, and so these estimates are bolded. Note that the sum of regional cases will not exactly match global cases due to rounding (and similarly for regions to global).

Supplemental Table 4. Forecast of age-standardised prevalence and total cases for each site of osteoarthritis globally and by region, both sexes, in 2050.

	Age-standardised prevalence (per 100 000)	Cases (millions)						
**Region**	**Hand OA**	**Hip OA**	**Knee OA**	**Other OA**	**Hand OA**	**Hip OA**	**Knee OA**	**Other OA**
Global	2·01 (1·51–2·64)	0·419 (0·315–0·536)	4·33 (3·87–4·94)	0·746 (0·612–0·955)	279 (221–338)	62·6 (49·7–75·5)	642 (574–722)	118 (97·1–143)
Andean Latin America	2·46 (1·91–3·08)	0·395 (0·306–0·496)	4·77 (4·21–5·36)	0·755 (0·614–0·976)	2·47 (1·94–3·02)	0·490 (0·387–0·601)	6·02 (5·04–7·21)	1·01 (0·822–1·28)
Australasia	2·52 (1·93–3·15)	0·810 (0·633–1·02)	4·91 (4·33–5·52)	0·780 (0·629–1·01)	1·90 (1·32–2·64)	0·596 (0·463–0·759)	3·48 (2·83–4·18)	0·601 (0·446–0·804)
Caribbean	2·32 (1·81–2·92)	0·378 (0·287–0·476)	4·57 (4·11–5·17)	0·743 (0·597–0·969)	1·56 (1·25–1·92)	0·323 (0·256–0·384)	3·91 (3·39–4·65)	0·676 (0·569–0·832)
Central Asia	3·88 (2·96–4·84)	0·503 (0·397–0·637)	2·79 (2·48–3·13)	0·742 (0·600–0·967)	5·02 (3·94–6·21)	0·729 (0·535–0·892)	4·43 (3·74–5·33)	1·17 (0·928–1·51)
Central Europe	3·19 (2·49–3·99)	0·566 (0·446–0·716)	3·34 (2·99–3·77)	0·786 (0·638–1·02)	6·12 (4·81–7·46)	1·29 (0·995–1·57)	7·62 (6·76–8·88)	1·95 (1·59–2·37)
Central Latin America	2·71 (2·11–3·40)	0·378 (0·290–0·468)	4·69 (4·20–5·28)	0·771 (0·622–0·997)	11·5 (8·96–13·8)	1·81 (1·41–2·17)	23·4 (20·5–27·3)	4·10 (3·33–5·12)
Central Sub-Saharan Africa	2·01 (1·55–2·57)	0·404 (0·317–0·505)	3·70 (3·29–4·15)	0·697 (0·571–0·907)	3·45 (2·68–4·25)	0·731 (0·548–0·912)	7·19 (5·83–8·77)	1·31 (0·998–1·73)
East Asia	1·59 (1·21–2·04)	0·294 (0·228–0·366)	5·30 (4·68–6·02)	0·741 (0·602–0·952)	44·2 (35·1–54·9)	9·12 (7·08–10·8)	159 (141–176)	26·3 (22·5–32·2)
Eastern Europe	4·23 (3·33–5·36)	0·565 (0·447–0·708)	3·51 (3·14–3·97)	0·784 (0·632–1·02)	16·7 (12·9–20·7)	2·22 (1·66–2·72)	14·0 (12·1–17·1)	3·38 (2·62–4·19)
Eastern Sub-Saharan Africa	1·83 (1·41–2·30)	0·429 (0·337–0·527)	3·75 (3·35–4·22)	0·713 (0·585–0·921)	10·4 (7·99–13·1)	2·45 (1·84–2·98)	23·0 (19·0–26·8)	4·21 (3·22–5·42)
High-income Asia Pacific	3·03 (2·33–3·78)	0·505 (0·386–0·638)	5·70 (5·15–6·46)	0·679 (0·545–0·880)	14·1 (11·3–17·1)	2·33 (1·84–2·80)	25·4 (22·5–28·2)	3·46 (2·94–4·23)

High-income North America	3·10 (2·36–3·88)	0·928 (0·711–1·19)	4·76 (4·24–5·33)	0·808 (0·705–0·954)	26·6 (19·2–34·2)	7·45 (5·85–9·24)	36·1 (31·8–43·5)	6·44 (5·45–7·74)
North Africa and Middle East	1·95 (1·54–2·45)	0·334 (0·260–0·410)	4·03 (3·61–4·55)	0·813 (0·665–1·05)	17·9 (14·6–21·5)	3·98 (3·16–4·65)	49·3 (43·5–55·1)	10·3 (8·43–12·9)
Oceania	1·66 (1·28–2·10)	0·325 (0·248–0·408)	4·25 (3·76–4·84)	0·711 (0·581–0·933)	0·229 (0·180–0·281)	0·0613 (0·0459–0·0753)	0·844 (0·706–1·00)	0·138 (0·111–0·181)
South Asia	2·05 (1·58–2·58)	0·350 (0·273–0·432)	4·09 (3·65–4·61)	0·728 (0·594–0·938)	47·8 (38·4–57·4)	10·9 (8·64–13·2)	134 (119–153)	24·8 (20·2–30·7)
Southeast Asia	1·89 (1·46–2·40)	0·308 (0·238–0·382)	3·39 (3·01–3·86)	0·717 (0·583–0·926)	14·6 (11·3–18·5)	3·76 (2·94–4·44)	42·2 (37·7–47·7)	9·58 (8·07–11·6)
Southern Latin America	2·48 (1·92–3·08)	0·734 (0·565–0·937)	4·73 (4·22–5·33)	0·744 (0·602–0·971)	3·67 (2·87–4·50)	1·00 (0·805–1·24)	6·40 (5·52–7·34)	1·05 (0·839–1·28)
Southern Sub-Saharan Africa	2·73 (2·13–3·44)	0·540 (0·418–0·681)	4·07 (3·64–4·63)	0·803 (0·647–1·04)	3·17 (2·50–3·84)	0·685 (0·512–0·834)	5·41 (4·56–6·35)	1·08 (0·834–1·41)
Tropical Latin America	2·62 (2·04–3·28)	0·426 (0·329–0·528)	4·66 (4·19–5·25)	0·785 (0·634–1·02)	8·96 (7·14–10·9)	1·93 (1·54–2·29)	21·8 (19·7–24·5)	3·98 (3·32–4·71)
Western Europe	2·29 (1·78–2·88)	0·786 (0·608–0·997)	4·24 (3·79–4·75)	0·733 (0·594–0·955)	23·5 (18·1–28·8)	8·16 (6·46–9·86)	42·5 (38·2–48·2)	7·93 (6·46–9·71)
Western Sub-Saharan Africa	1·74 (1·34–2·19)	0·407 (0·320–0·501)	4·13 (3·69–4·68)	0·737 (0·604–0·949)	11·1 (8·70–13·9)	2·54 (1·86–3·11)	27·7 (22·8–33·0)	4·79 (3·64–6·01)

Note that the sum of regional cases will not exactly match global cases due to rounding.

Supplemental Table 5. Decomposition analysis showing the contribution of population growth, population ageing, and age-standardised prevalence to the total forecasted percent change in cases between 2020 and 2050 by region and globally.

Region	Population growth (%)	Population ageing (%)	Age-standardised prevalence (%)	Total % change in cases	Population growth (%)	Population ageing (%)	Age-standardised prevalence (%)	Total % change in cases
	Knee osteoarthritis				Hip osteoarthritis			
Global	47.5	25.7	1.1	74.9	48.4	31.9	-0.6	78.6
Central Asia	54.0	40.6	4.2	104.8	56.3	48.1	7.8	106.0
Central Europe	-11.9	21.7	3.4	14.2	-12.7	35.3	6.6	17.1
Eastern Europe	-7.7	22.6	2.8	19.0	-8.0	38.1	5.5	22.5
Australasia	37.4	12.4	3.0	53.1	38.6	15.5	7.2	63.1
High-income Asia Pacific	-10.8	20.7	3.6	13.5	-11.0	25.0	7.0	22.4
High-income North America	17.9	7.2	1.1	26.9	18.4	10.4	3.2	36.3
Southern Latin America	35.4	25.4	3.8	66.3	36.8	27.9	9.2	76.5
Western Europe	6.1	13.8	2.4	22.5	6.5	16.6	5.6	30.0
Andean Latin America	74.8	39.8	5.7	123.1	78.5	45.3	12.6	131.5
Caribbean	33.1	26.6	2.8	64.5	34.3	33.3	6.0	69.9
Central Latin America	62.0	38.6	6.0	110.2	64.8	44.5	12.8	113.1
Tropical Latin America	40.4	44.9	6.1	91.9	42.6	53.9	13.2	98.5
North Africa and Middle East	95.6	66.1	9.7	172.4	98.5	69.7	22.3	189.1
South Asia	81.7	39.5	10.2	132.5	85.2	39.3	25.5	144.2
East Asia	4.0	32.7	6.7	42.9	3.3	51.6	16.4	65.3
Oceania	115.9	34.2	6.9	159.2	119.2	40.7	14.0	174.1
Southeast Asia	48.7	38.3	6.2	93.9	52.3	53.1	15.7	114.2
Central sub-Saharan Africa	199.1	28.9	16.4	257.2	208.2	31.9	34.4	277.2

Eastern sub-Saharan Africa	214.1	38.2	18.1	276.7	221.1	35.0	37.2	287.5
Southern sub-Saharan Africa	79.0	43.7	5.6	130.9	81.0	45.9	11.7	137.0
Western sub-Saharan Africa	200.9	28.9	16.9	252.2	208.6	28.3	33.9	261.0
	**Hand osteoarthritis**				**Other osteoarthritis**			
Global	45.9	29.9	-13.8	48.6	49.7	35.0	5.3	95.1
Central Asia	52.2	39.7	-0.6	62.3	55.3	47.7	2.4	112.9
Central Europe	-11.9	22.5	0.1	-7.1	-12.1	28.7	2.5	21.9
Eastern Europe	-8.1	22.1	3.1	17.5	-7.7	30.3	2.1	28.2
Australasia	37.4	15.7	0.0	57.0	38.4	18.8	2.1	61.9
High-income Asia Pacific	-11.0	25.0	3.2	12.6	-11.0	29.9	1.1	21.8
High-income North America	18.0	10.9	-0.1	38.0	18.5	13.6	1.0	34.8
Southern Latin America	34.9	26.8	0.1	75.3	36.1	29.7	2.8	72.1
Western Europe	5.9	14.6	2.0	23.5	6.5	18.6	1.7	28.6
Andean Latin America	73.7	42.7	0.3	73.1	77.3	48.2	4.3	138.6
Caribbean	33.3	29.9	0.0	26.7	33.9	34.0	1.7	74.8
Central Latin America	61.4	41.8	0.1	72.5	63.9	47.9	4.4	125.6
Tropical Latin America	40.4	50.5	0.0	35.5	41.9	56.7	4.6	110.2
North Africa and Middle East	96.6	70.1	5.5	104.9	97.8	79.8	7.3	195.0
South Asia	81.0	46.4	0.1	61.6	84.4	51.5	8.4	153.6
East Asia	3.9	53.9	0.1	28.3	3.4	55.5	5.5	70.5
Oceania	117.3	45.5	0.0	89.3	118.1	46.4	4.0	177.5
Southeast Asia	50.0	51.8	0.1	20.9	51.7	58.1	5.5	122.5
Central sub-Saharan Africa	192.8	29.8	0.3	209.1	199.2	34.5	11.1	269.6
Eastern sub-Saharan Africa	207.6	40.7	0.7	245.9	212.3	39.8	12.6	280.1

Southern sub-Saharan Africa	77.8	44.4	0.1	100.8	80.2	50.3	3.6	143.2
Western sub-Saharan Africa	194.9	33.4	0.6	231.5	200.7	32.2	11.9	258.6

Note that the sum of each decomposition component will not sum exactly to the "Total % change in cases" column due to rounding.

Data input sources

Hand osteoarthritis

Al-Arfaj AS, Al-Boukai A. Prevalence of radiographic osteoarthritis of the hands in Saudi Arabia. Rheumatol Int. 2002; 22(5): 208-12.

Andrianakos AA, Kontelis LK, Karamitsos DG, Aslanidis SI, Georgountzos AI, Kaziolas GO, Pantelidou KV, Vafiadou EV, Dantis PC, ESORDIG Study Group. Prevalence of symptomatic knee, hand, and hip osteoarthritis in Greece. The ESORDIG study. J Rheumatol. 2006; 33(12): 2507-13.

Aspelund G, Gunnarsdottir S, Jonsson P, Jonsson H. Hand osteoarthritis in the elderly. Application of clinical criteria. Scand J Rheumatol. 1996; 25(1): 34-6.

Bae KJ, Gong HS, Kim KW, Kim TK, Chang CB, Jang HC, Baek GH. Evaluation of femoral neck bone mineral density and radiographic hand and knee osteoarthritis in a Korean elderly population. Clin Orthop Surg. 2014; 6(3): 343-9.

Bagge E, Eden S, Rosen T, Bengtsson BA. The prevalence of radiographic osteoarthritis is low in elderly patients with growth hormone deficiency. Acta Endocrinol. 1993; 129(4): 296-300.

Bovenzi M, Petronio L, DiMarino F. Epidemiological survey of shipyard workers exposed to hand-arm vibration. Int Arch Occup Environ Health. 1980; 46(3): 251-66.

Branco JC, Rodrigues AM, Gouveia N, Eusébio M, Ramiro S, Machado PM, da Costa LP, Mourão AF, Silva I, Laires P, Sepriano A, Araújo F, Gonçalves S, Coelho PS, Tavares V, Cerol J, Mendes JM, Carmona L, Canhão H, EpiReumaPt study group. Prevalence of rheumatic and musculoskeletal diseases and their impact on health-related quality of life, physical function and mental health in Portugal: results from EpiReumaPt- a national health survey. RMD Open. 2016; 2(1): e000166.

Brighton SW, de la Harpe AL, Van Staden DA. The prevalence of osteoarthrosis in a rural African community. Br J Rheumatol. 1985; 24(4): 321-5.

Cakır N, Pamuk ÖN, Derviş E, Imeryüz N, Uslu H, Benian Ö, Elelçi E, Erdem G, Sarvan FO, Senocak M. The prevalences of some rheumatic diseases in western Turkey: Havsa study. Rheumatol Int. 2012; 32(4): 895-908.

Carman WJ. Factors associated with pain and osteoarthritis in the Tecumseh Community Health Study. Semin Arthritis Rheum. 1989; 18(4 Suppl 2): 10-3.

Carmona L, Ballina J, Gabriel R, Laffon A, EPISER Study Group. The burden of musculoskeletal diseases in the general population of Spain: results from a national survey. Ann Rheum Dis. 2001; 60(11): 1040-5.

Caspi D, Flusser G, Farber I, Ribak J, Leibovitz A, Habot B, Yaron M, Segal R. Clinical, radiologic, demographic, and occupational aspects of hand osteoarthritis in the elderly. Semin Arthritis Rheum. 2001; 30(5): 321-31.

Castell MV, van der Pas S, Otero A, Siviero P, Dennison E, Denkinger M, Pedersen N, Sanchez-Martinez M, Queipo R, van Schoor N, Zambon S, Edwards M, Peter R, Schaap L, Deeg D. Osteoarthritis and

frailty in elderly individuals across six European countries: results from the European Project on OSteoArthritis (EPOSA). BMC Musculoskelet Disord. 2015; 16: 359.

Cauley JA, Kwoh CK, Egeland G, Nevitt MC, Cooperstein L, Rohay J, Towers A, Gutai JP. Serum sex hormones and severity of osteoarthritis of the hand. J Rheumatol. 1993; 20(7): 1170-5.

Chaiamnuay P, Darmawan J, Muirden KD, Assawatanabodee P. Epidemiology of rheumatic disease in rural Thailand: a WHO-ILAR COPCORD study. Community Oriented Programme for the Control of Rheumatic Disease. J Rheumatol. 1998; 25(7): 1382-7.

Cho HJ, Morey V, Kang JY, Kim KW, Kim TK. Prevalence and Risk Factors of Spine, Shoulder, Hand, Hip, and Knee Osteoarthritis in Community-dwelling Koreans Older Than Age 65 Years. Clin Orthop Relat Res. 2015; 473(10): 3307-3314.

Dahaghin S, Bierma-Zeinstra SM, Ginai AZ, Pols HA, Hazes JM, Koes BW. Prevalence and pattern of radiographic hand osteoarthritis and association with pain and disability (the Rotterdam study). Ann Rheum Dis. 2005; 64(5): 682-7.

Davatchi F, Jamshidi A-R, Banihashemi AT, Gholami J, Forouzanfar MH, Akhlaghi M, Barghamdi M, Noorolahzadeh E, Khabazi A-R, Salesi M, Salari A-H, Karimifar M, Essalat-Manesh K, Hajialiloo M, Soroosh M, Farzad F, Moussavi H-R, Samadi F, Ghaznavi K, Asgharifard H, Zangiabadi A-H, Shahram F, Nadji A, Akbarian M, Gharibdoost F. WHO-ILAR COPCORD Study (Stage 1, Urban Study) in Iran. J Rheumatol. 2008; 35(7): 1384.

Dillon CF, Hirsch R, Rasch EK, Gu Q. Symptomatic hand osteoarthritis in the United States: prevalence and functional impairment estimates from the third U.S. National Health and Nutrition Examination Survey, 1991-1994. Am J Phys Med Rehabil. 2007; 86(1): 12-21.

Duncan R, Francis RM, Collerton J, Davies K, Jagger C, Kingston A, Kirkwood T, Robinson L, Birrell F. Prevalence of arthritis and joint pain in the oldest old: findings from the Newcastle 85+ study. Age Ageing. 2011; 40(6): 752-5.

Englund M, Haugen IK, Guermazi A, Roemer FW, Niu J, Neogi T, Aliabadi P, Felson DT. Evidence that meniscus damage may be a component of osteoarthritis: the Framingham study. Osteoarthr Cartil. 2016; 24(2): 270-3.

Garessus ED, de Mutsert R, Visser AW, Rosendaal FR, Kloppenburg M. No association between impaired glucose metabolism and osteoarthritis. Osteoarthr Cartil. 2016; 24(9): 1541-7.

Garnero P, Sornay-Rendu E, Arlot M, Christiansen C, Delmas PD. Association between spine disc degeneration and type II collagen degradation in postmenopausal women: the OFELY study. Arthritis Rheum. 2004; 50(10): 3137-44.

Goekoop RJ, Kloppenburg M, Kroon HM, Dirkse LE, Huizinga TW, Westendorp RG, Gussekloo J. Determinants of absence of osteoarthritis in old age. Scand J Rheumatol. 2011; 40(1): 68-73.

Goode AP, Nelson AE, Kraus VB, Renner JB, Jordan JM. Biomarkers reflect differences in osteoarthritis phenotypes of the lumbar spine: the Johnston County Osteoarthritis Project. Osteoarthr Cartil. 2017; 25(10): 1672-1679.

Granados Y, Cedeno L, Rosillo C, Berbin S, Azocar M, Molina ME, Lara O, Sanchez G, Pelaez-Ballestas I. Prevalence of musculoskeletal disorders and rheumatic diseases in an urban community in Monagas State, Venezuela: a COPCORD study. Clin Rheumatol. 2015; 34(5): 871-7.

Guevara-Pacheco S, Feicán-Alvarado A, Sanín LH, Vintimilla-Ugalde J, Vintimilla-Moscoso F, Delgado-Pauta J, Lliguisaca-Segarra A, Dután-Erráez H, Guevara-Mosquera D, Ochoa-Robles V, Cardiel MH, Peláez-Ballestas I. Prevalence of musculoskeletal disorders and rheumatic diseases in Cuenca, Ecuador: a WHO-ILAR COPCORD study. Rheumatol Int. 2016; 36(9): 1195-1204.

Haara MM, Arokoski JP, Kroger H, Karkkainen A, Manninen P, Knekt P, Impivaara O, Heliovaara M. Association of radiological hand osteoarthritis with bone mineral mass: a population study. Rheumatology (Oxford). 2005; 44(12): 1549-54.

Hart DJ, Spector TD, Brown P, Wilson P, Doyle DV, Silman AJ. Clinical signs of early osteoarthritis: reproducibility and relation to x ray changes in 541 women in the general population. Ann Rheum Dis. 1991; 50(7): 467-70.

Haugen IK, Englund M, Aliabadi P, Niu J, Clancy M, Kvien TK, Felson DT. Prevalence, incidence and progression of hand osteoarthritis in the general population: the Framingham Osteoarthritis Study. Ann Rheum Dis. 2011; 70(9): 1581-6.

Haugen IK, Magnusson K, Turkiewicz A, Englund M. The Prevalence, Incidence, and Progression of Hand Osteoarthritis in Relation to Body Mass Index, Smoking, and Alcohol Consumption. J Rheumatol. 2017; 44(9): 1402-1409.

Hochberg MC, Lane NE, Pressman AR, Genant HK, Scott JC, Nevitt MC. The association of radiographic changes of osteoarthritis of the hand and hip in elderly women. J Rheumatol. 1995; 22(12): 2291-4.

Jonsson H, Helgadottir GP, Aspelund T, Sverrisdottir JE, Eiriksdottir G, Sigurdsson S, Eliasson GJ, Jonsson A, Ingvarsson T, Harris TB, Launer L, Gudnason V. The use of digital photographs for the diagnosis of hand osteoarthritis: the AGES-Reykjavik study. BMC Musculoskelet Disord. 2012; 13: 20.

Jonsson H. Age related prevalence of hand osteoarthritis diagnosed by photography (HOASCORE). BMC Musculoskelet Disord. 2017; 18(1): 508.

Jørgensen KT, Pedersen BV, Nielsen NM, Hansen AV, Jacobsen S, Frisch M. Socio-demographic factors, reproductive history and risk of osteoarthritis in a cohort of 4.6 million Danish women and men. Osteoarthr Cartil. 2011; 19(10): 1176-82.

Kalichman L, Malkin I, Batsevich V, Kobyliansky E. Radiographic hand osteoarthritis in two ethnic groups living in the same geographic area. Rheumatol Int. 2010; 30(11): 1533-6.

Kodama R, Muraki S, Oka H, Iidaka T, Teraguchi M, Kagotani R, Asai Y, Yoshida M, Morizaki Y, Tanaka S, Kawaguchi H, Nakamura K, Akune T, Yoshimura N. Prevalence of hand osteoarthritis and its relationship to hand pain and grip strength in Japan: The third survey of the ROAD study. Mod Rheumatol. 2016; 26(5): 767-73.

Kwok WY, Kloppenburg M, Rosendaal FR, van Meurs JB, Hofman A, Bierma-Zeinstra SM. Erosive hand osteoarthritis: its prevalence and clinical impact in the general population and symptomatic hand osteoarthritis. Ann Rheum Dis. 2011; 70(7): 1238-42.

Macias-Hernandez SI, Zepeda-Borbon ER, Lara-Vazquez BI, Cuevas-Quintero NM, Morones-Alba JD, Cruz-Medina E, Nava-Bringas TI, Miranda-Duarte A. Prevalence of Clinical and Radiological Osteoarthritis in Knee, Hip, and Hand in an Urban Adult Population of Mexico City. Reumatol Clin. 2018.

Mannoni A, Briganti MP, Di Bari M, Ferrucci L, Costanzo S, Serni U, Masotti G, Marchionni N. Epidemiological profile of symptomatic osteoarthritis in older adults: a population based study in Dicomano, Italy. Ann Rheum Dis. 2003; 62(6): 576-8.

Mannoni A, Briganti MP, Di Bari M, Ferrucci L, Serni U, Masotti G, Marchionni N. Prevalence of symptomatic hand osteoarthritis in community-dwelling older persons: the ICARe Dicomano study. Insufficienza Cardiaca negli Anzizni Residenti a Dicomano. Osteoarthr Cartil. 2000; 8 Suppl A: S11-3.

Marshall M, Peat G, Nicholls E, van der Windt D, Myers H, Dziedzic K. Subsets of symptomatic hand osteoarthritis in community-dwelling older adults in the United Kingdom: prevalence, inter-relationships, risk factor profiles and clinical characteristics at baseline and 3-years. Osteoarthr Cartil. 2013; 21(11): 1674-84.

Mathiessen A, Slatkowsky-Christensen B, Kvien TK, Haugen IK, Berner Hammer H. Ultrasound-detected osteophytes predict the development of radiographic and clinical features of hand osteoarthritis in the same finger joints 5 years later. RMD Open. 2017; 3(2): e000505.

Miura H, Kawano T, Takasugi S, Manabe T, Hosokawa A, Iwamoto Y. Two subtypes of radiographic osteoarthritis in the distal interphalangeal joint of the hand. J Orthop Sci. 2008; 13(6): 487-91.

Nakamura R, Ono Y, Horii E, Tsunoda K, Takeuchi Y. The aetiological significance of work-load in the development of osteoarthritis of the distal interphalangeal joint. J Hand Surg. 1993; 18(4): 540-2.

Poole J, Sayer AA, Cox V, Cooper C, Kuh D, Hardy R, Wadsworth M. Birth weight, osteoarthritis of the hand, and cardiovascular disease in men. Ann Rheum Dis. 2003; 62(10): 1029.

Recht L, Helin P, Rasmussen JO, Jacobsen J, Lithman T, Scherstén B. Hand handicap and rheumatoid arthritis in a fish-eating society (the Faroe Islands). J Intern Med. 1990; 227(1): 49-55.

Salaffi F, De Angelis R, Grassi W, MArche Pain Prevalence, INvestigation Group (MAPPING) study. Prevalence of musculoskeletal conditions in an Italian population sample: results of a regional community-based study. I. The MAPPING study. Clin Exp Rheumatol. 2005; 23(6): 819-28.

Sanchez-Santos MT, Judge A, Gulati M, Spector TD, Hart DJ, Newton JL, Arden NK, Kluzek S. Association of metabolic syndrome with knee and hand osteoarthritis: A community-based study of women. Semin Arthritis Rheum. 2018.

Schneider DL, Barrett-Connor E, Morton DJ, Weisman M. Bone mineral density and clinical hand osteoarthritis in elderly men and women: the Rancho Bernardo study. J Rheumatol. 2002; 29(7): 1467-72.

Schouten JS, van den Ouweland FA, Valkenburg HA. Natural menopause, oophorectomy, hysterectomy and the risk of osteoarthritis of the dip joints. Scand J Rheumatol. 1992; 21(4): 196-200.

Szoeke CEI, Cicuttini FM, Guthrie JR, Clark MS, Dennerstein L. Factors affecting the prevalence of osteoarthritis in healthy middle-aged women: data from the longitudinal Melbourne Women's Midlife Health Project. Bone. 2006; 39(5): 1149-1155.

Turkiewicz A, Petersson IF, Björk J, Hawker G, Dahlberg LE, Lohmander LS, Englund M. Current and future impact of osteoarthritis on health care: a population-based study with projections to year 2032. Osteoarthr Cartil. 2014; 22(11): 1826-32.

Van Saase JL, van Romunde LK, Cats A, Vandenbroucke JP, Valkenburg HA. Epidemiology of osteoarthritis: Zoetermeer survey. Comparison of radiological osteoarthritis in a Dutch population with that in 10 other populations. Ann Rheum Dis. 1989; 48(4): 271-80.

Vega-Hinojosa O, Cardiel MH, Ochoa-Miranda P. Prevalence of musculoskeletal manifestations and related disabilities in a Peruvian urban population living at high altitude. COPCORD Study. Stage I. Reumatol Clin. 2018; 14(5): 278-284.

Veronese N, Maggi S, Noale M, Trevisan C, De Rui M, Bolzetta F, Zambon S, Sartori L, Musacchio E, Perissinotto E, Crepaldi G, Manzato E, Sergi G. Serum dehydroepiandrosterone sulfate and osteoarthritis in older people: the Pro.V.A. study. Clin Rheumatol. 2016; 35(10): 2609-14.

Wang F, Shi L, Xue QY. Association of Metabolic Factors with Symptomatic Hand Osteoarthritis in the Chinese Han Population Aged 40 Years and above. Chin Med J. 2016; 129(19): 2301-7.

Wang Y, Peng R, Ma R. Epidemiological investigation of osteoarthritis in middle-aged mongolian and senior residents of the inner mongolia autonomous region. Iran Red Crescent Med J. 2013; 15(10): e8303.

Yoshida S, Aoyagi K, Felson DT, Aliabadi P, Shindo H, Takemoto T. Comparison of the prevalence of radiographic osteoarthritis of the knee and hand between Japan and the United States. J Rheumatol. 2002; 29(7): 1454-8.

Zeng QY, Chen R, Xiao ZY, Huang S-B, Liu Y, Xu JC, Chen SL, Darmawan J, Couchman KG, Wigley RD, Muirden KD. Low prevalence of knee and back pain in southeast China; the Shantou COPCORD study. J Rheumatol. 2004; 31(12): 2439-43.

Zhang J, Song L, Wei J, Zhang A, Dong H, Wen H, Luo J, Liu G. Prevalence of and risk factors for the occurrence of symptomatic osteoarthritis in rural regions of Shanxi Province, China. Int J Rheum Dis. 2016; 19(8): 781-789.

Zhang Y, Xu L, Nevitt MC, Niu J, Goggins JP, Aliabadi P, Yu W, Lui LY, Felson DT. Lower prevalence of hand osteoarthritis among Chinese subjects in Beijing compared with white subjects in the United States: the Beijing Osteoarthritis Study. Arthritis Rheum. 2003; 48(4): 1034-40.

Hip osteoarthritis

Andrianakos AA, Kontelis LK, Karamitsos DG, Aslanidis SI, Georgountzos AI, Kaziolas GO, Pantelidou KV, Vafiadou EV, Dantis PC, ESORDIG Study Group. Prevalence of symptomatic knee, hand, and hip osteoarthritis in Greece. The ESORDIG study. J Rheumatol. 2006; 33(12): 2507-13.

Branco JC, Rodrigues AM, Gouveia N, Eusébio M, Ramiro S, Machado PM, da Costa LP, Mourão AF, Silva I, Laires P, Sepriano A, Araújo F, Gonçalves S, Coelho PS, Tavares V, Cerol J, Mendes JM, Carmona

L, Canhão H, EpiReumaPt study group. Prevalence of rheumatic and musculoskeletal diseases and their impact on health-related quality of life, physical function and mental health in Portugal: results from EpiReumaPt- a national health survey. RMD Open. 2016; 2(1): e000166.

Chaaya M, Slim ZN, Habib RR, Arayssi T, Dana R, Hamdan O, Assi M, Issa Z, Uthman I. High burden of rheumatic diseases in Lebanon: a COPCORD study. Int J Rheum Dis. 2012; 15(2): 136-43.

Cho HJ, Morey V, Kang JY, Kim KW, Kim TK. Prevalence and Risk Factors of Spine, Shoulder, Hand, Hip, and Knee Osteoarthritis in Community-dwelling Koreans Older Than Age 65 Years. Clin Orthop Relat Res. 2015; 473(10): 3307-3314.

Chung CY, Park MS, Lee KM, Lee SH, Kim TK, Kim KW, Park JH, Lee JJ. Hip osteoarthritis and risk factors in elderly Korean population. Osteoarthr Cartil. 2010; 18(3): 312-6.

Cornoni-Huntley J, Huntley RR, Feldman JJ, editors. Health Status and Well-being of the Elderly: National Health and Nutrition Examination Survey--I Epidemiologic Follow-up Study. New York: Oxford University Press; 1990. 320 p.

Courage UU, Stephen DP, Lucius IC, Ani C, Oche AO, Emmanuel AI, Olufemi AO. Prevalence of musculoskeletal diseases in a semi-urban Nigerian community: results of a cross-sectional survey using COPCORD methodology. Clin Rheumatol. 2017; 36(11): 2509-2516.

Croft P, Coggon D, Cruddas M, Cooper C. Osteoarthritis of the hip: an occupational disease in farmers. BMJ. 1992; 304(6837): 1269-72.

Cunningham LS, Kelsey JL. Epidemiology of musculoskeletal impairments and associated disability. Am J Public Health. 1984; 74(6): 574-9.

Danielsson L, Lindberg H, Nilsson BO. Prevalence of coxarthrosis. Clin Orthop Relat Res. 1984; 110-5.

Danielsson L, Lindberg H. Prevalence of coxarthrosis in an urban population during four decades. Clin Orthop Relat Res. 1997; 342: 106-10.

Davatchi F, Jamshidi A-R, Banihashemi AT, Gholami J, Forouzanfar MH, Akhlaghi M, Barghamdi M, Noorolahzadeh E, Khabazi A-R, Salesi M, Salari A-H, Karimifar M, Essalat-Manesh K, Hajialiloo M, Soroosh M, Farzad F, Moussavi H-R, Samadi F, Ghaznavi K, Asgharifard H, Zangiabadi A-H, Shahram F, Nadji A, Akbarian M, Gharibdoost F. WHO-ILAR COPCORD Study (Stage 1, Urban Study) in Iran. J Rheumatol. 2008; 35(7): 1384.

Duncan R, Francis RM, Collerton J, Davies K, Jagger C, Kingston A, Kirkwood T, Robinson L, Birrell F. Prevalence of arthritis and joint pain in the oldest old: findings from the Newcastle 85+ study. Age Ageing. 2011; 40(6): 752-5.

Forsberg K, Nilsson BE. Coxarthrosis on the island of Gotland. Increased prevalence in a rural population. Acta Orthop Scand. 1992; 63(1): 1-3.

Gosvig KK, Jacobsen S, Sonne-Holm S, Palm H, Troelsen A. Prevalence of Malformations of the Hip Joint and Their Relationship to Sex, Groin Pain, and Risk of Osteoarthritis: A Population-Based Survey. J Bone Joint Surg Am. 2010; 92(5): 1162-9.

Grubber JM, Callahan LF, Helmick CG, Zack MM, Pollard RA. Prevalence of radiographic hip and knee osteoarthritis by place of residence. J Rheumatol. 1998; 25(5): 959-63.

Guillemin F, Rat AC, Mazieres B, Pouchot J, Fautrel B, Euller-Ziegler L, Fardellone P, Morvan J, Roux CH, Verrouil E, Saraux A, Coste J, 3000 Osteoarthritis group. Prevalence of symptomatic hip and knee osteoarthritis: a two-phase population-based survey. Osteoarthr Cartil. 2011; 19(11): 1314-22.

Heliövaara M, Mäkelä M, Impivaara O, Knekt P, Aromaa A, Sievers K. Association of overweight, trauma and workload with coxarthrosis. A health survey of 7,217 persons. Acta Orthop Scand. 1993; 64(5): 513-8.

Iidaka T, Muraki S, Akune T, Oka H, Kodama R, Tanaka S, Kawaguchi H, Nakamura K, Yoshimura N. Prevalence of radiographic hip osteoarthritis and its association with hip pain in Japanese men and women: the ROAD study. Osteoarthr Cartil. 2016; 24(1): 117-123.

Indian Council of Medical Research (ICMR), Ministry of Health and Family Welfare (India), Vardhman Mahavir Medical College and Safdarjung Hospital (India). India Survey on Musculoskeletal Conditions 2007-2010.

Ingvarsson T. Prevalence and inheritance of hip osteoarthritis in Iceland. Acta Orthop Scand Suppl. 2000; 1-46.

Jacobsen S, Sonne-Holm S. Hip dysplasia: a significant risk factor for the development of hip osteoarthritis. A cross-sectional survey. Rheumatology (Oxford). 2005; 44(2): 211-8.

Joshi VL, Chopra A. Is there an urban-rural divide? Population surveys of rheumatic musculoskeletal disorders in the Pune region of India using the COPCORD Bhigwan model. J Rheumatol. 2009; 36(3): 614-22.

Juhakoski R, Heliövaara M, Impivaara O, Kröger H, Knekt P, Lauren H, Arokoski JPA. Risk factors for the development of hip osteoarthritis: a population-based prospective study. Rheumatology (Oxford). 2009; 48(1): 83-7.

Kim C, Linsenmeyer KD, Vlad SC, Guermazi A, Clancy MM, Niu J, Felson DT. Prevalence of Radiographic and Symptomatic Hip Osteoarthritis in an Urban United States Community: the Framingham Osteoarthritis Study. Arthritis Rheumatol. 2014; 66(11): 3013-7.

Loyola-Sanchez A, Richardson J, Pelaez-Ballestas I, Alvarez-Nemegyei J, Lavis JN, Wilson MG, Wilkins S. The impact of arthritis on the physical function of a rural Maya-Yucateco community and factors associated with its prevalence: a cross sectional, community-based study. Clin Rheumatol. 2016; 35 Suppl 1: 25-34.

Mannoni A, Briganti MP, Di Bari M, Ferrucci L, Costanzo S, Serni U, Masotti G, Marchionni N. Epidemiological profile of symptomatic osteoarthritis in older adults: a population based study in Dicomano, Italy. Ann Rheum Dis. 2003; 62(6): 576-8.

Moss AS, Murphy LB, Helmick CG, Schwartz TA, Barbour KE, Renner JB, Kalsbeek W, Jordan JM. Annual incidence rates of hip symptoms and three hip OA outcomes from a U.S. population-based cohort study: the Johnston County Osteoarthritis Project. Osteoarthr Cartil. 2016; 24(9): 1518-1527.

Nevitt MC, Xu L, Zhang Y, Lui L-Y, Yu W, Lane NE, Qin M, Hochberg MC, Cummings SR, Felson DT. Very low prevalence of hip osteoarthritis among Chinese elderly in Beijing, China, compared with whites in the United States: the Beijing osteoarthritis study. Arthritis Rheum. 2002; 46(7): 1773-9.

Nüesch E, Dieppe P, Reichenbach S, Williams S, Iff S, Jüni P. All cause and disease specific mortality in patients with knee or hip osteoarthritis: population based cohort study. BMJ. 2011; 342: d1165.

Odding E, Valkenburg HA, Algra D, Vandenouweland FA, Grobbee DE, Hofman A. Associations of radiological osteoarthritis of the hip and knee with locomotor disability in the Rotterdam Study. Ann Rheum Dis. 1998; 57(4): 203-8.

Oliveria SA, Felson DT, Reed JI, Cirillo PA, Walker AM. Incidence of symptomatic hand, hip, and knee osteoarthritis among patients in a health maintenance organization. Arthritis Rheum. 1995; 38(8): 1134-41.

Plotnikoff R, Karunamuni N, Lytvyak E, Penfold C, Schopflocher D, Imayama I, Johnson ST, Raine K. Osteoarthritis prevalence and modifiable factors: a population study. BMC Public Health. 2015; 15: 1195.

Pogrund H, Rutenberg M, Makin M, Robin G, Menczel J, Steinberg R. Osteoarthritis of the hip joint and osteoporosis: a radiological study in a random population sample in Jerusalem. Clin Orthop Relat Res. 1982; 130-5.

Prieto-Alhambra D, Judge A, Javaid MK, Cooper C, Diez-Perez A, Arden NK. Incidence and risk factors for clinically diagnosed knee, hip and hand osteoarthritis: influences of age, gender and osteoarthritis affecting other joints. Ann Rheum Dis. 2013; 73(9): 1659–64.

Quintana JM, Arostegui I, Escobar A, Azkarate J, Goenaga JI, Lafuente I. Prevalence of knee and hip osteoarthritis and the appropriateness of joint replacement in an older population. Arch Intern Med. 2008; 168(14): 1576-84.

Quintana JM, Escobar A, Arostegui I, Bilbao A, Armendariz P, Lafuente I, Agirre U. Prevalence of symptoms of knee or hip joints in older adults from the general population. Aging Clin Exp Res. 2008; 20(4): 329-36.

Reijman M, Hazes JMW, Bierma-Zeinstra SMA, Koes BW, Christgau S, Christiansen C, Uitterlinden AG, Pols HAP. A new marker for osteoarthritis: cross-sectional and longitudinal approach. Arthritis Rheum. 2004; 50(8): 2471-8.

Roux CH, Saraux A, Mazieres B, Pouchot J, Morvan J, Fautrel B, Testa J, Fardellone P, Rat AC, Coste J, Guillemin F, Euller-Ziegler L, KHOALA Osteoarthritis Group. Screening for hip and knee osteoarthritis in the general population: predictive value of a questionnaire and prevalence estimates. Ann Rheum Dis. 2008; 67(10): 1406-11.

Salaffi F, De Angelis R, Grassi W, MArche Pain Prevalence, INvestigation Group (MAPPING) study. Prevalence of musculoskeletal conditions in an Italian population sample: results of a regional community-based study. I. The MAPPING study. Clin Exp Rheumatol. 2005; 23(6): 819-28.

Solomon L, Beighton P, Lawrence JS. Osteoarthrosis in a rural South African Negro population. Ann Rheum Dis. 1976; 35(3): 274-8.

Tepper S, Hochberg MC. Factors associated with hip osteoarthritis: data from the First National Health and Nutrition Examination Survey (NHANES-I). Am J Epidemiol. 1993; 137(10): 1081-8.

Turkiewicz A, Petersson IF, Björk J, Hawker G, Dahlberg LE, Lohmander LS, Englund M. Current and future impact of osteoarthritis on health care: a population-based study with projections to year 2032. Osteoarthr Cartil. 2014; 22(11): 1826-32.

Van Saase JL, van Romunde LK, Cats A, Vandenbroucke JP, Valkenburg HA. Epidemiology of osteoarthritis: Zoetermeer survey. Comparison of radiological osteoarthritis in a Dutch population with that in 10 other populations. Ann Rheum Dis. 1989; 48(4): 271-80.

Wilson MG, Michet CJ Jr, Ilstrup DM, Melton LJ 3rd. Idiopathic symptomatic osteoarthritis of the hip and knee: a population-based incidence study. Mayo Clin Proc. 1990; 65(9): 1214-21.

Zeng QY, Chen R, Xiao ZY, Huang S-B, Liu Y, Xu JC, Chen SL, Darmawan J, Couchman KG, Wigley RD, Muirden KD. Low prevalence of knee and back pain in southeast China; the Shantou COPCORD study. J Rheumatol. 2004; 31(12): 2439-43.

Zhang J, Song L, Wei J, Zhang A, Dong H, Wen H, Luo J, Liu G. Prevalence of and risk factors for the occurrence of symptomatic osteoarthritis in rural regions of Shanxi Province, China. Int J Rheum Dis. 2016; 19(8): 781-789.

Clinical data sources

Truven Health Analytics. United States MarketScan Claims and Medicare Data - 2000. Ann Arbor, United States: Truven Health Analytics.

Truven Health Analytics. United States MarketScan Claims and Medicare Data - 2010–2014. Ann Arbor, United States: Truven Health Analytics.

Knee osteoarthritis

Akinpelu A, Alonge T, Adekanla B, Odole A. Prevalence and pattern of symptomatic knee osteoarthritis in Nigeria: a community based study. Internet J Allied Health Sci Pract. 2009; 7(3): 1-7.

American University of Beirut. Lebanon Community Oriented Programme for Control of Rheumatic Diseases Survey 2008.

Andersen RE, Crespo CJ, Ling SM, Bathon JM, Bartlett SJ. Prevalence of significant knee pain among older Americans: results from the Third National Health and Nutrition Examination Survey. J Am Geriatr Soc. 1999; 47(12): 1435-8.

Anderson JJ, Felson DT. Factors associated with osteoarthritis of the knee in the first national Health and Nutrition Examination Survey (HANES I). Evidence for an association with overweight, race, and physical demands of work. Am J Epidemiol. 1988; 128(1): 179-89.

Bagge E, Bjelle A, Valkenburg HA, Svanborg A. Prevalence of radiographic osteoarthritis in two elderly European populations. Rheumatol Int. 1992; 12(1): 33-8.

Branco JC, Rodrigues AM, Gouveia N, Eusébio M, Ramiro S, Machado PM, da Costa LP, Mourão AF, Silva I, Laires P, Sepriano A, Araújo F, Gonçalves S, Coelho PS, Tavares V, Cerol J, Mendes JM, Carmona L, Canhão H, EpiReumaPt study group. Prevalence of rheumatic and musculoskeletal diseases and their impact on health-related quality of life, physical function and mental health in Portugal: results from EpiReumaPt- a national health survey. RMD Open. 2016; 2(1): e000166.

Cakır N, Pamuk ÖN, Derviş E, Imeryüz N, Uslu H, Benian Ö, Elelçi E, Erdem G, Sarvan FO, Senocak M. The prevalences of some rheumatic diseases in western Turkey: Havsa study. Rheumatol Int. 2012; 32(4): 895-908.

Carmona L, Ballina J, Gabriel R, Laffon A, EPISER Study Group. The burden of musculoskeletal diseases in the general population of Spain: results from a national survey. Ann Rheum Dis. 2001; 60(11): 1040-5.

Chaiamnuay P, Darmawan J, Muirden KD, Assawatanabodee P. Epidemiology of rheumatic disease in rural Thailand: a WHO-ILAR COPCORD study. Community Oriented Programme for the Control of Rheumatic Disease. J Rheumatol. 1998; 25(7): 1382-7.

Cho HJ, Chang CB, Kim KW, Park JH, Yoo JH, Koh IJ, Kim TK. Gender and prevalence of knee osteoarthritis types in elderly Koreans. J Arthroplasty. 2011; 26(7): 994-9.

Cho HJ, Morey V, Kang JY, Kim KW, Kim TK. Prevalence and Risk Factors of Spine, Shoulder, Hand, Hip, and Knee Osteoarthritis in Community-dwelling Koreans Older Than Age 65 Years. Clin Orthop Relat Res. 2015; 473(10): 3307-3314.

Courage UU, Stephen DP, Lucius IC, Ani C, Oche AO, Emmanuel AI, Olufemi AO. Prevalence of musculoskeletal diseases in a semi-urban Nigerian community: results of a cross-sectional survey using COPCORD methodology. Clin Rheumatol. 2017; 36(11): 2509-2516.

Dans LF, Tankeh-Torres S, Amante CM, Penserga EG. The prevalence of rheumatic diseases in a Filipino urban population: a WHO-ILAR COPCORD Study. World Health Organization. International League of Associations for Rheumatology. Community Oriented Programme for the Control of the Rheumatic Diseases. J Rheumatol. 1997; 24(9): 1814-9.

Davis MA, Ettinger WH, Neuhaus JM, Hauck WW. Sex differences in osteoarthritis of the knee. The role of obesity. Am J Epidemiol. 1988; 127(5): 1019-30.

Dillon CF, Rasch EK, Gu Q, Hirsch R. Prevalence of knee osteoarthritis in the United States: arthritis data from the Third National Health and Nutrition Examination Survey 1991-94. J Rheumatol. 2006; 33(11): 2271-9.

Du H, Chen S-L, Bao C-D, Wang X-D, Lu Y, Gu Y-Y, Xu J-R, Chai W-M, Chen J, Nakamura H, Nishioka K. Prevalence and risk factors of knee osteoarthritis in Huang-Pu District, Shanghai, China. Rheumatol Int. 2005; 25(8): 585-90.

Ettinger WH, Davis MA, Neuhaus JM, Mallon KP. Long-term physical functioning in persons with knee osteoarthritis from NHANES. I: Effects of comorbid medical conditions. J Clin Epidemiol. 1994; 47(7): 809-15.

Farooqi A, Gibson T. Prevalence of the major rheumatic disorders in the adult population of north Pakistan. Br J Rheumatol. 1998; 37(5): 491-5.

Felson DT, Naimark A, Anderson J, Kazis L, Castelli W, Meenan RF. The prevalence of knee osteoarthritis in the elderly. The Framingham Osteoarthritis Study. Arthritis Rheum. 1987; 30(8): 914-8.

Felson DT, Zhang Y, Hannan MT, Naimark A, Weissman BN, Aliabadi P, Levy D. The incidence and natural history of knee osteoarthritis in the elderly. The Framingham Osteoarthritis Study. Arthritis Rheum. 1995; 38(10): 1500-5.

Fernandez-Lopez JC, Laffon A, Blanco FJ, Carmona L, EPISER Study Group. Prevalence, risk factors, and impact of knee pain suggesting osteoarthritis in Spain. Clin Exp Rheumatol. 2008; 26(2): 324-32.

Granados Y, Cedeno L, Rosillo C, Berbin S, Azocar M, Molina ME, Lara O, Sanchez G, Pelaez-Ballestas I. Prevalence of musculoskeletal disorders and rheumatic diseases in an urban community in Monagas State, Venezuela: a COPCORD study. Clin Rheumatol. 2015; 34(5): 871-7.

Guevara-Pacheco S, Feicán-Alvarado A, Sanín LH, Vintimilla-Ugalde J, Vintimilla-Moscoso F, Delgado-Pauta J, Lliguisaca-Segarra A, Dután-Erráez H, Guevara-Mosquera D, Ochoa-Robles V, Cardiel MH, Peláez-Ballestas I. Prevalence of musculoskeletal disorders and rheumatic diseases in Cuenca, Ecuador: a WHO-ILAR COPCORD study. Rheumatol Int. 2016; 36(9): 1195-1204.

Hart DJ, Spector TD. The relationship of obesity, fat distribution and osteoarthritis in women in the general population: the Chingford Study. J Rheumatol. 1993; 20(2): 331-5.

Ho-Pham LT, Lai TQ, Mai LD, Doan MC, Pham HN, Nguyen TV. Prevalence of radiographic osteoarthritis of the knee and its relationship to self-reported pain. PLoS One. 2014; 9(4): e94563.

Indian Council of Medical Research (ICMR), Ministry of Health and Family Welfare (India), Vardhman Mahavir Medical College and Safdarjung Hospital (India). India Survey on Musculoskeletal Conditions 2007-2010.

Jiang L, Rong J, Zhang Q, Hu F, Zhang S, Li X, Zhao Y, Tao T. Prevalence and associated factors of knee osteoarthritis in a community-based population in Heilongjiang, Northeast China. Rheumatol Int. 2012; 32(5): 1189-95.

Jordan JM, Helmick CG, Renner JB, Luta G, Dragomir AD, Woodard J, Fang F, Schwartz TA, Abbate LM, Callahan LF, Kalsbeek WD, Hochberg MC. Prevalence of knee symptoms and radiographic and symptomatic knee osteoarthritis in African Americans and Caucasians: the Johnston County Osteoarthritis Project. J Rheumatol. 2007; 34(1): 172-80.

Kaçar C, Gilgil E, Urhan S, Arikan V, Dündar U, Oksüz MC, Sünbüloglu G, Yildirim C, Tekeoglu I, Bütün B, Apaydin A, Tuncer T. The prevalence of symptomatic knee and distal interphalangeal joint osteoarthritis in the urban population of Antalya, Turkey. Rheumatol Int. 2005; 25(3): 201-4.

Kang X, Fransen M, Zhang Y, Li H, Ke Y, Lu M, Su S, Song X, Guo Y, Chen J, Niu J, Felson D, Lin J. The high prevalence of knee osteoarthritis in a rural Chinese population: the Wuchuan osteoarthritis study. Arthritis Rheum. 2009; 61(5): 641-7.

Kim I, Kim HA, Seo Y-I, Song YW, Jeong J-Y, Kim DH. The prevalence of knee osteoarthritis in elderly community residents in Korea. J Korean Med Sci. 2010; 25(2): 293-8.

Kim IJ, Kim DH, Song YW, Guermazi A, Crema MD, Hunter DJ, Seo Y-I, Kim HA. The prevalence of periarticular lesions detected on magnetic resonance imaging in middle-aged and elderly persons: a cross-sectional study. BMC Musculoskelet Disord. 2016; 17: 186.

Laxafoss E, Jacobsen S, Gosvig KK, Sonne-Holm S. Case definitions of knee osteoarthritis in 4,151 unselected subjects: relevance for epidemiological studies: the Copenhagen Osteoarthritis Study. Skeletal Radiol. 2010; 39(9): 859-66.

Lee S, Kim T-N, Kim S-H. Sarcopenic obesity is more closely associated with knee osteoarthritis than is nonsarcopenic obesity: a cross-sectional study. Arthritis Rheum. 2012; 64(12): 3947-54.

Leyland KM, Hart DJ, Javaid MK, Judge A, Kiran A, Soni A, Goulston LM, Cooper C, Spector TD, Arden NK. The natural history of radiographic knee osteoarthritis: a fourteen-year population-based cohort study. Arthritis Rheum. 2012; 64(7): 2243-51.

Liu Y, Zhang H, Liang N, Fan W, Li J, Huang Z, Yin Z, Wu Z, Hu J. Prevalence and associated factors of knee osteoarthritis in a rural Chinese adult population: an epidemiological survey. BMC Public Health. 2016; 16: 94.

Livshits G, Zhai G, Hart DJ, Kato BS, Wang H, Williams FM, Spector TD. Interleukin-6 is a significant predictor of radiographic knee osteoarthritis: The Chingford study. Arthritis Rheum. 2009; 60(7): 2037-45.

Loyola-Sanchez A, Richardson J, Pelaez-Ballestas I, Alvarez-Nemegyei J, Lavis JN, Wilson MG, Wilkins S. The impact of arthritis on the physical function of a rural Maya-Yucateco community and factors associated with its prevalence: a cross sectional, community-based study. Clin Rheumatol. 2016; 35 Suppl 1: 25-34.

Muraki S, Oka H, Akune T, Mabuchi A, En-yo Y, Yoshida M, Saika A, Suzuki T, Yoshida H, Ishibashi H, Yamamoto S, Nakamura K, Kawaguchi H, Yoshimura N. Prevalence of radiographic knee osteoarthritis and its association with knee pain in the elderly of Japanese population-based cohorts: the ROAD study. Osteoarthr Cartil. 2009; 17(9): 1137-43.

Murphy LB, Moss S, Do BT, Helmick CG, Schwartz TA, Barbour KE, Renner J, Kalsbeek W, Jordan JM. Annual Incidence of Knee Symptoms and Four Knee Osteoarthritis Outcomes in the Johnston County Osteoarthritis Project. Arthritis Care Res (Hoboken). 2016; 68(1): 55–65.

Nishimura A, Hasegawa M, Kato K, Yamada T, Uchida A, Sudo A. Risk factors for the incidence and progression of radiographic osteoarthritis of the knee among Japanese. Int Orthop. 2011; 35(6): 839-43.

Nishimura A, Hasegawa M, Wakabayashi H, Yoshida K, Kato K, Yamada T, Uchida A, Sudo A. Prevalence and characteristics of unilateral knee osteoarthritis in a community sample of elderly Japanese: do fractures around the knee affect the pathogenesis of unilateral knee osteoarthritis?. J Orthop Sci. 2012; 17(5): 556-61.

Pal CP, Singh P, Chaturvedi S, Pruthi KK, Vij A. Epidemiology of Knee Osteoarthritis in India and Related Factors. Indian J Orthop. 2016; 50(5): 518-22.

Petersson IF, Boegård T, Saxne T, Silman AJ, Svensson B. Radiographic osteoarthritis of the knee classified by the Ahlbäck and Kellgren and Lawrence systems for the tibiofemoral joint in people aged 35-54 years with chronic knee pain. Ann Rheum Dis. 1997; 56(8): 493-6.

Plotnikoff R, Karunamuni N, Lytvyak E, Penfold C, Schopflocher D, Imayama I, Johnson ST, Raine K. Osteoarthritis prevalence and modifiable factors: a population study. BMC Public Health. 2015; 15: 1195.

Sudo A, Miyamoto N, Horikawa K, Urawa M, Yamakawa T, Yamada T, Uchida A. Prevalence and risk factors for knee osteoarthritis in elderly Japanese men and women. J Orthop Sci. 2008; 13(5): 413-8.

Tamm A, Lintrop M, Veske K, Hansen U, Tamm A. Prevalence of patello- and tibiofemoral osteoarthritis in Elva, Southern Estonia. J Rheumatol. 2008; 35(3): 543-4.

Tang X, Wang S, Zhan S, Niu J, Tao K, Zhang Y, Lin J. The Prevalence of Symptomatic Knee Osteoarthritis in China: Results From the China Health and Retirement Longitudinal Study. Arthritis Rheumatol. 2016; 68(3): 648-53.

Tehrani-Banihashemi A, Davatchi F, Jamshidi A-R, Faezi T, Paragomi P, Barghamdi M. Prevalence of osteoarthritis in rural areas of Iran: a WHO-ILAR COPCORD study. Int J Rheum Dis. 2014; 17(4): 384–388.

Thomas E, Peat G, Croft P. Defining and mapping the person with osteoarthritis for population studies and public health. Rheumatology (Oxford). 2014; 53(2): 338-345.

Toivanen AT, Heliövaara M, Impivaara O, Arokoski JPA, Knekt P, Lauren H, Kröger H. Obesity, physically demanding work and traumatic knee injury are major risk factors for knee osteoarthritis--a population-based study with a follow-up of 22 years. Rheumatology (Oxford). 2010; 49(2): 308-14.

Turkiewicz A, Gerhardsson de Verdier M, Engström G, Nilsson PM, Mellström C, Lohmander LS, Englund M. Prevalence of knee pain and knee OA in southern Sweden and the proportion that seeks medical care. Rheumatology (Oxford). 2015; 54(5): 827-35.

Verweij LM, Van Schoor NM, Deeg DJ, Dekker J, Visser M. Physical activity and incident clinical knee osteoarthritis in older adults. Arthritis Care Res. 2009; 61(2): 152-7.

Wang Y, Peng R, Ma R. Epidemiological investigation of osteoarthritis in middle-aged mongolian and senior residents of the inner mongolia autonomous region. Iran Red Crescent Med J. 2013; 15(10): e8303.

Wigley R, Manahan L, Muirden KD, Caragay R, Pinfold B, Couchman KG, Valkenburg HA. Rheumatic disease in a Philippine village. II: a WHO-ILAR-APLAR COPCORD study, phases II and III. Rheumatol Int. 1991; 11(4-5): 157-61.

Yeşil H, Hepgüler S, Öztürk C, Çapacı K, Yeşil M. Prevalence of Symptomatic Knee, Hand and Hip Osteoarthritis Among Individuals 40 Years or Older: a Study Conducted in İzmir City. Acta Orthop Traumatol Turc. 2013; 47(4): 231-5.

Yoshimura N, Muraki S, Oka H, Mabuchi A, En-Yo Y, Yoshida M, Saika A, Yoshida H, Suzuki T, Yamamoto S, Ishibashi H, Kawaguchi H, Nakamura K, Akune T. Prevalence of knee osteoarthritis, lumbar spondylosis, and osteoporosis in Japanese men and women: the research on osteoarthritis/osteoporosis against disability study. J Bone Miner Metab. 2009; 27(5): 620-8.

Zeng Q, Zang C, Li X, Dong H, Zhang A, Lin L. Associated risk factors of knee osteoarthritis: a population survey in Taiyuan, China. Chin Med J (Engl). 2006; 119(18): 1522-7.

Zeng S-Y, Gong Y, Zhang Y-P, Chen S-B, Chen J-Y, Lin C-Q, Peng J-H, Hou Z-D, Zhong J-Q, Liang H-J, Huang G-H, Wang D-M, Lai H-Y, Li L-P, Zeng QY. Changes in the Prevalence of Rheumatic Diseases in Shantou, China, in the Past Three Decades: A COPCORD Study. PLoS One. 2015; 10(9): e0138492.

Zhang J, Song L, Liu G, Zhang A, Dong H, Liu Z, Li X, Luo J. Risk factors for and prevalence of knee osteoarthritis in the rural areas of Shanxi Province, North China: a COPCORD study. Rheumatol Int. 2013; 33(11): 2783-2788.

Zhang Y, Xu L, Nevitt MC, Aliabadi P, Yu W, Qin M, Lui LY, Felson DT. Comparison of the prevalence of knee osteoarthritis between the elderly Chinese population in Beijing and whites in the United States: The Beijing Osteoarthritis Study. Arthritis Rheum. 2001; 44(9): 2065-71.

Clinical data sources

Truven Health Analytics. United States MarketScan Claims and Medicare Data - 2000. Ann Arbor, United States: Truven Health Analytics.

Truven Health Analytics. United States MarketScan Claims and Medicare Data - 2010–2014. Ann Arbor, United States: Truven Health Analytics.

Other osteoarthritis

Truven Health Analytics. United States MarketScan Claims and Medicare Data - 2000. Ann Arbor, United States: Truven Health Analytics.

Truven Health Analytics. United States MarketScan Claims and Medicare Data - 2010–2014. Ann Arbor, United States: Truven Health Analytics.

Contributions

Writing the first draft of the manuscript

Peter Brooks, Marita Cross, Jacek A Kopec, Lyn M March, Kanyin Liane Ong, Jaimie D Steinmetz, Theo Vos, and Anthony Woolf

Providing data or critical feedback on data sources

Aidin Abedi, Hubert Amu, Jalal Arabloo, Ayele Mamo Argaw, Anton A Artamonov, Tahira Ashraf, Amadou Barrow, Vijayalakshmi S Bhojaraja, Paul Svitil Briant, Vijay Kumar Chattu, Marita Cross, Garland T Culbreth, Xiaochen Dai, Lalit Dandona, Rakhi Dandona, Samath Dhamminda Dharmaratne, Meghnath Dhimal, Mostafa Dianatinasab, Karsten E Dreinhoefer, Marisa Freitas, Kai Fukutaki, Balasankar Ganesan, Tamiru Getachew, Ahmad Ghashghaee, Mahaveer Golechha, Vivek Kumar Gupta, Nima Hafezi-Nejad, Rabih Halwani, Netanja I Harlianto, Josep Maria Haro, Mehdi Hosseinzadeh, Ravi Prakash Jha, Jost B Jonas, Nitin Joseph, Himal Kandel, Yun Jin Kim, Oleksii Korzh, Vijay Krishnamoorthy, G Anil Kumar, Sang-woong Lee, Stephen S Lim, Lyn M March, Mohamed Kamal Mesregah, Erkin M Mirrakhimov, Awoke Misganaw, Reza Mohammadpourhodki, Ali H Mokdad, Sara Momtazmanesh, Henok Biresaw Netsere, Sandhya Neupane Kandel, Kanyin Liane Ong, Mayowa O Owolabi, Songhomitra Panda-Jonas, Anamika Pandey, Shrikant Pawar, Paolo Pedersini, Jeevan Pereira, Salman Rawaf, Reza Rawassizadeh, Seyed-

Mansoor Rayegani, Leonardo Roever, Basema Saddik, Milena M Santric-Milicevic, Masood Ali Shaikh, Mohammed Shannawaz, Jeevan K Shetty, Parnian Shobeiri, Ambrish Singh, Jasvinder A Singh, Mohammad Sadegh Soltani-Zangbar, Sahel Valadan Tahbaz, Pascual R Valdez, Bay Vo, Linh Gia Vu, Seyed Hossein Yahyazadeh Jabbari, Naohiro Yonemoto, and Ismaeel Yunusa.

Developing methods or computational machinery

Hubert Amu, Aleksandr Y Aravkin, Kaleb Coberly, Jessica A Cruz, Garland T Culbreth, Xiaochen Dai, Mostafa Dianatinasab, Kai Fukutaki, Mehdi Hosseinzadeh, Sang-woong Lee, Reza Mohammadpourhodki, Ali H Mokdad, Reza Rawassizadeh, Jaimie D Steinmetz, Bay Vo, and Stein Emil Vollset.

Providing critical feedback on methods or results

Aidin Abedi, Ilana N Ackerman, Hubert Amu, Benny Antony, Jalal Arabloo, Anton A Artamonov, Tahira Ashraf, Amadou Barrow, Lindsay M Bearne, Isabela M Bensenor, Alemshet Yirga Berhie, Nikha Bhardwaj, Pankaj Bhardwaj, Vijayalakshmi S Bhojaraja, Ali Bijani, Andrew M Briggs, Nadeem Shafique Butt, Jaykaran Charan, Vijay Kumar Chattu, Marita Cross, Garland T Culbreth, Omid Dadras, Xiaochen Dai, Lalit Dandona, Rakhi Dandona, Katie de Luca, Edgar Denova-Gutiérrez, Samath Dhamminda Dharmaratne, Meghnath Dhimal, Mostafa Dianatinasab, Karsten E Dreinhoefer, Muhammed Elhadi, Umar Farooque, Irina Filip, Florian Fischer, Marisa Freitas, Kai Fukutaki, Balasankar Ganesan, Belete Negese Belete Gemeda, Tamiru Getachew, Tiffany K Gill, Mahaveer Golechha, Davide Golinelli, Bhawna Gupta, Vivek Kumar Gupta, Rasool Haddadi, Nima Hafezi-Nejad, Lydia M Haile, Rabih Halwani, Samer Hamidi, Asif Hanif, Netanja I Harlianto, Josep Maria Haro, Jan Hartvigsen, Simon I Hay, Jeffrey J Hebert, Golnaz Heidari, Mohammad-Salar Hosseini, Mehdi Hosseinzadeh, Alexander Kevin Hsiao, Irena M Ilic, Milena D Ilic, Louis Jacob, Ravi Prakash Jha, Jost B Jonas, Nitin Joseph, Himal Kandel, Ibraheem M Karaye, Md Jobair Khan, Yun Jin Kim, Ali-Asghar Kolahi, Oleksii Korzh, Rajasekaran Koteeswaran, Jacek A Kopec, Vijay Krishnamoorthy, G Anil Kumar, Narinder Kumar, Sang-woong Lee, Stephen S Lim, Justin Lo, Stany W Lobo, Giancarlo Lucchetti, Mohammad-Reza Malekpour, Ahmad Azam Malik, Luiz Garcia Garcia Mandarano-Filho, Lyn M March, Santi Martini, Alexios-Fotios A Mentis, Mohamed Kamal Mesregah, Tomislav Mestrovic, Erkin M Mirrakhimov, Awoke Misganaw, Reza Mohammadpourhodki, Ali H Mokdad, Sara Momtazmanesh, Shane Douglas Morrison, Christopher J L Murray, Hasan Nassereldine, Henok Biresaw Netsere, Sandhya Neupane Kandel, Kanyin Liane Ong, Mayowa O Owolabi, Songhomitra Panda-Jonas, Anamika Pandey, Shrikant Pawar, Paolo Pedersini, Jeevan Pereira, Amir Radfar, Mohammad-Mahdi Rashidi, David Laith Rawaf, Salman Rawaf, Reza Rawassizadeh, Seyed-Mansoor Rayegani, Daniela Ribeiro, Leonardo Roever, Basema Saddik, Sana Salehi, Francesco Sanmarchi, Milena M Santric-Milicevic, Saeed Shahabi, Masood Ali Shaikh, Elaheh Shaker, Mohammed Shannawaz, Rajendra Sharma, Saurab Sharma, Jeevan K Shetty, Rahman Shiri, Parnian Shobeiri, Diego Augusto Santos Silva, Ambrish Singh, Jasvinder A Singh, Surjit Singh, Søren T Skou, Amanda E Smith, Mohammad Sadegh Soltani-Zangbar, Antonina V Starodubova, Jaimie D Steinmetz, Sahel Valadan Tahbaz, Pascual R Valdez, Bay Vo, Stein Emil Vollset, Linh Gia Vu, Yuan-Pang Wang, Seyed Hossein Yahyazadeh Jabbari, Naohiro Yonemoto, and Ismaeel Yunusa.

Drafting the work or revising it critically for important intellectual content

Mohsen Abbasi-Kangevari, Aidin Abedi, Ilana N Ackerman, Hubert Amu, Benny Antony, Jalal Arabloo, Amadou Barrow, Lindsay M Bearne, Isabela M Bensenor, Vijayalakshmi S Bhojaraja, Andrew M Briggs, Nadeem Shafique Butt, Vijay Kumar Chattu, Flavia M Cicuttini, Marita Cross, Garland T Culbreth, Katie de

Luca, Edgar Denova-Gutiérrez, Samath Dhamminda Dharmaratne, Meghnath Dhimal, Mostafa Dianatinasab, Karsten E Dreinhoefer, Muhammed Elhadi, Hamid Reza Farpour, Irina Filip, Florian Fischer, Marisa Freitas, Balasankar Ganesan, Seyyed-Hadi Ghamari, Tiffany K Gill, Davide Golinelli, Bhawna Gupta, Veer Bala Gupta, Vivek Kumar Gupta, Nima Hafezi-Nejad, Rabih Halwani, Netanja I Harlianto, Josep Maria Haro, Jan Hartvigsen, Simon I Hay, Jeffrey J Hebert, Golnaz Heidari, Mohammad-Salar Hosseini, Alexander Kevin Hsiao, Irena M Ilic, Milena D Ilic, Louis Jacob, Ranil Jayawardena, Ravi Prakash Jha, Jost B Jonas, Nitin Joseph, Himal Kandel, Md Jobair Khan, Yun Jin Kim, Jacek A Kopec, Oleksii Korzh, Narinder Kumar, Giancarlo Lucchetti, Mohammad-Reza Malekpour, Ahmad Azam Malik, Lyn M March, Alexios-Fotios A Mentis, Mohamed Kamal Mesregah, Tomislav Mestrovic, Awoke Misganaw, Reza Mohammadpourhodki, Ali H Mokdad, Sara Momtazmanesh, Shane Douglas Morrison, Christopher J L Murray, Hasan Nassereldine, Henok Biresaw Netsere, Sandhya Neupane Kandel, Kanyin Liane Ong, Mayowa O Owolabi, Songhomitra Panda-Jonas, Shrikant Pawar, Paolo Pedersini, Jeevan Pereira, Amir Radfar, Quinn Rafferty, David Laith Rawaf, Salman Rawaf, Seyed-Mansoor Rayegani, Daniela Ribeiro, Leonardo Roever, Basema Saddik, Amirhossein Sahebkar, Lidia Sanchez Riera, Francesco Sanmarchi, Milena M Santric-Milicevic, Saeed Shahabi, Elaheh Shaker, Mohammed Shannawaz, Saurab Sharma, Jeevan K Shetty, Parnian Shobeiri, Diego Augusto Santos Silva, Jasvinder A Singh, Søren T Skou, Helen Slater, Antonina V Starodubova, Arash Tehrani-Banihashemi, Sahel Valadan Tahbaz, Stein Emil Vollset, Linh Gia Vu, Yuan-Pang Wang, Seyed Hossein Yahyazadeh Jabbari, and Naohiro Yonemoto.

Managing the estimation or publications process

Peter Brooks, Hailey Hagins, Simon I Hay, Jacek A Kopec, Lyn M March, Ali H Mokdad, Christopher J L Murray, Kanyin Liane Ong, Jaimie D Steinmetz, Theo Vos, and Anthony Woolf.
